# Atlas of tissue renin-angiotensin-aldosterone system in human: A transcriptomic meta-analysis

**DOI:** 10.1038/srep10035

**Published:** 2015-05-20

**Authors:** Ali Nehme, Catherine Cerutti, Nedra Dhaouadi, Marie Paule Gustin, Pierre-Yves Courand, Kazem Zibara, Giampiero Bricca

**Affiliations:** 1 EA4173, Functional genomics of arterial hypertension, Hôpital Nord-Ouest, Villefranche-sur-Saône; Université Lyon1, Lyon, France; 2 ER045, Laboratory of stem cells, Department of Biology, Faculty of sciences, Lebanese University, Beirut, Lebanon

## Abstract

Tissue renin-angiotensin-aldosterone system (RAAS) has attracted much attention because of its physiological and pharmacological implications; however, a clear definition of tissue RAAS is still missing. We aimed to establish a preliminary atlas for the organization of RAAS across 23 different normal human tissues. A set of 37 genes encoding classical and novel RAAS participants including gluco- and mineralo-corticoids were defined as extended RAAS (extRAAS) system. Microarray data sets containing more than 10 normal tissues were downloaded from the GEO database. R software was used to extract expression levels and construct dendrograms of extRAAS genes within each data set. Tissue co-expression modules were then extracted from reproducible gene clusters across data sets. An atlas of the maps of tissue-specific organization of extRAAS was constructed from gene expression and coordination data. Our analysis included 143 data sets containing 4933 samples representing 23 different tissues. Expression data provided an insight on the favored pathways in a given tissue. Gene coordination indicated the existence of tissue-specific modules organized or not around conserved core groups of transcripts. The atlas of tissue-specific organization of extRAAS will help better understand tissue-specific effects of RAAS. This will provide a frame for developing more effective and selective pharmaceuticals targeting extRAAS.

Since the identification of renin by Tigerstedt and Bergmann in 1898, the renin-angiotensin-aldosterone system (RAAS) has been extensively studied. It is a major therapeutic target in cardiovascular diseases (CVD) due to its important role in maintaining cellular and tissue physiology^1^,^2^.

In its classical endocrine view, angiotensinogen (AGT), produced by the liver, is cleaved in the plasma by the tightly regulated renin, produced by the kidney. This results in the release of the amino terminus decapeptide angiotensin I (1–10) (Ang I). Ang I is further processed by the angiotensin converting enzyme (ACE) which releases the active (1–8) octapeptide angiotensin II (Ang II). The latter binds to its specific membrane receptors and elicit cellular effects. The system is currently characterized by an increased complexity, with the discovery of new functional components such as the receptors for renin, for the heptapetide angiotensin (1–7) and for the hexapeptide angiotensin IV (3–8), in addition to the enzymes leading to the production of active angiotensin peptides from Ang I. Until recently, renin was thought to be the rate limiting factor for the production of these active peptides due to its high specificity and affinity for angiotensinogen. However, the recent discovery of the angiotensin (1–12) peptide as a potential alternative of Ang I for cleavage by ACE, chymase or neprilysin raised the possibility of alternative renin-independent pathway(s) for the generation of active peptides from AGT^3^,^4^. Moreover, the known activity of cathepsin D, cathepsin G and tissue kallikrein to directly accept angiotensinogen, as a substrate to release Ang I or Ang II, further strengthens this hypothesis^5^. Altogether, this leads to the concept of tissue RAAS that was shown to act at the paracrine and autocrine levels, independently from the circulating RAAS.

Tissue RAAS has attracted much attention because of its physiological, pharmacological and therapeutic implications^6^. In fact, tissue RAAS is often investigated in the context of expression of specific enzymes or receptors, pharmacological responses to specific peptides, or pharmacological inhibition of specific enzymes. However, very few studies simultaneously compared several components of RAAS in several tissues^7^,^8^. We have compared the expression of several components of RAAS in the atheroma plaque relative to nearby low grade remodeling tissue. Indeed, we found that a specific pattern of expression modifications of both receptors and enzymes was found to be associated with the remodeling process^9^,^10^. Moreover, we showed that the trans-differentiation of vascular smooth muscle cells (VSMCs) could establish a positive loop between angiotensin II and corticosteroids signaling, thus functionally linking both systems^11^. In addition, this suggested that along with the expression levels, correlations between transcripts could hold a tissue- or process-specific property.

Based on literature and results obtained in our laboratory, we defined an extended renin-angiotensin-aldosterone system (extRAAS) which includes 37 gene products^3^,^11^,^12^,^13^,^14^,^15^. The extRAAS system contains the classical systemic RAAS participants (AGT-REN-ACE-AGTR1) in addition to novel enzymes and receptors^13^,^16^ described at the tissue level (Fig. 1, see also Supplementary Table S1).

Our hypothesis is that a tissue-specific extRAAS organization should refer to the co-expression of genes coding for specific subsets of potential participants. In this study, we aimed to address the organization of extRAAS components in several human tissues. Owing to the availability of large public transcriptomic databases, we established the first atlas of tissue extRAAS in a large set of human tissues. Using this atlas, we showed that tissue specificity could be achieved through a specific pattern of expression and coordination of transcripts.

## Material and Methods

### Microarray data sets

**Figure 2:**
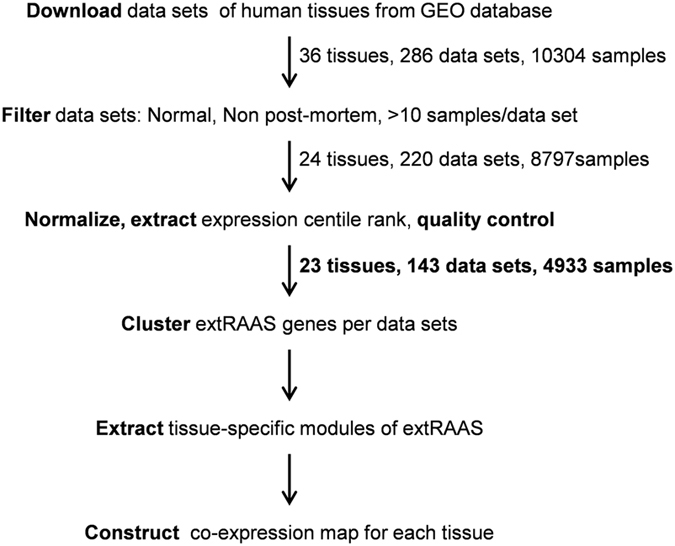
Experimental workflow . Microarray data sets obtained from tissue samples were downloaded from the Gene Expression Omnibus (GEO) database; then filtered for normal samples based on exclusion criteria. The data sets passing quality control were selected and their expression data were normalized by centile rank transformation. Each of the data sets was then submitted for extRAAS hierarchical clustering and expression profiling. The resulting dendrograms were then used to assess the level of reproducibility of the different clusters across different data sets obtained from the same tissue. Genes that were most often clustered together in different data sets of the same tissue were annotated as tissue-specific modules. For each tissue, a co-expression map was elaborated using both expression level and tissue-specific module belonging of each extRAAS gene.

Published microarray data sets of different human tissues were downloaded from the Gene Expression Omnibus database (http://www.ncbi.nlm.nih.gov/geo/). Data sets were then filtered for normal tissues, by excluding cell culture samples, post mortem tissues, diseased tissues (cancer or other), and tissues from pharmacologically treated individuals. Age, gender, and ethnicity were not taken into account in selecting the data sets. Only data sets with more than 10 normal samples were retained. Affymetrix microarray data sets were exclusively selected and only those containing all the probe sets were included for further analysis. The detailed procedure is shown in Fig. 2.

### Expression level and quality control

After filtering, data sets were checked for the expression distribution of their individual samples. Data sets which showed large variability among samples were then eliminated. Data sets were normalized by their authors using different methods including robust multichip average (RMA), GC-RMA or a global score method^17^. Since data sets were obtained from different experiments, the data sets lacking any transformation were log2-transformed. In order to compare expression data between different data sets, the centile rank of a gene was calculated using R-software by normalizing its mean expression level relative to the mean expression data distribution over the microarray. As a quality control step to remove outliers, data sets of a given tissue were then hierarchically clustered based on the obtained centile rank of extRAAS gene expression (Cluster 3.0 software using the average linkage method, http://bonsai.hgc.jp/~mdehoon/software/cluster/, and Java TreeView 3.0, http://jtreeview.sourceforge.net free software tools)^18^. Non-clustered data sets were then eliminated from the study.

### ExtRAAS expression profiles across tissues and tissue dendrogram

In order to reflect the relative abundance of extRAAS transcripts in a given tissue, the mean expression centile rank (MCR) of genes was calculated across data sets. After log transformation of MCR, a tissue dendrogram was built by hierarchical clustering of tissues based on the correlation between MCR profiles of extRAAS (Cluster 3.0 and TreeView 3.0). Principle component analysis (PCA) was also applied on tissues based on standardized MCR values, using the R software (ade4 package). Projection of tissues on the 3 principal axes (rgl package) was used to disclose specific groups of tissues^19^.

### Clustering of extRAAS genes per data set

The R software was used for statistical description and clustering of the 37 extRAAS gene transcripts in each data set, using the “Cluster” R library. ExtRAAS gene transcripts were hierarchically clustered in each data set using Pearson correlation distance and Ward’s agglomeration method^20^. Each of the obtained dendrograms was then cut at a given level to identify the gene clusters. The cut-off level was chosen on the basis of a balance between the level of clustering strength, assessed with the agglomerative coefficient and a minimum of 3 gene transcripts per cluster.

### Identifying local extRAAS co-expression modules

For a given tissue, a co-expression module was defined as a set of 2 or more genes that were coordinated across data sets. The average coordination rate of genes within a module was calculated as the average percentage of coordinated genes within a module that were always clustered together across the different data sets in a specific tissue. A threshold of > 55% was the criterion used to define gene modules that were representative for a specific tissue.

### Statistical analysis

For centile rank expression levels, one MCR value was computed per tissue and one mean MCR for all tissues. These MCR values were presented as (1) mean ± SD to show intra- and inter-tissue variation in extRAAS gene expression and (2) mean ± SEM to describe specific gene expression.

## Results

### Microarray data sets

Following filtering and applying the exclusion criteria, normalization of the data sets for normal tissues was done as described in Fig. 2. After quality control, 77 outlier data sets were removed from a total of 220. The retained 143 data sets contained a total of 4933 samples corresponding to 23 different tissues (Table 1, see detailed list in Supplementary Table S2). These tissues belong to different systems and have different physiological functions and embryological origins. The total number of data sets was variable between tissues and ranged between 2 (thyroid) and 17 (whole blood), whereas the total number of samples per tissue ranged between 54 (embryo) and 774 (whole blood). The average number of data sets per tissue was 6 ± 4, whereas that of samples per tissue was 214 ± 178. Some tissues, such as adrenal gland, vascular wall or brain, were absent from this study because it was impossible to obtain non post-mortem normal samples from these tissues.

### ExtRAAS gene expression

**Figure 3:**
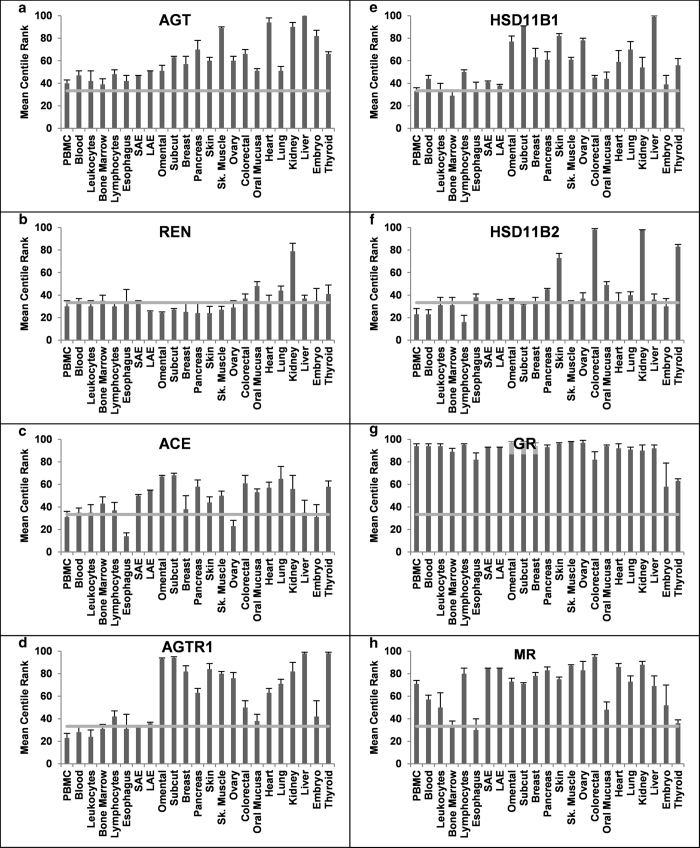
mRNA expression profile of classical RAAS and Corticosteroid system (COS) across tissues. The relative abundance of gene transcripts in each tissue is expressed as the mean expression centile rank (MCR) across data sets (Mean ± SEM). Classical RAAS genes (**a**-**d**): AGT, angiotensinogen; REN, renin; ACE, angiotensin converting enzyme; AGTR1, angiotensin II type 1 receptor. COS genes (**e**-**h**): HSD11B1, 11beta hydroxysteroid dehydrogenase type 1; HSD11B2, 11beta hydroxysteroid dehydrogenase type 2; GR, glucorticoid receptor; MR, mineralocorticoid receptor; PBMC, peripheral blood mononuclear cells; SAE, small airways epithelium; LAE, large airways epithelium; Omental, Omental adipose tissue; Subcut, sub-cutaneous adipose tissue; Sk. Muscle, Skeletal muscle.

**Figure 4:**
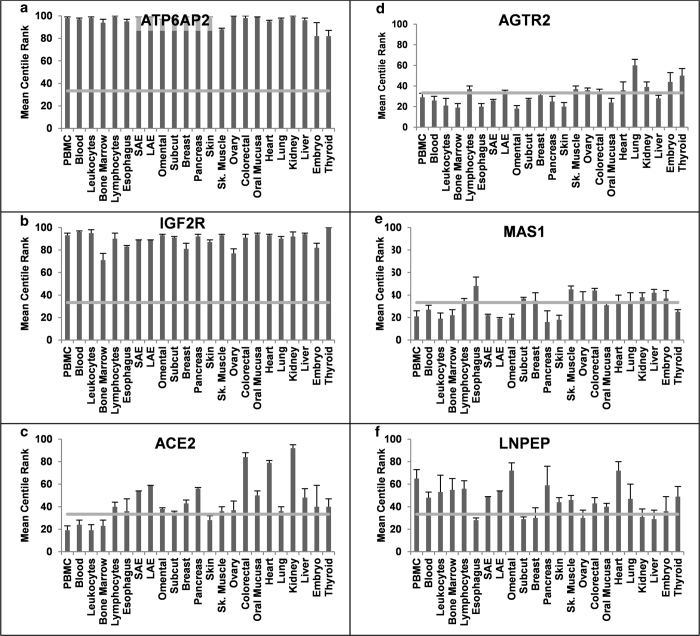
mRNA expression profile of key components of extRAAS across tissues. The relative abundance of gene transcripts in each tissue is expressed as the mean expression centile rank (MCR) across data sets (Mean ± SEM). (**a-b**). Renin receptors: ATP6AP2, ATPase, H + transporting, lysosomal accessory protein 2; IGF2R, insulin-like growth factor 2 receptor. **c**. ACE2, angiotensin converting enzyme type 2. (**d-e**). Angiotensin peptides receptors: AGTR2, angiotensin II type 2 receptor; MAS1, Ang (1–7) receptor; LNPEP, angiotensin IV receptor. PBMC, peripheral blood mononuclear cells; SAE, small airways epithelium; LAE, large airways epithelium; Omental, Omental adipose tissue; Subcut, sub-cutaneous adipose tissue; Sk. Muscle, Skeletal muscle. Expression profiles for the other investigated tissues are provided in supplemental data.

Among the 37 extRAAS genes, neurolysin peptidase (NLN) was excluded from the analysis since it was not represented by any probe set in most of the microarray platforms. The MCR expression level of the remaining 36 extRAAS genes in each tissue was then calculated as the mean centile rank (MCR) of a gene transcript across data sets; thus supplying a complete and comparative assessment of mRNA abundance across tissues (Supplementary Table S3 and Supplementary Fig. S1). Using the MCR data, distribution of gene expression across tissues displayed the previously known classical RAAS features. The highest expression levels of key markers were found in their respective tissues^1^, such as AGT in the liver, renin in the kidney, and ACE in the lung (Fig. 3a, 3b and 3c, respectively). Moreover, aldosterone sensitive tissues such as the kidney and the colon, along with skin and thyroid gland, contained the highest levels of HSD11B2 transcript (Fig. 3f). The MCR data revealed novel features for other extRAAS gene expression. For instance, the glucocorticoid receptor (GR) and the two potential prorenin and renin receptors, ATP6AP2 and IGF2R, were among the most abundant mRNAs in all tissues (Figs. 3g, 4a and 4b, respectively). Tissue-specific features could also be identified for the first time at the signal response level through AGTR2, MAS1, LNPEP-IRAP (Fig. 4d–f), GPER and EGFR (see Supplementary Fig. S1). In fact, MAS1 (Fig. 4e) and ACE2 (Fig. 4c) were highly expressed in the kidney and skeletal muscle while the LNPEP-IRAP (Fig. 4f) receptor was abundantly present in the omental adipose tissue, heart and pancreas, but not in the kidney. Similarly, this systematic comparison demonstrated new features such as the higher level of AGTR2 mRNA (Fig. 4d) in the large airways epithelium (bronchi) compared to small airways epithelium (bronchioles). On the other hand, HSD11B2 was expressed at relatively low levels in both types of airway tissues (Fig. 3f). Notably, lymphocytes were the only circulating blood cells found to contain high amounts of all angiotensin, renin and mineralocorticoid receptors mRNAs.

### Classification of tissues according to extRAAS expression

**Figure 5:**
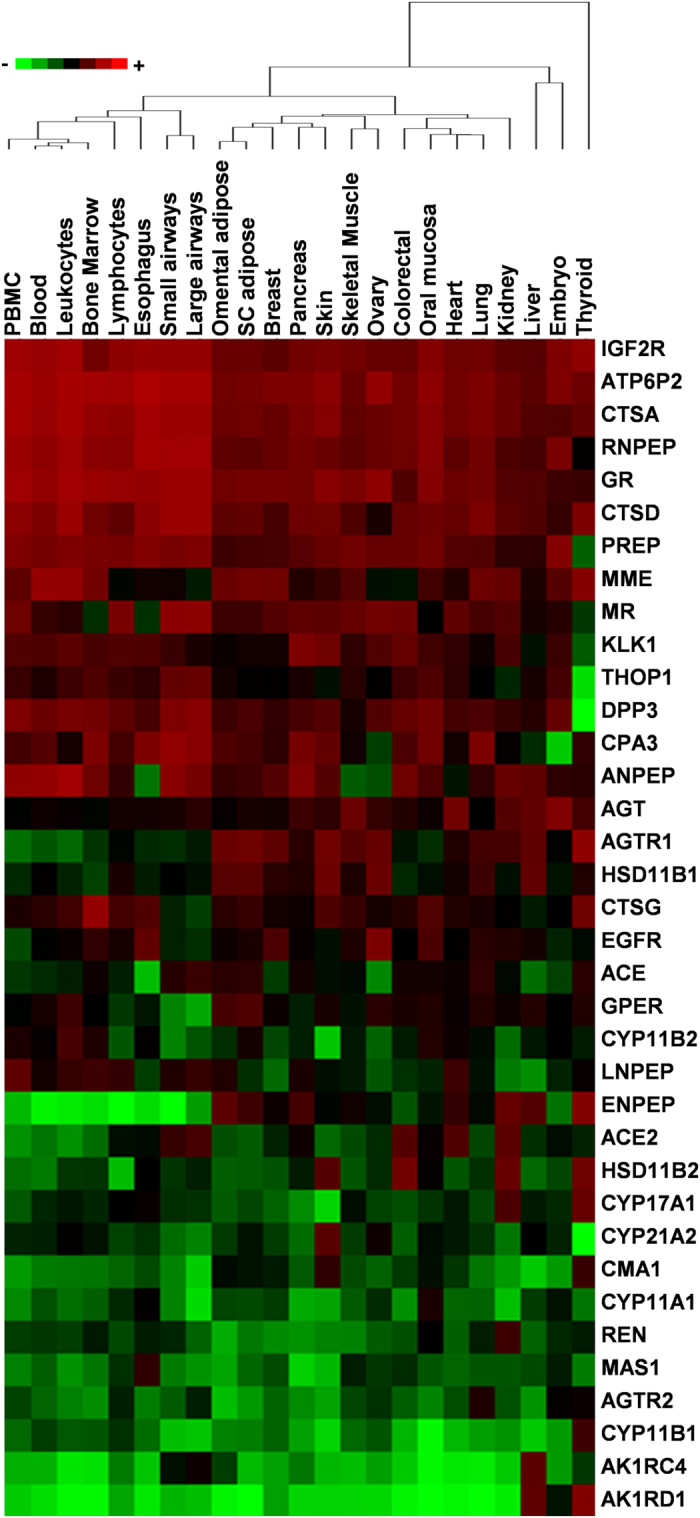
ExtRAAS-based tissue clustering. The tissue dendrogram was drawn based on the average linkage method (cluster 3.0 software) using the logged and normalized mean centile expression rank of extRAAS genes. Colors of the heatmap correspond to the relative log (MCR) in each tissue. PBMC, peripheral blood mononuclear cells.

Tissue dendrogram was drawn using MCR of extRAAS genes per tissue (Fig. 5). Interestingly, tissues belonging to the same system were clustered together. For example, peripheral blood mononuclear cells, whole blood cells, leukocytes and lymphocytes were found to be grouped with bone marrow. In addition, the epithelia from large and small respiratory airways were very close, as were omental and subcutaneous adipose tissues. On the other hand, the thyroid gland showed an extremely different expression profile and was not clustered with any of the other tissues. Finally, aldosterone-sensitive tissues (e.g. skin, colorectal and kidney), found to have high levels of HSD11B2 mRNAs, were not closely clustered. Similar results were obtained using PCA (data not shown).

### ExtRAAS gene clustering in each data set

Hierarchical clustering of extRAAS genes in each data set indicated that all 36 genes could be strongly clustered with a mean agglomerative coefficient above 0.7, by default between 0 and 1, for all of the data sets in all tissues except lymphocytes, skeletal muscles and small airways. This showed that a clustering structure clearly exists within extRAAS transcripts.

### ExtRAAS genes co-expression modules

Local extRAAS modules of co-expressed genes were then identified by calculating the average coordination rates of gene sets across data sets within tissues. Table 2 shows extRAAS co-expression modules and the corresponding gene expression levels for the kidney, heart, skin, and omental adipose tissues. A minimum of 5 modules per tissue was found in the kidney, omental adipose and total blood tissues, and a maximum of 8 modules was found in 10 tissues including the thyroid gland, liver, lung and subcutaneous adipose tissues (Supplementary Table S4). The largest module, comprising 11 transcripts, was found in the kidney. This module contained AGT, REN, ACE and ACE2 along with transcripts of other enzymes involved in the metabolism of angiotensin.

By comparing the modules in the different tissues, we found 3 types of transcript groups: (1) the first type comprised modules that were based on the presence of a “core group” of transcripts correlated in more than 50% of tissues. A total of 3 such modules were isolated, 2 of which were proteolytic enzymes modules. The first proteolytic module is based on CTSA and CTSD core group. These 2 transcripts were found to be coordinated with other proteolytic enzymes in numerous tissues, including the kidney. In fact, these 2 transcripts were coordinated with 9 other transcripts in the kidney and omental adipose tissue, making them the two largest modules detected. This module never contained receptors with the exception of the pancreas where both prorenin-renin receptors, ATP6AP2 and IGF2R, together with GR were associated (Supplementary Table S4). The second module of proteolytic enzymes was based on the core group of CPA3, CTSG, and CMA1 transcripts, which were often clustered together without any other genes (Table 2). This module was typically found in the subcutaneous adipose tissue, pancreas and skin. Interestingly, this module lacks CMA1 in the heart, which is replaced by ACE and included AGTR1. The third core group-based module contained receptor-coding transcripts: AGTR1, MR and GR (Table 2 and Supplementary Table S4). This cluster of receptors often contained only GR and MR.

(2) The second type of transcripts group constituted co-expression modules detected only in a single tissue. For example, only the heart tissue contained the IGF2R-MME-HSD11B1-AKR1D1 module (Table 2). At least one such module was detected in each tissue (Supplementary Table S4).

(3) The last type of transcripts group comprised the non-clustered transcripts. Their number could vary according to tissues, ranging between 4 in the kidney and up to 22 in the skin. Each of the extRAAS transcripts was found in this group in at least one tissue, except omental adipose which had all extRAAS genes included in co-expression modules.

### ExtRAAS tissue atlas

**Figure 6:**
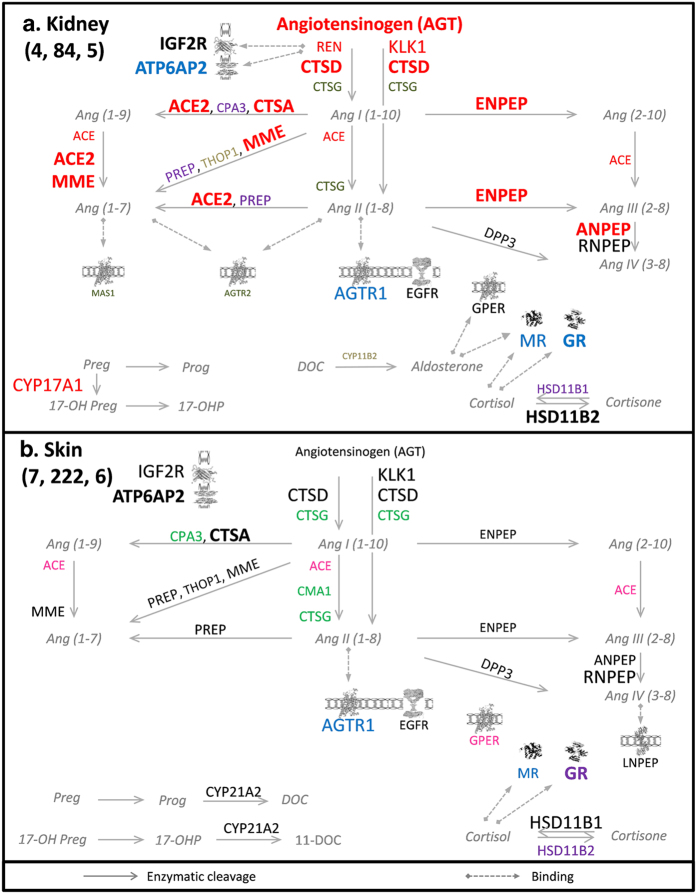
ExtRAAS maps in the kidney (a) and the skin (b). The number of data sets, samples and modules are represented between brackets (data sets, samples, modules) below tissue name in the upper left corner of the figure. Gene transcripts are represented by the corresponding official symbols. Genes are represented based on their coordination (same color = same module) and mean centile expression rank (MCR, different font size). Non-clustered genes are represented in black color. Gene transcripts below the first tertile (MCR < 33.3) in each tissue were excluded for simplicity. Angiotensin peptides and corticosteroid metabolites are represented in gray italics. Images of IGF2R^36^, ATP6AP2^37^, MR^38^, GR^39^, G-protein coupled receptors (AGTR1, AGTR2, GPER and MAS1)^40^ and LNPEP^41^ were obtained from the Protein Data Bank in Europe (PDBe) with respective PDBe IDs: 2YDO, 3LBS, 1P93, 4P8Q, 2AA2. Image of EGFR^42^ was obtained from Protein Data Bank DOI:10.2210/ rcsb_pdb/mom_2010_6. Maps for the other investigated tissues are provided in supplemental data.

extRAAS maps were built for each tissue using expression levels and co-expression modules (Supplementary Atlas S1). Degradation pathways leading to angiotensin peptides with no known activity, such as angiotensin (5–10) and angiotensin (1–5) pathways, in addition to the angiotensin (1–12) pathway, were not included in the maps. These maps clearly displayed different transcriptional organization between tissues, with only few strong similarities. As shown in Fig. 6, although the kidney and the skin are both aldosterone sensitive tissues linked to water and salt homeostasis, their extRAAS maps showed different patterns of expression and coordination. Not only different expression patterns were observed, with the absence of MAS1, AGTR2, ACE2, REN and CYP17A1 transcripts in the skin compared to the kidney, but also the transcripts present in both tissues had different patterns of co-expression. The kidney showed a large CTSA-CTSD-based module associating highly expressed proteolytic enzymes and including the highly expressed AGT transcript (Fig. 6a, genes in red). In contrast, none of the genes of the red module in the kidney was found to be coordinated in the skin. On the other hand, the smaller proteolytic CTSG-CPA3-CMA1 module was present in the skin, but not in the kidney (Fig. 6b, genes in green). Similarly, the AGTR1-MR-GR-based receptor module was present in the kidney (Fig. 6a, genes in blue), but not in the skin.

**Figure 7:**
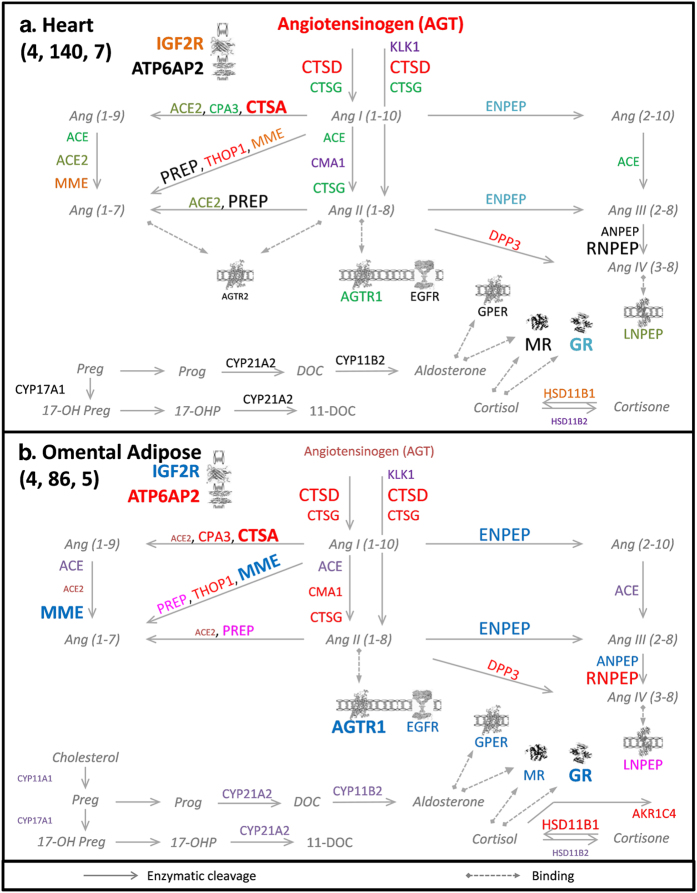
ExtRAAS maps in the heart (a) and the omental adipose tissue (b). The number of data sets, samples and modules are represented between brackets (data sets, samples, modules) below tissue name in the upper left corner of the figure. Gene transcripts are represented by the corresponding official symbols. The genes are represented based on their coordination (same color = same module) and mean centile expression rank (MCR, different font size). Non-clustered genes are represented in black color. Gene transcripts below the first tertile (MCR < 33.3) in each tissue were excluded for simplicity. Angiotensin peptides and corticosteroid metabolites are represented in gray italics. Images of IGF2R^36^, ATP6AP2^37^, MR^38^, GR^39^, G-protein coupled receptors (AGTR1, GTR2, GPER and MAS1)^40^ and LNPEP^41^ were obtained from the Protein Data Bank in Europe (PDBe) with respective PDBe IDs: 2YDO, 3LBS, 1P93, 4P8Q, 2AA2. Image of EGFR^42^ was obtained from Protein Data Bank DOI:10.2210/rcsb_pdb/mom_2010_6. Maps for the other investigated tissues are provided in the supplemental data.

In the same way, both the heart and the adipose tissues, which are known for their active local RAAS, showed abundant levels of angiotensin metabolizing enzymes and receptors mRNAs. However, there were large differences in clustering patterns between both tissues. In the heart, the CTSG and CPA3 core transcripts were coordinated with ACE, rather than CMA1 (Fig. 7a). In addition, the CTSA-CTSD proteolytic module was present in the heart (Fig. 7a), including the AGT transcript and two other enzymes transcripts DPP3 and THOP1). On the other hand, the CTSA-CTSD and the CTSG-CPA3-CMA1 proteolytic modules were combined in the omental adipose tissue (Fig. 7b), forming a larger module that included up to 11 gene transcripts. Moreover, the omental adipose tissue had the largest receptor-containing module which included the GR-MR-AGTR1 core group, GPER, IGF2R and EGFR gene transcripts, in addition to three enzyme-coding transcripts, MME, ENPEP and ANPEP. On the contrary, co-expression of receptor-coding gene transcripts was not detected in the heart.

Although similarities in patterns of expression can be detected between tissues, co-expression similarities were mainly limited to the core group-transcripts. For instance, the omental and adipose tissue were very similar in their expression patterns; however, they had very different patterns of coordination.

## Discussion

In this study, we propose for the first time an extensive atlas of the mRNA organization of extRAAS across 23 different normal human tissues. These maps were generated using a large amount of publicly available transcriptomic data in combination with a statistical meta-analysis, based on hierarchical clustering. Using expression levels and coordination of genes, tissue maps were generated for all 23 tissues. These maps displayed the tissue-specific features and may represent a reference for the analysis of pathological situations. Indeed, we showed that tissue specificity of extRAAS may be achieved through a specific pattern of expression and coordination of transcripts. When comparing the different maps, it appears that tissue-specific co-expression patterns are achieved through the combination of: (1) tissue-specific patterns of mRNA abundance; (2) modules of co-expressed transcripts; and (3) a specific combination of expression and coordination patterns.

Because this study was performed only at the mRNA level, it exclusively explored the gene expression properties of cells composing each tissue. It indicated the existence, at the mRNA level, of tissue-specific modules organized or not around core groups of transcripts. Two such core groups were enzymatic groups of peptidases suggesting that their coordinated expression could exert a strong effect in orienting the metabolism of angiotensin I. The other core group was a receptor group involving GR-MR with AGTR1 which may orient cell sensitivity. It is important to note that the substrate AGT mRNA is abundant in almost all tissues, as previously reported^21^. However, it is clustered only in the kidney and the heart, where it associates within the CTSA-CTSD module. The key quantitative role of AGT gene expression in determining blood pressure has been demonstrated both in humans and animals^22^,^23^. Our maps suggest that this effect may be associated with increased activity of specific components of the extRAAS in the heart and kidney tissues while the increased AGT expression in other tissues would fuel independent enzymatic pathways.

For each tissue, the meta-analysis included 2 to 17 data sets fulfilling the inclusion criteria. The number of data sets and the number of observations greater than 10 within each data set allowed robust estimation of gene expression levels and robust identification of co-expression modules. The mapping was found to fit perfectly with most known tissue distribution of transcript levels when a threshold was applied at the first tertile of the microarray expression distribution (MCR < 33 taken as non-expressed, Supplementary Atlas S1)^3^,^24^. In addition, we provide here a primary information in tissues where only scarce information was available, such as the ovary, thyroid gland, pancreas, skeletal muscle, circulating cells, and airways epithelia^25^,^26^ (Supplementary Atlas S1). Interestingly, bone marrow cells showed almost the same map as total blood cells, leukocytes, or peripheral blood mononuclear cells indicating that the transcriptional coordination may be preserved during “cell lineage”. Moreover, although the expression patterns were similar in subcutaneous and omental fat, there were important differences between the coordination patterns of both these tissues. This suggests that the differences observed between the two adipose tissues in obese patients^27^ may likely be due to local differences in expression regulatory mechanisms.

All tissues appeared to have abundant mRNAs coding for GR and the two potential prorenin-renin receptors ATP6AP2 and IGFR2. Receptors mRNAs were all found to be abundant only in colorectal mucosa, skeletal muscle and lymphocytes. In all other tissues, at least one angiotensin peptide receptor was expressed confirming the very high tissue specificity of the responses through the combined activation of the different subsets of receptors. Interestingly, although there was often a strong coordination between angiotensin signaling receptors and steroid receptors, the metabolic pathways appeared to be structured only for the angiotensin proteases, with rare association with one or the other enzymes of the steroid pathway. The maps also suggest that the “active” metabolic pathways could lead to a dead-end with no receptors for peptides such as LNPEP-IRAP receptor in the kidney, or MAS1 receptor in the heart.

Altogether our results suggest that the extRAAS signaling pathways are regulated at the mRNA level in different tissues according to the 3 following targets that seem to be independent. First, the substrate AGT had scarce and limited coodination, except in the heart and kidney, suggesting that it is involved in other independent regulation(s). Second, the signal generation where the peptidic cascades showed 2 almost independent coordinated modules around CTSA-CTSD and CTSG-CMA1-CPA3, possibly orienting peptide flow. Third, the cell response where there was a strong core group of receptors GR-MR-AGTR1 which provide cell sensitivity. Although strong inter-relationships have been previously described for receptors^28^,^29^,^30^,^31^, it is the first time that these relationships are detected for extRAAS enzymes. A major difference also appeared between peptide and steroid hormones. While the peptidic angiotensin cascade displayed high tissue organization, few and dispersed coordination was observed in the steroid biosynthetic pathway. Most of the local steroid synthesis regulation seemed to rely not only on CYP11B1 and CYP11B2 but mostly on both HSD11B1 and HSD11B2. On the other hand, the complete aldosterone synthetic pathway was present in adipose tissue, as expected from Biones *et al.*^32^, as well as most of the features of local corticoid generation and metabolism^33^. Finally, the non-clustered transcripts, or those with dispersed coordination across tissues, were also of interest because they could represent bottle necks in the pathways and/or be linked to other cellular functions or pathways.

The identified modules of gene transcripts may hold a great functional importance. Their correlations may result from tissue and intercellular properties, but also from intracellular properties. It has been hypothesized from transcriptomic analysis that co-expressed genes may share common regulation either on the transcriptional side or the RNA degradation one. Indeed, we recently showed, in the field of TGFβ regulation in the human arterial wall, that the coordination between transcripts could be reproduced in cell culture as the result of common transcription factors activation^34^. Using a different approach, Zhou *et al.*^35^ recently showed, in a human proximal tubular cell line, that different ligands of the Wnt/β-catenin pathway could stimulate the expression of several classical RAAS genes simultaneously. Indeed, all these transcripts were included in the large specific module identified in the kidney. This raises several questions, first about the fate of other members of the coordinated groups in the cellular model, and second about the role of β-catenin pathway in the coordination observed *in situ*. Whatever the responses are, this strengthens the hypothesis that gene co-expression observed *in situ* has a cellular origin, and that it may result from the actions of transcription factors, which can be identified and tested.

In conclusion, our meta-analysis made possible the emergence of conserved results for each tissue and across data sets that are robust by definition. This study allowed extracting three levels of information. First, the expression levels revealed the features of the “endocrine RAAS” and permitted to get new insights of tissue distribution among the alternative players, such as MAS1, prorenin and renin receptors, and LNPEP-IRAP. A second level of information was the identification of core modules of transcripts that were robustly identified in several tissues, such as CTSA-CTSD, CTSG-CMA1-CPA3 and GR-MR-AGTR1. These clusters seemed to dissociate signal production from signal reception pathways, and could also orient the peptide cascade. A third level was about tissue-specific coordination of extRAAS transcripts, which built up by combining tissue-specific clusters, with modification and/or combination of the core modules.

The atlas we have established in this study provides the basis for further more elaborate studies that would take into account the variability in each tissue, due to age, gender or ethnicity. In addition, cellular and molecular mechanisms within this organization need to be elucidated, as well as how they translate into enzymatic activity, peptide production and signaling. Finally, the extensive atlas of the extRAAS organization across normal human tissues that we propose here will considerably help understand the tissue-specific effects of extRAAS and of its targeting drugs.

## Additional Information

**How to cite this article**: Nehme, A. *et al*. Atlas of tissue renin-angiotensin-aldosterone system in human: A transcriptomic meta-analysis. *Sci. Rep.* **5**, 10035; doi: 10.1038/srep10035 (2015).

## Supplementary Material

Supplementary Information

## Figures and Tables

**Figure 1:**
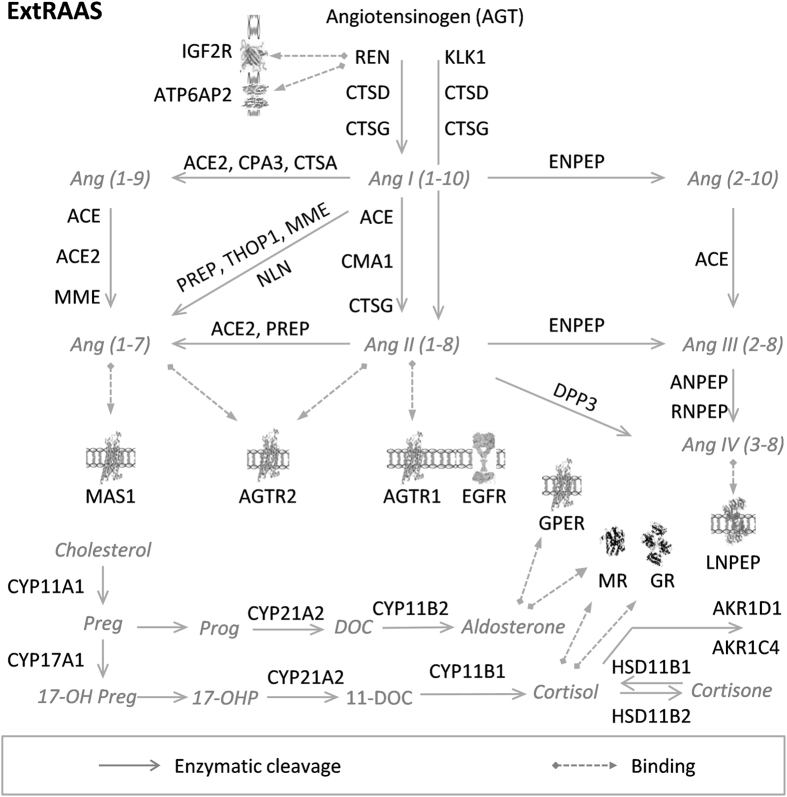
Extended Renin Angiotensin Aldosterone System (ExtRAAS). The metabolic cascades of angiotensin peptides, and cortico-and gluco-corticoid pathways have been represented using symbols of genes coding for the substrate, the enzymes and the receptors involved in the pathway. Angiotensin peptides and steroid hormones are represented in grey using their usual abbreviation. Ang, Angiotensin; Preg, Pregnanolone; Prog, Progesterone; DOC, deoxycortisol; 17-OHP, 17-OH Progesterone; ACE, angiotensin I converting enzyme; ACE2, angiotensin I converting enzyme type 2; AGTR1, angiotensin II type 1 receptor; AGTR2, angiotensin II type 2 receptor; AKRIC4, aldo-keto reductase family 1, member C4; AKRID1, aldo-keto reductase family 1, member D1; ANPEP, alanyl-aminopeptidase; ATP6AP2, prorenin/renin receptor; CMA1, chymase 1; CPA3, carboxypeptidase A3; CTSA, cathepsin A; CTSD, cathepsin D; CTSG, cathepsin G; CYP11A1, cytochrome P450, family 11, subfamily A, polypeptide 1; CYP11B1, cortisol synthase; CYP11B2, aldosterone synthase; CYP17A1, cytochrome P450, family 17, subfamily A, polypeptide 1; CYP21A2, cytochrome P450 enzyme, family 21, subfamily A, polypeptide 2; DPP3, dipeptidyl-peptidase 3; ENPEP, glutamyl aminopeptidase (aminopeptidase A); GR, glucocorticoid receptor; HSD11B1, hydroxysteroid (11-beta) dehydrogenase 1; HSD11B2, hydroxysteroid (11-beta) dehydrogenase 2; IGF2R , insulin-like growth factor 2 receptor; KLK1, tissue kallikrein; LNPEP, leucyl/cystinylaminopeptidase; MAS1, MAS1 proto-oncogene; MME, membrane metallo-endopeptidase; MR, mineralocorticoid receptor; NLN, neurolysin (metallopeptidase M3 family); PREP, prolylendopeptidase; REN, renin; RNPEP, arginyl aminopeptidase (aminopeptidase B); THOP1, thimetoligopeptidase 1. Images of IGF2R^36^, ATP6AP2^37^, MR^38^, GR^39^, G-protein coupled receptors (AGTR1, AGTR2, GPER and MAS1)^40^ and LNPEP^41^ were obtained from the Protein Data Bank in Europe (PDBe) with respective PDBe IDs: 2YDO, 3LBS, 1P93, 4P8Q, 2AA2. Image of EGFR^42^ was obtained from Protein Data Bank DOI:10.2210/rcsb_pdb/mom_2010_6.

**Table 1:** List of the studied human tissues.

**Organ system**	**Tissue**	**Data sets**	**Samples**
Urinary system	Kidney	4	84
Cardiovascular system	Heart	4	140
Adipose tissue	Sub-cutaneous adipose	9	474
	Omental adipose	4	86
Respiratory system	Large airways epithelium	5	101
	Small airways epithelium	8	357
	Lung	5	210
Reproductive system	Ovary	5	55
Fetal	Embryo	3	54
Digestive system	Colorectum	8	171
	Esophagus	3	83
	Liver	5	93
	Pancreas	3	100
	Oral mucosa	4	193
Blood	Lymphocytes	4	142
	Leukocytes	4	222
	PBMC	11	303
	Whole Blood	17	774
Other organ systems	Skin	7	222
	Thyroid	2	66
	Skeletal muscle	14	556
	Breast	6	239
	Bone marrow stem cells	8	208
Total	23	143	4933

The final list of data sets obtained after filtering for normal samples and quality control. All selected data sets were obtained on the Affymetrix microarrays platform.

**Table 2:** ExtRAAS tissue modules.

**Tissues (data sets, samples)**	**Module 1**		**Module 2**		**Module 3**		**Module 4**		**Module 5**		**Module 6**	
**Kidney (4, 84)**	84%		88%		85%		94%		80%			
	**CTSA**	**99**	ATP6AP2	99	**CTSG**	**59**	THOP1	48	PREP	74		
	ANPEP	98	**GR**	**90**	AGTR2	39	CYP11B2	34	**CPA3**	**60**		
	ENPEP	97	**MR**	**88**	MAS1	38	CYP21A2	32	HSD11B1	54		
	MME	97	**AGTR1**	**82**	AKR1C4	19	**CMA1**	**26**	LNPEP	31		
	ACE2	92			AKR1D1	11			CYP11A1	21		
	**CTSD**	**92**										
	AGT	90										
	CYP17A1	85										
	KLK1	84										
	REN	75										
	ACE	56										
**Heart (4, 140)**	77%		75%		80%		100%		81%		81%	
	**CTSA**	**94**	**GR**	**92**	**CTSG**	**64**	EGFR	54	KLK1	68	IGF2R	93
	AGT	94	ENPEP	69	**AGTR1**	**63**	REN	33	**CMA1**	**41**	MME	64
	**CTSD**	**88**			**CPA3**	**59**	MAS1	32	HSD11B2	35	HSD11B1	59
	DPP3	74			ACE	57			CYP11B1	20	AKR1D1	8
	THOP1	67			AKR1C4	11						
**Skin (7, 222)**	81%		57%		90%		71%		57%		71%	
	GPER	54	AGTR1	84	**CPA3**	**78**	THOP1	44	**GR**	**96**	ATP6AP2	98
	ACE	44	**MR**	**75**	**CTSG**	**70**	REN	24	HSD11B2	73	ACE2	28
	CYP11B2	16			**CMA1**	**60**						
**Omental adipose (4, 86)**	91%		83%		83%		85%		75%			
	ATP6AP2	96	**GR**	**97**	ACE	67	AGT	51	PREP	74		
	**CTSA**	**95**	MME	96	KLK1	57	ACE2	38	LNPEP	72		
	**CTSD**	**89**	**AGTR1**	**93**	CYP11B2	46	REN	24				
	RNPEP	86	IGF2R	93	CYP21A2	44	MAS1	20				
	HSD11B1	77	ENPEP	88	CYP11A1	37	AKR1D1	9				
	**CPA3**	**75**	GPER	75	HSD11B2	37						
	DPP3	72	**MR**	**73**	CYP17A1	36						
	**CTSG**	**65**	ANPEP	67	CYP11B1	33						
	THOP1	62	EGFR	67	AGTR2	18						
	**CMA1**	**51**										
	AKR1C4	49										

This table represents extRAAS co-expression modules (module 1–6) in the kidney, heart, skin and omental adipose tissues (data sets, samples). At the top of each module the average coordination rate is expressed as a percentage shown at the top of each module (average percentage of genes within a module that are always coordinated across the different data sets of a specific tissue). The mRNA abundance of each gene is present next to the gene symbol and is expressed in the mean MCR (mean centile rank, the percent level of the transcript within the transcriptome). Core-groups transcripts are in bold.
