# Supplementary material for: Atlas of tissue renin-angiotensin-aldosterone system in human: A transcriptomic meta-analysis
Source: Sci Rep. 2015 May 20;5:10035. doi: 10.1038/srep10035 (PMC4445654; doi:10.1038/srep10035)
Supplement: Supplementary Information [file srep10035-s1.pdf]

ATLAS OF TISSUE RENIN-ANGIOTENSIN-ALDOSTERONE SYSTEM IN HUMAN: A TRANSCRIPTOMIC META-ANALYSIS

Supplemental data

Ali Nehme, Catherine Cerutti, Nedra Dhaouadi, Marie Paule Gustin, Pierre-Yves Courand, Kazem Zibara*, Giampiero Bricca*.

* Co-last and corresponding authors

Address correspondence to:

* Dr Giampiero BRICCA, EA4173, Functional genomics of arterial hypertension, Université Lyon1, Domaine Rockefeller, 8 avenue Rockefeller, France; email: giampiero.bricca@inserm.fr

*Dr Kazem ZIBARA, ER045, Laboratory of stem cells, Department of Biology, Faculty of Sciences, Lebanese University, Beirut – Lebanon; email: kzibara@ul.edu.lb

Table of Contents

#	Title	Page
Supplementary table S1	Extended Renin-Angiotensin-Aldosterone System (ExtRAAS) genes	3
Supplementary table S2	Detailed Inventory of Studied Datasets	5
Supplementary table S3	Tissue Expression Profiles of ExtRAAS Genes	11
Supplementary table S4	ExtRAAS Co-expression Modules in All 23 Studied Tissues	15
Supplementary Figure S1	ExtRAAS Genes Expression Profiles Across Studied Tissues	22
Supplementary Atlas S1	ExtRAAS Maps in All Studied Tissues	28
	Atlas template	29
	Thyroid	30
	Embryo	31
	Liver	32
	Kidney	33
	Lung	34
	Heart	35
	Oral Mucosa	36
	Colorectal	37
	Ovary	38
	Skeletal Muscle	39
	Skin	40
	Pancreas	41
	Breast	42
	Subcutaneous Adipose	43
	Omental Adipose	44
	Large Airways Epithelium	45
	Small Airways Epithelium	46
	Esophagus	47
	Lymphocytes	48
	Bone Marrow	49
	Leukocytes	50
	Total blood	51
	PBMC	52

Supplementary Table S1: Extended renin-angiotensin-aldosterone system (ExtRAAS) gene

Supplementary Table S1: Extended renin-angiotensin-aldosterone system (ExtRAAS) gene. ExtRAAS constitute 37 genes; 25 encode for the renin-angiotensin system (RAS) components corresponding to angiotensinogen (AGT) 17 enzymes and 7 receptors; and 12 genes encode for corticosteroid system (COS) proteins corresponding to 9 enzymes and 3 receptors. Classical RAAS genes are annotated by an asterisk. RAS, renin-angiotensin system; COS, corticosteroid system; GeneID, gene refseq ID.

System	Gene Symbol	Gene Description	GeneID
RAS	ACE*	angiotensin I converting enzyme (peptidyl-dipeptidase A) 1	1636
RAS	ACE2	angiotensin I converting enzyme (peptidyl-dipeptidase A) 2	59272
RAS	AGT*	angiotensinogen (serpin peptidase inhibitor, clade A, member 8)	183
RAS	AGTR1*	angiotensin II receptor, type 1	185
RAS	AGTR2	angiotensin II receptor, type 2	186
RAS	ANPEP	alanyl (membrane) aminopeptidase	290
RAS	ATP6AP2	ATPase, H+ transporting, lysosomal accessory protein 2	10159
RAS	CMA1	chymase 1, mast cell	1215
RAS	CPA3	carboxypeptidase A3 (mast cell)	1359
RAS	CTSA	cathepsin A	5476
RAS	CTSD	cathepsin D	1509
RAS	CTSG	cathepsin G	1511
RAS	DPP3	dipeptidyl-peptidase 3	10072
RAS	EGFR	epidermal growth factor receptor	1956
RAS	ENPEP	glutamyl aminopeptidase (aminopeptidase A)	2028
RAS	IGF2R	insulin-like growth factor 2 receptor	3482
RAS	KLK1	kallikrein 1	3816
RAS	LNPEP	leucyl/cystinyl aminopeptidase	4012
RAS	MAS1	MAS1 oncogene	4142
RAS	MME	membrane metallo-endopeptidase	4311
RAS	NLN	neurolysin (metallopeptidase M3 family)	57486
RAS	PREP	prolyl endopeptidase	5550
RAS	REN*	renin	5972
RAS	RNPEP	arginyl aminopeptidase (aminopeptidase B)	6051
RAS	THOP1	thimet oligopeptidase 1	7064
COS	AKR1C4	aldo-keto reductase family 1, member C4	1109
COS	AKR1D1	aldo-keto reductase family 1, member D1	6718
COS	CYP11A1	cytochrome P450, family 11, subfamily A, polypeptide 1	1583
COS	CYP11B1	cytochrome P450, family 11, subfamily B, polypeptide 1	1584
COS	CYP11B2*	cytochrome P450, family 11, subfamily B, polypeptide 2	1585
COS	CYP17A1	cytochrome P450, family 17, subfamily A, polypeptide 1	1586
COS	CYP21A2	cytochrome P450, family 21, subfamily A, polypeptide 2	1589
COS	GPER	G protein-coupled estrogen receptor 1	2852
COS	HSD11B1	hydroxysteroid (11-beta) dehydrogenase 1	3290
COS	HSD11B2*	hydroxysteroid (11-beta) dehydrogenase 2	3291
COS	NR3C1	nuclear receptor subfamily 3, group C, member 1 (glucocorticoid receptor)	2908
COS	NR3C2*	nuclear receptor subfamily 3, group C, member 2	4306

Supplementary Table S2: Detailed Inventory of Studied Datasets

Supplementary Table S2: Detailed inventory of studied datasets. Dataset ID: data set accession number in Gene expression omnibus (GEO) database; Platform ID: GEO accession number of platform; species: species from which tissues where obtained; Normal: number of normal samples in the dataset; Algorithm: normalization method used by authors; Cut height: the level at which the dendrogram was cut; MAC: mean agglomerative coefficient of dendrogram.

Dataset ID	Platform ID	Species	Tissue	Normal	algorithm	Cut height	MAC
GSE18723	GPL96	Homo Sapiens	B cells	40	MAS 5	0.9	0.47
GSE16028	GPL570	Homo Sapiens	Blood	109	Quantile	0.82	0.48
GSE46480	GPL570	Homo Sapiens	Blood	98	Quantile	0.767	0.74
GSE10041	GPL570	Homo Sapiens	Blood	72	Global scaling	0.8	0.66
GSE19743	GPL570	Homo Sapiens	Blood	63	MBEI	0.8	0.63
GSE33580	GPL570	Homo Sapiens	Blood	43	MAS 5	0.89	0.57
GSE26049	GPL570	Homo Sapiens	Blood	42	RMA	0.9	0.67
GSE27263	GPL570	Homo Sapiens	Blood	26	RMA	0.82	0.79
GSE45536	GPL570	Homo Sapiens	Blood	24	RMA	0.987	0.71
GSE19314	GPL570	Homo sapiens	Blood	21	RMA	0.88	0.68
GSE22255	GPL570	Homo sapiens	Blood	20	RMA	0.93	0.71
GSE28750	GPL570	Homo Sapiens	Blood	20	RMA	0.9	0.8
GSE37171	GPL570	Homo Sapiens	Blood	20	RMA	0.8	0.8
GSE33341	GPL571	Homo sapiens	Blood	43	RMA	1	0.73
GSE21592	GPL571	Homo sapiens	Blood	20	RMA	1	0.83
GSE47199	GPL6244	Homo Sapiens	Blood	21	RMA	0.76	0.85
GSE12288	GPL96	Homo sapiens	Blood	112	MAS 5.0	0.785	0.56
GSE14795	GPL96	Homo Sapiens	Blood	20	MAS 5.0	0.94	0.62
GSE45460	GPL6244	Homo Sapiens	BM cells	31	RMA	1	0.84
GSE11504	GPL570	Homo Sapiens	BM cells	25	RMA	1	0.79
GSE16334	GPL96	Homo Sapiens	BM cells	11	RMA	1.02	0.84
GSE32719	GPL570	Homo Sapiens	BM cells	27	RMA	0.88	0.58
GSE24870	GPL571	Homo Sapiens	BM cells	20	MAS 5	1.033	0.75
GSE35010	GPL6244	Homo Sapiens	BM cells	16	RMA	0.875	0.86
GSE19429	GPL570	Homo Sapiens	BM cells	17	RMA	1	0.81
GSE39540	GPL571	Homo Sapiens	BM cells	61	Quantile	1	0.74
GSE29431	GPL570	Homo Sapiens	Breast	12	RMA	1	0.86
GSE42568	GPL570	Homo Sapiens	Breast	17	gcRMA	1	0.78
GSE5364	GPL96	Homo sapiens	Breast	13	Global scaling	1	0.8

Dataset ID	Platform ID	Species	Tissue	Normal	algorithm	Cut height	MAC
GSE20437	GPL96	Homo sapiens	Breast	24	MAS 5,0	0.8	0.74
GSE21947	GPL96	Homo Sapiens	Breast	30	Mean expression	1.03	0.75
GSE10780	GPL570	Homo Sapiens	Breast	143	RMA	0.83	0.65
GSE31773	GPL570	Homo Sapiens	CD8 & CD4	17	RMA	1	0.85
GSE41328	GPL570	Homo sapiens	colon	10	PLIER	1	0.82
GSE13367	GPL570	Homo sapiens	colonic mucosa	38	RMA	1	0.77
GSE37364	GPL570	Homo sapiens	colonic mucosa	38	MAS 5,0	0.91	0.63
GSE24514	GPL96	Homo sapiens	colonic mucosa	15	Quantile	0.92	0.77
GSE22598	GPL570	Homo Sapiens	Colorectal	17	RMA	1	0.8
GSE23878	GPL570	Homo sapiens	Colorectal	24	PLIER	1	0.75
GSE32323	GPL570	Homo Sapiens	Colorectal	17	RMA	1	0.8
GSE9348	GPL570	Homo Sapiens	Colorectal	12	Global scaling	1.12	0.79
GSE15744	GPL570	Homo sapiens	Embryo	18	Global scaling	1	0.72
GSE18290	GPL570	Homo sapiens	Embryo	18	dChip signal intensity	0.81	0.79
GSE18887	GPL96	Homo sapiens	Embryo	18	RMA	1	0.79
GSE20347	GPL571	Homo Sapiens	Esophagus	17	RMA	1	0.8
GSE23400	GPL96	Homo Sapiens	Esophagus	53	RMA	0.92	0.75
GSE5364	GPL96	Homo sapiens	Esophagus	13	Global scaling	1	0.83
GSE16134	GPL570	Homo Sapiens	Gingiva	70	RMA	0.93	0.71
GSE10334	GPL570	Homo Sapiens	Gingiva	64	RMA	1	0.71
GSE22253	GPL6244	Homo Sapiens	Heart	108	gcRMA	1	0.65
GSE22459	GPL570	Homo Sapiens	Kidney	25	MAS 5	1.08	0.75
GSE9489	GPL570	Homo Sapiens	Kidney	13	Global scaling	1.08	0.78
GSE15641	GPL96	Homo Sapiens	Kidney	23	dChip	1	0.8
GSE11906	GPL570	Homo Sapiens	Large airway epithelium	21	RMA	1	0.6
GSE10135	GPL570	Homo Sapiens	Large airway epithelium	21	Global scaling	1	0.6
GSE16696	GPL570	Homo sapiens	Large airway epithelium	20	Global scaling	1	0.64
GSE18637	GPL570	Homo sapiens	Large airway epithelium	20	Global scaling	1	0.67
GSE22047	GPL570	Homo sapiens	Large airway epithelium	19	Global scaling	1.01	0.64

Dataset ID	Platform ID	Species	Tissue	Normal	algorithm	Cut height	MAC
GSE11375	GPL570	Homo Sapiens	Leukocytes	26	Global scaling	0.85	0.75
GSE38941	GPL570	Homo sapiens	Liver	10	Median normalization	0.9	0.77
GSE45267	GPL570	Homo Sapiens	Liver	41	gcRMA	1	0.73
GSE29721	GPL570	Homo Sapiens	Liver	10	RMA	1	0.85
GSE14323	GPL571	Homo Sapiens	Liver	19	RMA	1.08	0.8
GSE41258	GPL96	Homo sapiens	Liver	13	LOWSESS	0.92	0.77
GSE19804	GPL570	Homo sapiens	Lung tissue	60	RMA	1	0.68
GSE18842	GPL570	Homo sapiens	Lung tissue	45	RMA	1	0.69
GSE31552	GPL6244	Homo sapiens	Lung tissue	36	RMA	1	0.83
GSE31547	GPL96	Homo sapiens	Lung tissue	20	Quantile	1	0.74
GSE10072	GPL96	Homo Sapiens	Lung tissue	49	RMA	0.785	0.75
GSE29819	GPL570	Homo sapiens	Myocardial	12	MAS 5.0	1	0.7
GSE12485	GPL570	Homo sapiens	Myocardial	10	RMA	1.09	0.78
GSE12486	GPL570	Homo sapiens	Myocardial	10	RMA	1	0.83
GSE20950	GPL570	Homo Sapiens	Omental adipose	11	RMA	0.567	0.84
GSE41168	GPL570	Homo Sapiens	Omental adipose	35	RMA	0.965	0.66
GSE20571	GPL6244	Homo Sapiens	Omental adipose	14	Global Median	1	0.84
GSE25401	GPL6244	Homo Sapiens	Omental adipose	26	Global Median	0.8	0.71
GSE17913	GPL570	Homo Sapiens	Oral mucosa	40	RMA	0.9	0.68
GSE37265	GPL570	Homo Sapiens	Oral mucosa	19	RMA	0.85	0.76
GSE14407	GPL570	Homo Sapiens	Ovary surface epithelium	12	MAS 5	1.06	0.74
GSE38666	GPL570	Homo Sapiens	Ovary surface epithelium	12	MAS 5	1.06	0.74
GSE29220	GPL570	Homo Sapiens	Ovary surface epithelium	11	gcRMA	1.154	0.81
GSE29450	GPL570	Homo Sapiens	Ovary surface epithelium	10	RMA	1.09	0.81
GSE26712	GPL96	Homo Sapiens	Ovary surface epithelium	10	RMA	1.16	0.76
GSE16515	GPL570	Homo Sapiens	Pancreatic tissue	16	gcRMA	0.8	0.86
GSE15471	GPL570	Homo Sapiens	Pancreatic tissue	39	RMA	1	0.82
GSE28735	GPL6244	Homo sapiens	pancreatic tissue	45	RMA	1	0.79
GSE27562	GPL570	Homo sapiens	PBMC	46	RMA	0.93	0.71

Dataset ID	Platform ID	Species	Tissue	Normal	algorithm	Cut height	MAC
GSE11761	GPL570	Homo Sapiens	PBMC	40	gcRMA	1.05	0.79
GSE14642	GPL570	Homo Sapiens	PBMC	40	gcRMA	0.93	0.78
GSE15932	GPL570	Homo sapiens	PBMC	32	VSN	0.84	0.6
GSE21942	GPL570	Homo Sapiens	PBMC	15	gcRMA	0.99	0.7
GSE17114	GPL570	Homo sapiens	PBMC	14	RMA	0.875	0.73
GSE43553	GPL571	Homo Sapiens	PBMC	34	Global scaling	0.9	0.79
GSE41890	GPL6244	Homo Sapiens	PBMC	24	gcRMA	0.91	0.77
GSE28686	GPL6244	Homo Sapiens	PBMC	20	gcRMA	0.78	0.82
GSE12585	GPL96	Homo Sapiens	PBMC	23	RMA	1	0.74
GSE14577	GPL96	Homo Sapiens	PBMC	15	RMA	1	0.75
GSE25507	GPL570	Homo sapiens	Periheral lymphocytes	64	RMA	0.85	0.66
GSE7638	GPL571	Homo sapiens	Peripheral monocytes	160	NUSE	0.86	0.72
GSE36895	GPL570	Homo Sapiens	Renal cortex	23	RMA	1	0.72
GSE20950	GPL570	Homo Sapiens	Subcutaneous adipose	12	RMA	0.6	0.84
GSE26339	GPL570	Homo Sapiens	Subcutaneous adipose	11	Mas 5	1.1	0.83
GSE27916	GPL570	Homo Sapiens	Subcutaneous adipose	255	PLIER	0.93	0.72
GSE27916	GPL570	Homo Sapiens	Subcutaneous adipose	120	PLIER	0.94	0.69
GSE27949	GPL570	Homo Sapiens	Subcutaneous adipose	11	MAS5	1	0.82
GSE28005	GPL570	Homo Sapiens	Subcutaneous adipose	13	RMA	1	0.8
GSE29718	GPL6244	Homo Sapiens	Subcutaneous adipose	15	RMA	1	0.78
GSE19811	GPL96	Homo Sapiens	Subcutaneous adipose	13	RMA	1.1	0.81
GSE35710	GPL96	Homo Sapiens	Subcutaneous adipose	24	PLIER	1	0.78
GSE18206	GPL570	Homo sapiens	Skin	21	VSN	1	0.74
GSE30999	GPL570	Homo sapiens	Skin	85	gcRMA	1	0.77
GSE34248	GPL570	Homo sapiens	Skin	14	Rosetta resolver	0.92	0.76
GSE36842	GPL570	Homo sapiens	Skin	23	fRMA	0.88	0.75
GSE41662	GPL570	Homo sapiens	Skin	24	Rosetta resolver	0.8	0.69
GSE41663	GPL570	Homo sapiens	Skin	15	Rosetta resolver	0.86	0.76
GSE32407	GPL571	Homo sapiens	Skin	40	gcRMA	1.088	0.78

Dataset ID	Platform ID	Species	Tissue	Normal	algorithm	Cut height	MAC
GSE11784	GPL570	Homo sapiens	small airway epithelium	63	MAS 5,0	0.93	0.72
GSE22047	GPL570	Homo sapiens	small airway epithelium	60	Global scaling	0.93	0.62
GSE30063	GPL570	Homo sapiens	small airway epithelium	60	Global scaling	0.93	0.62
GSE19667	GPL570	Homo sapiens	small airway epithelium	48	MAS 5,0	0.84	0.58
GSE19407	GPL570	Homo Sapiens	small airway epithelium	47	MAS 5	0.86	0.72
GSE11906	GPL570	Homo Sapiens	small airway epithelium	35	RMA	1	0.74
GSE13933	GPL570	Homo sapiens	small airway epithelium	23	MAS 5,0	1.05	0.66
GSE27681	GPL570	Homo sapiens	small airway epithelium	21	Global scaling	1	0.7
GSE14924	GPL570	Homo Sapiens	T cells	21	MAS 5	1	0.62
GSE29265	GPL570	Homo Sapiens	Thyroid	20	RMA	0.71	0.71
GSE33630	GPL570	Homo Sapiens	Thyroid	46	RMA	0.69	0.69
GSE13601	GPL8300	Homo Sapiens	Tongue	27	MAS 5	0.88	0.72
GSE13070	GPL570	Homo Sapiens	Vastus lateralis	87	MAS 5.0	0.82	0.65
GSE13070	GPL570	Homo Sapiens	Vastus lateralis	53	MAS 5.0	0.85	0.67
GSE14901	GPL570	Homo Sapiens	Vastus lateralis	72	MAS 5	1.04	0.61
GSE17674	GPL570	Homo Sapiens	Vastus lateralis	18	RMA	0.925	0.8
GSE19420	GPL570	Homo sapiens	Vastus lateralis	24	MAS 5,0	1	0.63
GSE25462	GPL570	Homo sapiens	Vastus lateralis	50	MAS 5,0	1	0.6
GSE25941	GPL570	Homo Sapiens	Vastus lateralis	36	Global scaling	0.9	0.54
GSE38718	GPL570	Homo Sapiens	Vastus lateralis	22	RMA	1	0.73
GSE41168	GPL570	Homo Sapiens	Vastus lateralis	47	RMA	0.79	0.54
GSE8157	GPL570	Homo sapiens	Vastus lateralis	43	RMA	1.16	0.78
GSE9419	GPL570	Homo Sapiens	Vastus lateralis	66	MAS 5	0.803	0.52
GSE11681	GPL96	Homo sapiens	Vastus lateralis	10	RMA	1	0.78
GSE11686	GPL96	Homo Sapiens	Vastus lateralis	16	MAS5	1	0.68
GSE17371	GPL96	Homo sapiens	Vastus lateralis	12	MAS 5,0	0.9	0.72
GSE13985	GPL570	Homo sapiens	WBC	10	RMA	0.82	0.8
GSE28498	GPL6244	Homo Sapiens	WBC	26	RMA	0.6	0.82

Supplementary Table S3: Tissue Expression Profiles of ExtRAAS Genes

Supplementary Table S3: Tissue expression profiles of extRAAS genes. Tissues are arranged according to the tissue dendrogram of figure 5 in the article (see dendrogram below). PBMC, peripheral blood mononuclear cells; small airways, small airways epithelium; large airways, large airways epithelium; SC adipose, sub-cutaneous adipose tissue.

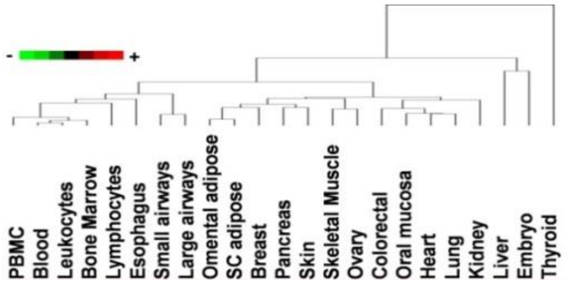

Gene symbol	PBMC	Blood	Leukocytes	Bone Marrow	Lymphocytes	Esophagus	Small airways	Large airways
**ACE**	31 ± 5	35 ± 4	36 ± 7	43 ± 6	37 ± 7	14 ± 3	50 ± 1	55 ± 0
**ACE2**	20 ± 4	24 ± 4	19 ± 5	23 ± 5	40 ± 4	36 ± 11	54 ± 0	59 ± 0
**AGT**	41 ± 3	47 ± 4	42 ± 9	39 ± 5	49 ± 4	43 ± 5	47 ± 0	51 ± 0
**AGTR1**	24 ± 4	28 ± 5	25 ± 6	31 ± 4	42 ± 5	32 ± 13	33 ± 1	36 ± 1
**AGTR2**	30 ± 4	26 ± 4	21 ± 7	20 ± 4	37 ± 3	21 ± 3	26 ± 1	35 ± 1
**AKR1C4**	16 ± 4	17 ± 3	10 ± 2	11 ± 2	23 ± 5	13 ± 5	38 ± 1	44 ± 0
**AKR1D1**	13 ± 4	11 ± 3	6 ± 3	6 ± 3	18 ± 5	8 ± 6	16 ± 1	15 ± 0
**ANPEP**	86 ± 4	94 ± 1	96 ± 2	71 ± 7	57 ± 10	21 ± 6	84 ± 0	75 ± 0
**ATP6AP2**	98 ± 1	97 ± 1	98 ± 1	94 ± 3	99 ± 1	95 ± 2	99 ± 0	99 ± 0
**CMA1**	18 ± 2	23 ± 2	22 ± 3	22 ± 3	26 ± 3	26 ± 7	21 ± 1	13 ± 1
**CPA3**	58 ± 5	67 ± 3	46 ± 12	76 ± 11	62 ± 5	72 ± 6	89 ± 0	82 ± 0
**CTSA**	98 ± 1	96 ± 1	98 ± 1	87 ± 4	90 ± 4	91 ± 3	93 ± 0	92 ± 0
**CTSD**	85 ± 3	82 ± 2	91 ± 4	71 ± 4	70 ± 6	78 ± 3	93 ± 0	93 ± 0
**CTSG**	48 ± 3	53 ± 3	56 ± 10	87 ± 7	62 ± 4	57 ± 5	35 ± 1	30 ± 0
**CYP11A1**	20 ± 3	29 ± 2	23 ± 4	25 ± 3	36 ± 6	39 ± 5	21 ± 2	11 ± 1
**CYP11B1**	25 ± 6	32 ± 6	26 ± 13	26 ± 7	34 ± 7	22 ± 10	15 ± 1	14 ± 1
**CYP11B2**	47 ± 6	45 ± 5	58 ± 10	47 ± 6	29 ± 13	39 ± 9	21 ± 2	27 ± 0
**CYP17A1**	27 ± 4	36 ± 4	36 ± 7	33 ± 6	43 ± 2	40 ± 8	33 ± 1	32 ± 1
**CYP21A2**	34 ± 5	37 ± 4	42 ± 5	37 ± 4	31 ± 9	30 ± 9	24 ± 1	21 ± 1
**DPP3**	78 ± 3	74 ± 4	75 ± 3	72 ± 5	71 ± 9	56 ± 8	79 ± 1	82 ± 0
**EGFR**	28 ± 7	43 ± 5	43 ± 7	51 ± 8	49 ± 9	62 ± 16	35 ± 1	33 ± 0
**ENPEP**	15 ± 5	7 ± 3	8 ± 3	11 ± 3	3 ± 1	11 ± 6	4 ± 1	18 ± 1
**GPER**	41 ± 6	49 ± 4	56 ± 1	40 ± 7	34 ± 8	34 ± 7	21 ± 1	17 ± 0
**HSD11B1**	34 ± 3	44 ± 3	34 ± 6	29 ± 5	50 ± 2	34 ± 8	41 ± 1	38 ± 1
**HSD11B2**	23 ± 5	23 ± 4	31 ± 7	31 ± 7	16 ± 6	38 ± 3	32 ± 1	35 ± 1
**IGF2R**	93 ± 2	96 ± 1	95 ± 3	72 ± 6	91 ± 5	84 ± 1	89 ± 0	89 ± 0
**KLK1**	62 ± 3	64 ± 3	64 ± 6	58 ± 6	65 ± 3	57 ± 2	55 ± 1	47 ± 0
**LNPEP**	66 ± 8	48 ± 5	53 ± 15	55 ± 10	56 ± 7	28 ± 2	49 ± 0	54 ± 0
**MAS1**	21 ± 5	27 ± 4	19 ± 5	23 ± 5	35 ± 3	48 ± 8	22 ± 1	19 ± 1
**MME**	66 ± 8	95 ± 3	88 ± 10	72 ± 9	43 ± 16	42 ± 14	45 ± 1	36 ± 1
**NR3C1**	94 ± 2	94 ± 2	95 ± 2	89 ± 3	95 ± 1	82 ± 6	93 ± 0	93 ± 0
**NR3C2**	71 ± 3	57 ± 4	50 ± 13	32 ± 6	80 ± 5	30 ± 10	85 ± 0	85 ± 0
**PREP**	79 ± 2	79 ± 2	78 ± 2	74 ± 4	79 ± 5	75 ± 5	77 ± 0	79 ± 0
**REN**	30 ± 5	33 ± 4	30 ± 5	36 ± 5	30 ± 4	33 ± 12	34 ± 1	25 ± 1
**RNPEP**	91 ± 1	91 ± 2	94 ± 2	82 ± 5	87 ± 2	92 ± 1	96 ± 0	96 ± 0
**THOP1**	55 ± 3	51 ± 4	55 ± 5	59 ± 7	58 ± 5	48 ± 7	68 ± 0	68 ± 0

Gene symbol	Omental adipose	SC adipose	Breast	Pancreas	Skin	Skeletal Muscle	Ovary	Colorectal
**ACE**	67 ± 1	68 ± 2	38 ± 12	58 ± 6	44 ± 5	50 ± 4	23 ± 5	61 ± 7
**ACE2**	38 ± 1	35 ± 1	44 ± 3	56 ± 1	28 ± 4	36 ± 4	37 ± 8	84 ± 4
**AGT**	51 ± 5	63 ± 1	58 ± 7	70 ± 8	60 ± 3	89 ± 1	60 ± 4	66 ± 4
**AGTR1**	93 ± 1	94 ± 1	82 ± 5	63 ± 4	84 ± 5	80 ± 2	76 ± 5	51 ± 6
**AGTR2**	18 ± 3	27 ± 1	31 ± 3	25 ± 5	20 ± 4	37 ± 3	36 ± 2	34 ± 3
**AKR1C4**	49 ± 12	25 ± 3	22 ± 5	28 ± 8	18 ± 3	19 ± 2	20 ± 6	18 ± 4
**AKR1D1**	9 ± 2	2 ± 0	23 ± 9	15 ± 6	14 ± 4	15 ± 3	13 ± 4	9 ± 4
**ANPEP**	67 ± 2	70 ± 2	79 ± 3	99 ± 1	73 ± 6	33 ± 2	31 ± 5	96 ± 1
**ATP6AP2**	96 ± 1	98 ± 0	98 ± 1	98 ± 1	98 ± 1	88 ± 1	100 ± 0	98 ± 2
**CMA1**	51 ± 1	45 ± 2	45 ± 6	33 ± 4	60 ± 7	36 ± 2	28 ± 5	41 ± 5
**CPA3**	75 ± 3	76 ± 1	65 ± 9	94 ± 2	78 ± 6	57 ± 2	34 ± 12	80 ± 5
**CTSA**	95 ± 0	95 ± 0	85 ± 4	95 ± 2	94 ± 1	85 ± 2	79 ± 3	99 ± 1
**CTSD**	89 ± 2	87 ± 1	74 ± 6	90 ± 5	83 ± 3	78 ± 2	53 ± 9	91 ± 2
**CTSG**	65 ± 1	75 ± 1	57 ± 9	56 ± 7	70 ± 5	69 ± 2	52 ± 1	67 ± 3
**CYP11A1**	37 ± 2	37 ± 1	38 ± 3	21 ± 10	20 ± 6	33 ± 2	37 ± 7	27 ± 4
**CYP11B1**	33 ± 7	26 ± 2	31 ± 9	22 ± 11	14 ± 1	31 ± 4	30 ± 6	22 ± 7
**CYP11B2**	46 ± 4	64 ± 1	37 ± 9	44 ± 15	16 ± 6	46 ± 6	28 ± 7	48 ± 7
**CYP17A1**	36 ± 1	39 ± 1	31 ± 7	25 ± 12	14 ± 5	49 ± 3	33 ± 8	38 ± 4
**CYP21A2**	44 ± 3	49 ± 1	38 ± 9	30 ± 11	74 ± 11	39 ± 4	50 ± 7	34 ± 7
**DPP3**	72 ± 2	81 ± 1	66 ± 9	74 ± 8	72 ± 3	59 ± 3	70 ± 3	92 ± 2
**EGFR**	67 ± 5	58 ± 3	75 ± 3	54 ± 21	44 ± 4	61 ± 4	86 ± 5	55 ± 9
**ENPEP**	88 ± 1	66 ± 3	56 ± 8	72 ± 3	46 ± 6	57 ± 4	43 ± 9	37 ± 9
**GPER**	75 ± 2	83 ± 1	54 ± 12	44 ± 5	54 ± 5	48 ± 3	57 ± 4	63 ± 5
**HSD11B1**	77 ± 5	91 ± 0	64 ± 8	61 ± 7	82 ± 2	61 ± 2	78 ± 2	46 ± 2
**HSD11B2**	37 ± 0	31 ± 1	34 ± 4	45 ± 1	73 ± 4	34 ± 1	37 ± 5	98 ± 1
**IGF2R**	93 ± 1	91 ± 1	81 ± 5	92 ± 2	87 ± 2	93 ± 1	77 ± 4	92 ± 3
**KLK1**	57 ± 2	61 ± 1	58 ± 4	98 ± 1	83 ± 3	67 ± 2	69 ± 2	91 ± 2
**LNPEP**	72 ± 7	29 ± 2	31 ± 9	59 ± 17	44 ± 4	46 ± 4	30 ± 7	43 ± 5
**MAS1**	20 ± 3	37 ± 1	35 ± 7	16 ± 10	18 ± 4	45 ± 3	35 ± 8	45 ± 2
**MME**	96 ± 1	95 ± 0	91 ± 3	62 ± 8	62 ± 4	79 ± 2	43 ± 11	50 ± 7
**NR3C1**	97 ± 1	97 ± 1	94 ± 3	93 ± 2	96 ± 1	98 ± 0	97 ± 2	82 ± 7
**NR3C2**	73 ± 3	71 ± 1	78 ± 3	83 ± 3	75 ± 2	87 ± 1	83 ± 8	95 ± 2
**PREP**	74 ± 3	75 ± 1	74 ± 5	80 ± 3	79 ± 2	94 ± 0	76 ± 1	92 ± 2
**REN**	24 ± 1	27 ± 1	25 ± 8	24 ± 10	24 ± 6	27 ± 3	29 ± 6	38 ± 4
**RNPEP**	86 ± 1	85 ± 1	87 ± 2	93 ± 2	81 ± 3	83 ± 1	78 ± 5	96 ± 1
**THOP1**	62 ± 2	53 ± 2	52 ± 5	59 ± 3	44 ± 8	64 ± 3	47 ± 4	75 ± 3

Gene symbol	Oral Mucosa	Heart	Lung	Kidney	Liver	Embryo	Thyroid
**ACE**	53 ± 3	57 ± 5	65 ± 11	56 ± 12	35 ± 11	32 ± 9	58 ± 5
**ACE2**	50 ± 4	79 ± 2	36 ± 4	92 ± 3	48 ± 8	44 ± 15	40 ± 7
**AGT**	51 ± 2	94 ± 4	51 ± 4	90 ± 4	100 ± 1	84 ± 4	66 ± 2
**AGTR1**	38 ± 6	63 ± 4	71 ± 4	82 ± 8	98 ± 1	53 ± 7	98 ± 1
**AGTR2**	24 ± 4	36 ± 8	60 ± 6	39 ± 5	28 ± 3	49 ± 5	50 ± 7
**AKR1C4**	7 ± 2	11 ± 3	10 ± 2	19 ± 6	96 ± 2	23 ± 5	36 ± 4
**AKR1D1**	3 ± 0	8 ± 3	5 ± 3	11 ± 6	97 ± 2	33 ± 8	91 ± 0
**ANPEP**	69 ± 3	48 ± 4	65 ± 11	98 ± 1	98 ± 1	48 ± 9	58 ± 4
**ATP6AP2**	99 ± 0	95 ± 1	97 ± 1	99 ± 1	96 ± 2	95 ± 1	82 ± 5
**CMA1**	44 ± 4	41 ± 5	28 ± 5	26 ± 2	20 ± 3	16 ± 5	63 ± 4
**CPA3**	84 ± 7	59 ± 8	96 ± 1	60 ± 1	49 ± 6	18 ± 5	60 ± 12
**CTSA**	95 ± 1	94 ± 0	96 ± 1	99 ± 0	93 ± 2	78 ± 2	77 ± 11
**CTSD**	83 ± 1	88 ± 4	96 ± 1	92 ± 1	91 ± 2	60 ± 2	89 ± 3
**CTSG**	72 ± 2	64 ± 9	59 ± 6	59 ± 2	53 ± 11	43 ± 4	83 ± 2
**CYP11A1**	55 ± 2	32 ± 3	31 ± 5	21 ± 4	45 ± 8	39 ± 6	26 ± 6
**CYP11B1**	7 ± 1	20 ± 9	22 ± 7	27 ± 9	20 ± 12	17 ± 4	65 ± 0
**CYP11B2**	57 ± 4	57 ± 10	48 ± 4	34 ± 13	54 ± 7	39 ± 6	41 ± 12
**CYP17A1**	37 ± 3	47 ± 9	36 ± 4	85 ± 4	53 ± 6	41 ± 1	79 ± 7
**CYP21A2**	45 ± 4	47 ± 4	40 ± 4	32 ± 8	61 ± 9	33 ± 5	6 ± 0
**DPP3**	86 ± 2	74 ± 3	75 ± 5	77 ± 5	74 ± 5	66 ± 5	4 ± 1
**EGFR**	69 ± 3	54 ± 9	63 ± 7	70 ± 11	68 ± 7	47 ± 9	45 ± 6
**ENPEP**	43 ± 9	69 ± 4	49 ± 9	97 ± 1	93 ± 2	30 ± 8	92 ± 4
**GPER**	57 ± 2	56 ± 2	61 ± 4	63 ± 9	73 ± 9	35 ± 5	56 ± 3
**HSD11B1**	44 ± 6	59 ± 10	70 ± 7	54 ± 9	99 ± 1	36 ± 6	56 ± 6
**HSD11B2**	49 ± 3	35 ± 7	40 ± 3	98 ± 0	36 ± 5	36 ± 2	83 ± 2
**IGF2R**	94 ± 1	93 ± 1	90 ± 2	92 ± 4	94 ± 1	83 ± 3	100 ± 0
**KLK1**	66 ± 3	68 ± 6	55 ± 5	84 ± 2	56 ± 7	54 ± 5	30 ± 3
**LNPEP**	40 ± 3	72 ± 8	47 ± 13	31 ± 7	29 ± 8	44 ± 8	49 ± 9
**MAS1**	31 ± 2	32 ± 8	33 ± 9	38 ± 4	42 ± 3	37 ± 6	25 ± 2
**MME**	66 ± 3	64 ± 7	89 ± 2	97 ± 1	71 ± 7	68 ± 3	93 ± 1
**NR3C1**	94 ± 1	92 ± 4	91 ± 2	90 ± 5	92 ± 3	45 ± 13	63 ± 2
**NR3C2**	48 ± 7	86 ± 3	73 ± 5	88 ± 3	69 ± 9	42 ± 12	36 ± 3
**PREP**	86 ± 1	82 ± 2	77 ± 3	74 ± 5	77 ± 7	88 ± 1	29 ± 8
**REN**	48 ± 4	33 ± 7	44 ± 4	79 ± 7	37 ± 3	28 ± 7	41 ± 8
**RNPEP**	95 ± 1	85 ± 1	91 ± 2	86 ± 5	87 ± 2	87 ± 0	48 ± 4
**THOP1**	71 ± 2	67 ± 3	51 ± 9	48 ± 11	69 ± 7	67 ± 2	12 ± 1

Supplementary Table S4: ExtRAAS Co-expression Modules in All 23 Studied Tissues

Supplementary Table S4: ExtRAAS co-expression modules in all 23 studied tissue. Below each tissue the number of datasets and corresponding samples are represented as (datasets, samples). At the top of each module the average coordination rate is expressed in percentage (average percentage of genes within a module that are always coordinated across the different datasets of a specific tissue). Next to each gene symbol the abundance of the mRNA is expressed in mean MCR (Mean expression centile rank). Genes corresponding to core groups are colored; CTSA and CTSD in red; MR, GR and AGTR1 in blue; and CTSG, CPA3 and CMA1in green. Tissues are arranged based on the tissue dendrogram of figure 5 in the article (see dendrogram below).

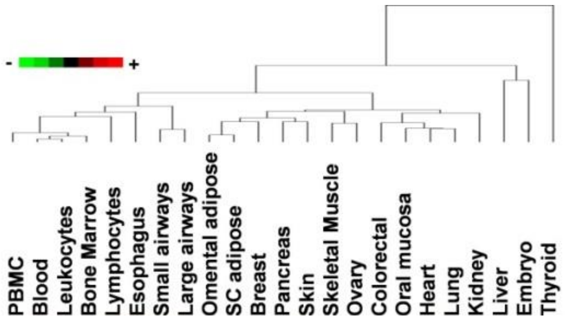

Tissues (datasets, samples)	Module 1		Module 2		Module 3		Module 4		Module 5		Module 6		Module 7		Module 8		Non-clustered genes	
**Thyroid (2, 66)**	100%		100%		100%		100%		100%		100%		100%		100%			
	CTSD	89	ATP6AP2	82	MME	93	CYP11B2	41	AGTR1	98	AKR1D1	91	PREP	29	LNPEP	49	IGF2R	100
	CYP17A1	79	GR	63	CTSG	83	KLK1	30	ENPEP	92	CYP11B1	65	THOP1	12	REN	41	CTSA	77
	RNPEP	48	GPER	56	CMA1	63			HSD11B2	83	AKR1C4	36					AGT	66
			ACE2	40	CPA3	60			ACE	58							ANPEP	58
					HSD11B1	56											AGTR2	50
																	EGFR	45
																	MR	36
																	CYP11A1	26
																	MAS1	25
																	CYP21A2	6
																	DPP3	4
**Embryo (4, 66)**	83%		80%		83%		87%		87%		80%							
	ATP6AP2	95	AGTR2	49	CTSG	43	ANPEP	48	PREP	88	AGT	84					IGF2R	83
	RNPEP	87	LNPEP	44	CYP17A1	41	GR	45	KLK1	54	AGTR1	53					CTSA	78
	THOP1	67	MR	42	MAS1	37	CYP11A1	39	CYP11B2	39	AKR1D1	33					MME	68
	DPP3	66	GPER	35	CYP11B1	17	ENPEP	30	HSD11B1	36	AKR1C4	23					CTSD	60
			CYP21A2	33			REN	28	ACE	32	CPA3	18					EGFR	47
																	ACE2	44
																	HSD11B2	36
																	CMA1	16
**Liver (5, 93)**	83%		60%		87%		80%		80%		74%		74%		67%			
	ATP6AP2	96	AGTR1	98	HSD11B1	99	EGFR	68	ANPEP	98	MAS1	42	ENPEP	93	CYP11B2	54	AGT	100
	IGF2R	94	GR	92	AKR1D1	97	REN	37	KLK1	56	LNPEP	29	MME	71	CYP17A1	53	RNPEP	87
	CTSA	93			AKR1C4	96	CYP11B1	20	HSD11B2	36	AGTR2	28	MR	69	CYP11A1	45	PREP	77
	CTSD	91															GPER	73
	DPP3	74															THOP1	69
	CYP21A2	61															CTSG	53
	ACE	35															CPA3	49
																	ACE2	48
																	CMA1	20
**Kidney (4, 84)**	84%		88%		85%		94%		80%									
	CTSA	99	ATP6AP2	99	CTSG	59	THOP1	48	PREP	74							HSD11B2	98
	ANPEP	98	GR	90	AGTR2	39	CYP11B2	34	CPA3	60							IGF2R	92
	ENPEP	97	MR	88	MAS1	38	CYP21A2	32	HSD11B1	54							RNPEP	86
	MME	97	AGTR1	82	AKR1C4	19	CMA1	26	LNPEP	31							DPP3	77
	ACE2	92			AKR1D1	11			CYP11A1	21							EGFR	70
	CTSD	92															GPER	63
	AGT	90															CYP11B1	27
	CYP17A1	85																
	KLK1	84																
	REN	75																
	ACE	56																

**Lung**	90%		85%		100%		80%		86%		93%		80%		100%			
**(5, 210)**	CTSD	96	CPA3	96	CTSG	59	KLK1	55	EGFR	63	ATP6AP2	97	CYP11B2	48	AKR1C4	10	ACE	65
	CTSA	96	GR	91	CMA1	28	HSD11B2	40	REN	44	IGF2R	90	CYP21A2	40	AKR1D1	5	GPER	61
	RNPEP	91	MME	89			CYP11A1	31	MAS1	33	PREP	77					AGTR2	60
	DPP3	75	MR	73			CYP11B1	22									AGT	51
	ANPEP	65	AGTR1	71													THOP1	51
	LNPEP	47	HSD11B1	70													ACE2	36
			ENPEP	49													CYP17A1	36
**Heart**	77%		75%		80%		100%		81%		81%		75%					
**(4, 140)**	CTSA	94	GR	92	CTSG	64	EGFR	54	KLK1	68	IGF2R	93	ACE2	79			ATP6AP2	95
	AGT	94	ENPEP	69	AGTR1	63	REN	33	CMA1	41	MME	64	LNPEP	72			MR	86
	CTSD	88			CPA3	59	MAS1	32	HSD11B2	35	HSD11B1	59					RNPEP	85
	DPP3	74			ACE	57			CYP11B1	20	AKR1D1	8					PREP	82
	THOP1	67			AKR1C4	11											CYP11B2	57
																	GPER	56
																	ANPEP	48
																	CYP21A2	47
																	CYP17A1	47
																	AGTR2	36
																	CYP11A1	32
**Oral mucosa**	81%		71%		96%		75%		87%		75%		75%		83%			
**(4, 193)**	CTSA	95	GR	94	CPA3	84	REN	48	RNPEP	95	KLK1	66	ACE2	50	AGT	69	ATP6AP2	99
	DPP3	86	MR	48	CTSG	72	MAS1	31	IGF2R	94	HSD11B2	49	LNPEP	40	AKR1C4	51	CYP11B2	57
	THOP1	71			ANPEP	69	AGTR2	24	PREP	86					EGFR	7	GPER	57
	CYP11A1	55			MME	66	CYP11B1	7	CTSD	83							ACE	53
					HSD11B1	44	AKR1D1	3									CYP21A2	45
					CMA1	44											AGTR1	38
					ENPEP	43											CYP17A1	37
**Colorectal**	82%		78%		96%		80%		83%		75%		75%		62%			
**(8, 171)**	CTSA	98	ATP6AP2	98	CPA3	80	RNPEP	96	ANPEP	96	AGT	66	EGFR	55	ACE	61	LNPEP	43
	HSD11B2	98	GR	82	CTSG	66	PREP	92	ACE2	84	CYP11B2	48	CYP17A1	37	MME	50	REN	37
	MR	95	GPER	63	CMA1	41	DPP3	91	MAS1	44	AGTR2	34					ENPEP	36
	IGF2R	91	AGTR1	50			KLK1	91	AKR1C4	18	CYP21A2	34					CYP11A1	26
	CTSD	91	HSD11B1	45			THOP1	74	AKR1D1	9	CYP11B1	21						
**Ovary Epithelium**	85%		80%		80%		88%		90%		84%		85%		85%			
**(5, 55)**	CTSA	79	GR	97	EGFR	86	CTSG	52	THOP1	47	ATP6AP2	100	KLK1	69	AGT	60	RNPEP	78
	IGF2R	77	MR	83	CYP17A1	33	ACE2	37	MME	43	HSD11B1	78	CYP21A2	50	CTSD	53	PREP	76
	DPP3	70	CYP11B1	30	REN	29	MAS1	35	HSD11B2	37	AGTR1	76	CYP11B2	28	AGTR2	36	LNPEP	30
	GPER	57			CMA1	28	AKR1C4	20	CYP11A1	37	ENPEP	43	ACE	23	ANPEP	31		
							AKR1D1	13			CPA3	34						
**Skeletal Muscle**	64%		76%		57%		74%		67%		57%							
**(14, 556)**	CTSA	85	GR	98	CPA3	57	KLK1	67	CYP11B2	46	ATP6AP2	88					PREP	94
	RNPEP	83	IGF2R	93	CMA1	36	CYP17A1	49	CYP21A2	39	AGTR1	80					AGT	89
	CTSD	78	MR	87			REN	27	AKR1C4	19							MME	79
																	CTSG	69
																	THOP1	64
																	HSD11B1	61
																	EGFR	61
																	DPP3	59
																	ENPEP	57

																	ACE	50
																	GPER	48
																	LNPEP	46
																	MAS1	45
																	AGTR2	37
																	ACE2	36
																	HSD11B2	34
																	CYP11A1	33
																	ANPEP	33
																	CYP11B1	31
																	AKR1D1	15
**Skin (7, 222)**	81%		57%		90%		71%		57%		71%							
	GPER	54	AGTR1	84	CPA3	78	THOP1	44	GR	96	ATP6AP2	98					CTSA	94
	ACE	44	MR	75	CTSG	70	REN	24	HSD11B2	73	ACE2	28					IGF2R	87
	CYP11B2	16			CMA1	60											CTSD	83
																	KLK1	83
																	HSD11B1	82
																	RNPEP	81
																	PREP	79
																	CYP21A2	74
																	ANPEP	73
																	DPP3	72
																	MME	62
																	AGT	60
																	ENPEP	46
																	EGFR	44
																	LNPEP	44
																	CYP11A1	20
																	AGTR2	20
																	MAS1	18
																	AKR1C4	18
																	AKR1D1	14
																	CYP11B1	14
																	CYP17A1	14
**Pancreas (3, 100)**	100%		100%		100%		100%		100%		100%							
	ATP6AP2	98	DPP3	74	CPA3	94	RNPEP	93	CYP11B2	44	ANPEP	99					MR	83
	CTSA	95	THOP1	59	CTSG	56	PREP	80	CYP21A2	30	KLK1	98					AGT	70
	GR	93			CMA1	33	ACE	58	AGTR2	25	MAS1	16					AGTR1	63
	IGF2R	92					ACE2	56	CYP17A1	25							MME	62
	CTSD	90					HSD11B2	45	REN	24							EGFR	54
	ENPEP	72							CYP11B1	22							GPER	44
	HSD11B1	61							AKR1D1	15							AKR1C4	28
	LNPEP	59															CYP11A1	21
**Sub-cutaneous adipose (9, 474)**	81%		88%		96%		78%		85%		75%		78%		79%			
	CTSA	95	GR	97	CPA3	76	ATP6AP2	98	CYP11B2	64	ACE	68	CYP17A1	39	MME	95	HSD11B1	91
	IGF2R	91	AGTR1	94	CTSG	75	GPER	83	KLK1	61	THOP1	53	MAS1	37	AGT	63	EGFR	58
	CTSD	87	MR	71	CMA1	45	DPP3	81	CYP21A2	49	AKR1C4	25	REN	27	LNPEP	29	ACE2	35
	RNPEP	85	ENPEP	66			ANPEP	70	HSD11B2	31			CYP11B1	26				
	PREP	75							AGTR2	27								
	CYP11A1	37							AKR1D1	2								

**Omental adipose (4, 86)**	91%		83%		83%		85%		75%									
	ATP6AP2	96	GR	97	ACE	67	AGT	51	PREP	74								
	CTSA	95	MME	96	KLK1	57	ACE2	38	LNPEP	72								
	CTSD	89	AGTR1	93	CYP11B2	46	REN	24										
	RNPEP	86	IGF2R	93	CYP21A2	44	MAS1	20										
	HSD11B1	77	ENPEP	88	CYP11A1	37	AKR1D1	9										
	CPA3	75	GPER	75	HSD11B2	37												
	DPP3	72	MR	73	CYP17A1	36												
	CTSG	65	ANPEP	67	CYP11B1	33												
	THOP1	62	EGFR	67	AGTR2	18												
	CMA1	51																
	AKR1C4	49																
**Breast (6, 239)**	86%		83%		73%		84%		83%		75%		83%					
	RNPEP	86	ATP6AP2	98	CPA3	65	EGFR	75	ACE2	43	AGT	57	ANPEP	79			MME	90
	CTSA	85	GR	94	CTSG	57	MAS1	35	REN	25	ACE	38	HSD11B2	34			HSD11B1	63
	CTSD	74	AGTR1	82	CMA1	45	AGTR2	31			CYP11A1	38					KLK1	57
	PREP	73	IGF2R	81							CYP11B1	31					GPER	54
	DPP3	66	MR	78													CYP21A2	38
	THOP1	52	ENPEP	55													CYP11B2	36
																	CYP17A1	31
																	LNPEP	30
																	AKR1D1	23
																	AKR1C4	22
**Large airways (5, 101)**	96%		93%		100%		96%		95%		93%		73%		80%			
	RNPEP	96	ATP6AP2	99	CPA3	82	ANPEP	75	EGFR	33	HSD11B1	38	IGF2R	89	CYP17A1	32	AGT	51
	CTSD	93	GR	93	AKR1C4	44	ACE	55	REN	25	HSD11B2	35	DPP3	82	CMA1	13	KLK1	47
	CTSA	92	MR	85	CTSG	30	MME	36	MAS1	19	CYP21A2	21	AKR1D1	15			CYP11B1	14
	PREP	79	ACE2	59	CYP11A1	11	AGTR1	36	ENPEP	18								
	THOP1	68	LNPEP	54			CYP11B2	27										
			AGTR2	35			GPER	17										
**Small airways (8, 357)**	96%		100%		90%		88%		78%		91%		88%					
	RNPEP	96	GR	93	CPA3	89	REN	34	MME	45	ATP6AP2	99	CYP21A2	24			KLK1	55
	CTSD	93	MR	85	EGFR	35	AGTR1	33	AGTR2	26	AGT	47	CYP11B2	21			ACE	50
	CTSA	93			CTSG	35	CYP17A1	33	AKR1D1	16	HSD11B1	41					LNPEP	49
	IGF2R	89			HSD11B2	32	CYP11A1	21	ENPEP	4	AKR1C4	38					MAS1	22
	ANPEP	84					CMA1	21									GPER	21
	DPP3	79					CYP11B1	15										
	PREP	77																
	THOP1	68																
	ACE2	54																
**Esophagus (3, 83)**	92%		92%		92%		100%		92%		92%							
	RNPEP	91	GR	82	CPA3	72	AGT	42	CYP21A2	30	ATP6AP2	95					EGFR	62
	CTSA	90	AGTR1	31	CTSG	57	GPER	34	ACE	14	IGF2R	83					KLK1	56
	CTSD	78	MR	30	CMA1	26	HSD11B1	33	AKR1C4	13	LNPEP	28					MAS1	48
	PREP	74	ENPEP	10													MME	42
	DPP3	55															CYP17A1	40
	THOP1	48															CYP11B2	39
	CYP11A1	39															HSD11B2	38
	ACE2	36															REN	33
																	CYP11B1	22
																	ANPEP	21

																	AGTR2	20
																	AKR1D1	8
**Lymphocytes**	77%		95%		83%		93%		83%		79%		75%					
**(6, 184)**	IGF2R	90	ATP6AP2	99	CTSG	61	EGFR	49	CYP17A1	43	KLK1	65	CTSD	70			HSD11B1	50
	CTSA	90	GR	95	ANPEP	56	AGT	48	ACE	37	CPA3	61	THOP1	58			HSD11B2	16
	RNPEP	87	MR	90	MME	42	REN	30	AGTR2	37	LNPEP	56	GPER	33				
	PREP	79			AGTR1	42	AKR1C4	23	CYP11A1	35	ACE2	40	CYP21A2	31				
	DPP3	70			MAS1	34	AKR1D1	18										
	CYP11B1	34			CMA1	26												
	CYP11B2	28																
	ENPEP	3																
**Bone marrow**	72%		75%		71%		73%		78%		75%		72%		67%			
**(8, 208)**	CTSA	86	GR	89	CTSG	87	CYP21A2	36	EGFR	51	CYP17A1	33	ACE	43	KLK1	57	ATP6AP2	94
	RNPEP	82	ANPEP	70	CPA3	76	MR	32	GPER	40	AGTR1	31	AGT	39	CYP11B2	47	MME	72
	PREP	74	LNPEP	55	DPP3	72	CYP11B1	26	HSD11B2	31	MAS1	22	REN	35	HSD11B1	29	THOP1	59
	IGF2R	71					CYP11A1	25	ENPEP	10	AKR1D1	6	ACE2	23				
	CTSD	70					AGTR2	19					CMA1	22				
							AKR1C4	10										
**Leukocytes**	83%		75%		75%		88%		79%		75%		75%		75%			
**(4, 222)**	ATP6AP2	98	MR	50	CTSG	56	CYP17A1	36	KLK1	64	AGT	42	CYP11B1	26	AGTR2	21	ANPEP	96
	CTSA	97	CPA3	46	ENPEP	8	ACE	35	CYP11B2	58	AGTR1	24	AKR1D1	6	AKR1C4	9	IGF2R	95
	RNPEP	93					HSD11B2	31	GPER	55							GR	95
	CTSD	91					REN	30	CYP21A2	41							MME	88
	PREP	78					CMA1	22	HSD11B1	34							THOP1	55
	DPP3	75					ACE2	19	CYP11A1	23							LNPEP	53
																	EGFR	43
																	MAS1	19
**Total Blood**	76%		69%		65%		71%		69%									
**(17, 774)**	IGF2R	96	ATP6AP2	97	PREP	79	HSD11B1	44	REN	33							CPA3	67
	CTSA	96	GR	94	DPP3	74	CYP17A1	36	CYP11B1	32							KLK1	64
	MME	95	MR	57					AKR1D1	11							CTSG	53
	ANPEP	94	LNPEP	48													THOP1	51
	RNPEP	91															GPER	49
	CTSD	82															AGT	47
																	CYP11B2	45
																	EGFR	43
																	CYP21A2	37
																	ACE	35
																	CYP11A1	29
																	AGTR1	28
																	MAS1	27
																	AGTR2	26
																	ACE2	24
																	CMA1	23
																	HSD11B2	23
																	AKR1C4	17
																	ENPEP	7

**PBMC**	71%		77%		70%		68%		79%		79%							
**(11, 303)**	CTSA	97	ATP6AP2	98	AGTR2	29	AGT	40	KLK1	61	CYP17A1	26					ANPEP	86
	IGF2R	93	GR	94	AGTR1	23	ACE	31	CYP11B2	47	CYP11A1	20					PREP	79
	RNPEP	91	MR	71	AKR1D1	13	REN	30	GPER	40	ENPEP	15					DPP3	78
	CTSD	84	LNPEP	65			MAS1	21									MME	66
	THOP1	54															CPA3	58
																	CTSG	48
																	CYP21A2	34
																	HSD11B1	34
																	EGFR	28
																	CYP11B1	25
																	HSD11B2	23
																	ACE2	20
																	CMA1	18
																	AKR1C4	16

Supplementary Figure S1: ExtRAAS Gene Expression Profiles Across Studied Tissues

Supplementary Figure S1: ExtRAAS gene expression profiles across studied tissues. The expression profile of each of the extRAAS genes across all studied tissues represented in a bar-graph as mean expression centile rank (MCR) ± SEM. A horizontal cut-off line was drawn at the MCR level 33.3.

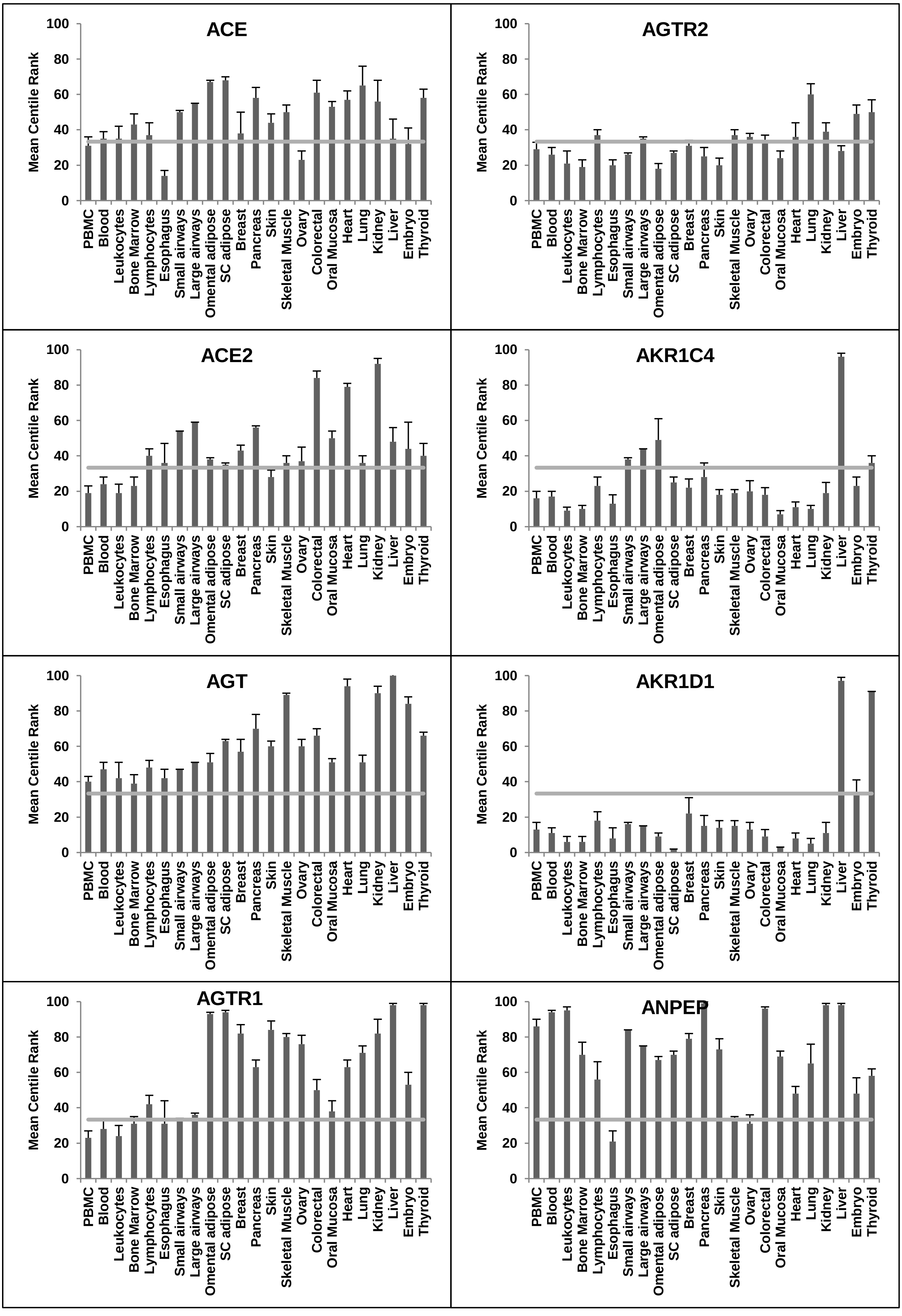
ACE
AGTR2
ACE2
AKR1C4
AGT
AKR1D1
AGTR1
ANPEP
Mean Centile Rank
0
20
40
60
80
100
PBMC
Blood
Leukocytes
Bone Marrow
Lymphocytes
Esophagus
Small airways
Large airways
Omental adipose
SC adipose
Breast
Pancreas
Skin
Skeletal Muscle
Ovary
Colorectal
Oral Mucosa
Heart
Lung
Kidney
Liver
Embryo
Thyroid

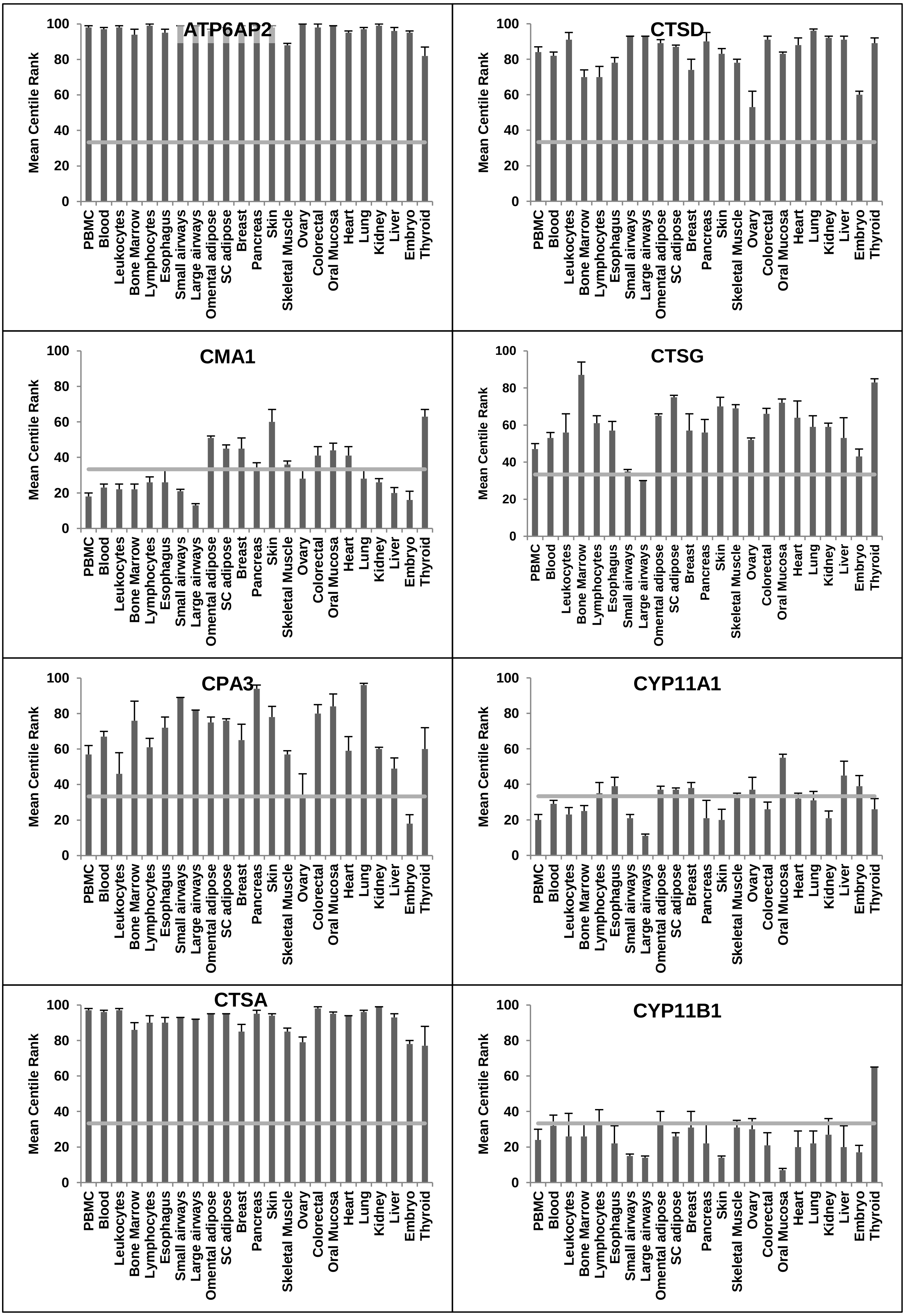
ATP6AP2
CTSD
CMA1
CTSG
CPA3
CYP11A1
CTSA
CYP11B1
Mean Centile Rank
0
20
40
60
80
100
PBMC
Blood
Leukocytes
Bone Marrow
Lymphocytes
Esophagus
Small airways
Large airways
Omental adipose
SC adipose
Breast
Pancreas
Skin
Skeletal Muscle
Ovary
Colorectal
Oral Mucosa
Heart
Lung
Kidney
Liver
Embryo
Thyroid

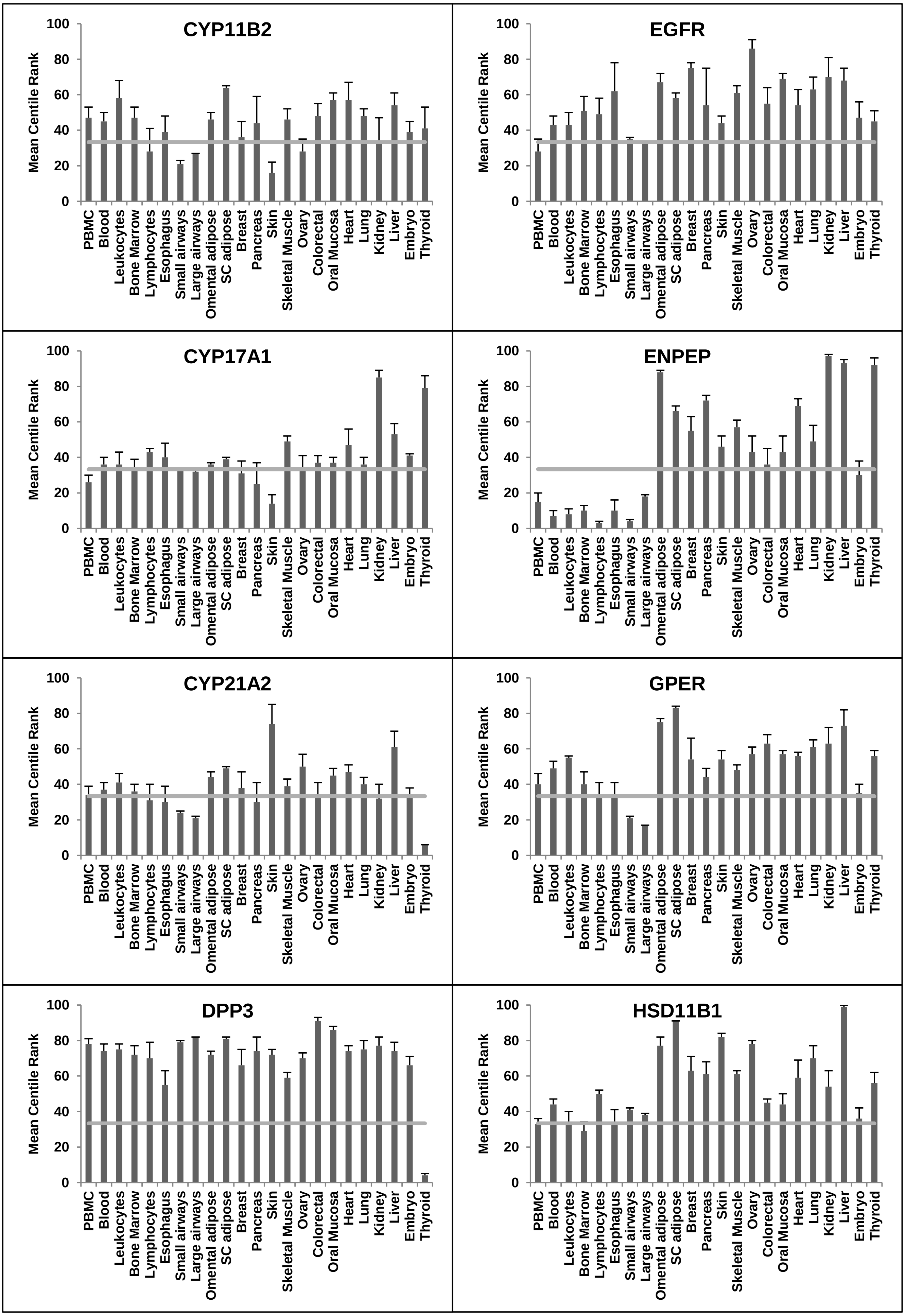
CYP11B2
EGFR
CYP17A1
ENPEP
CYP21A2
GPER
DPP3
HSD11B1
Mean Centile Rank
0
20
40
60
80
100
PBMC
Blood
Leukocytes
Bone Marrow
Lymphocytes
Esophagus
Small airways
Large airways
Omental adipose
SC adipose
Breast
Pancreas
Skin
Skeletal Muscle
Ovary
Colorectal
Oral Mucosa
Heart
Lung
Kidney
Liver
Embryo
Thyroid

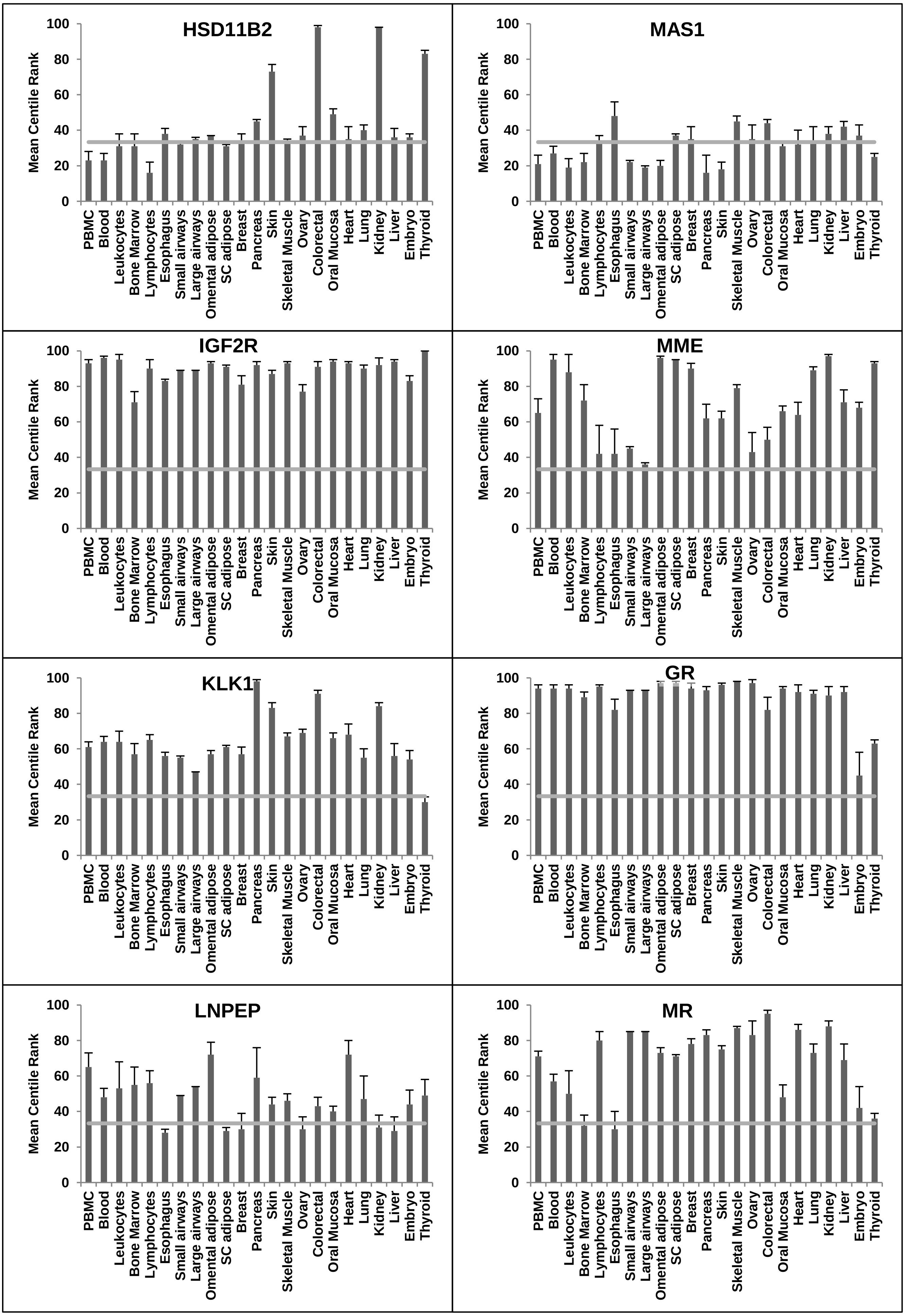
HSD11B2
MAS1
IGF2R
MME
KLK1
GR
LNPEP
MR
Mean Centile Rank
0
20
40
60
80
100
PBMC
Blood
Leukocytes
Bone Marrow
Lymphocytes
Esophagus
Small airways
Large airways
Omental adipose
SC adipose
Breast
Pancreas
Skin
Skeletal Muscle
Ovary
Colorectal
Oral Mucosa
Heart
Lung
Kidney
Liver
Embryo
Thyroid

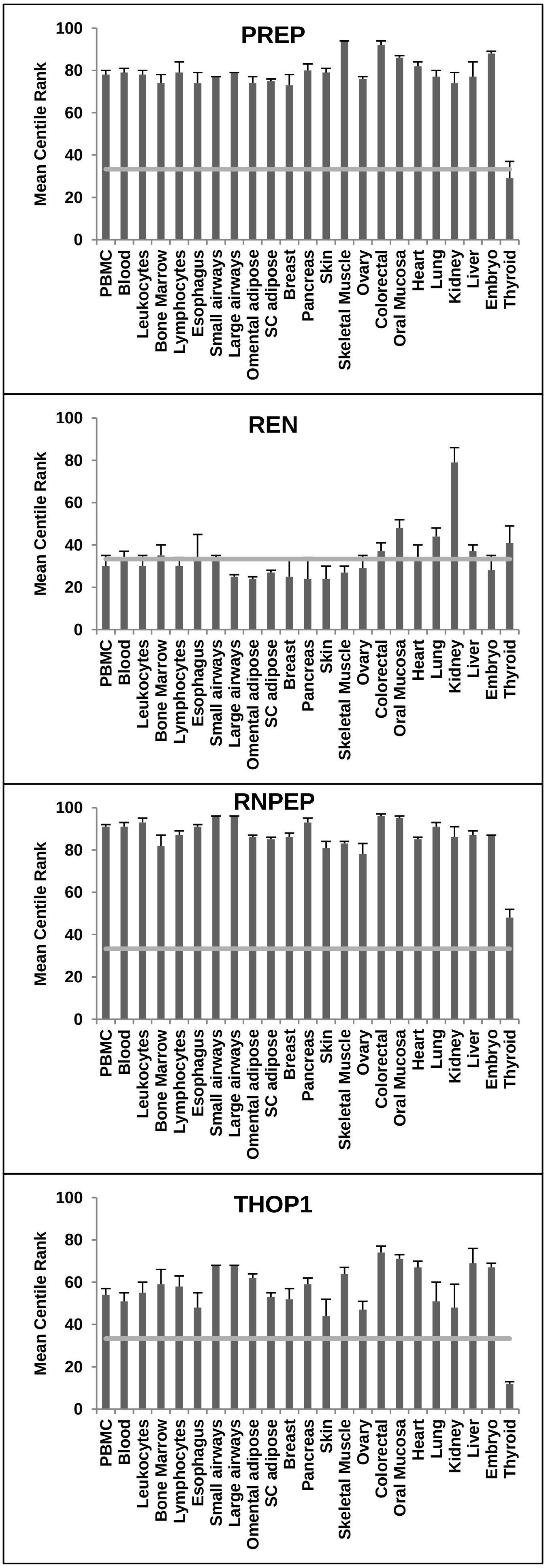
PREP
Mean Centile Rank
100
80
60
40
20
0
PBMC
Blood
Leukocytes
Bone Marrow
Lymphocytes
Esophagus
Small airways
Large airways
Omental adipose
SC adipose
Breast
Pancreas
Skin
Skeletal Muscle
Ovary
Colorectal
Oral Mucosa
Heart
Lung
Kidney
Liver
Embryo
Thyroid
REN
Mean Centile Rank
100
80
60
40
20
0
PBMC
Blood
Leukocytes
Bone Marrow
Lymphocytes
Esophagus
Small airways
Large airways
Omental adipose
SC adipose
Breast
Pancreas
Skin
Skeletal Muscle
Ovary
Colorectal
Oral Mucosa
Heart
Lung
Kidney
Liver
Embryo
Thyroid
RNPEP
Mean Centile Rank
100
80
60
40
20
0
PBMC
Blood
Leukocytes
Bone Marrow
Lymphocytes
Esophagus
Small airways
Large airways
Omental adipose
SC adipose
Breast
Pancreas
Skin
Skeletal Muscle
Ovary
Colorectal
Oral Mucosa
Heart
Lung
Kidney
Liver
Embryo
Thyroid
THOP1
Mean Centile Rank
100
80
60
40
20
0
PBMC
Blood
Leukocytes
Bone Marrow
Lymphocytes
Esophagus
Small airways
Large airways
Omental adipose
SC adipose
Breast
Pancreas
Skin
Skeletal Muscle
Ovary
Colorectal
Oral Mucosa
Heart
Lung
Kidney
Liver
Embryo
Thyroid

Supplementary Atlas S1: ExtRAAS Maps in All Studied Tissues

Supplementary Atlas S1: ExtRAAS maps in all studied tissues. For each tissue**,** the number of datasets, samples and modules are represented as (datasets, samples, modules) below the tissue name. Gene transcripts are represented by the corresponding official symbols. The genes are represented in the map based on their coordination (same color = same cluster) and mean expression centile rank (MCR, 4 levels, larger font size= higher expression level). Genes below the first tertile (MCR ≤ 33) in each tissue were omitted for simplicity. Non-clustered genes are colored in black. Core group-based modules when present are colored similarly across tissue: CTSA-CTSD-based modules in red; CTSG-CPA3-CMA1-based modules in green; and GR-MR-AGTR1-based modules in dark blue. Angiotensin peptides and corticosteroid metabolites are represented in gray italics. Expression profiles of ExtRAAS genes in each tissue are represented using their MCR in a bar graph. Tissues are arranged based on the tissue dendrogram of figure 5 in the article (see dendrogram below).

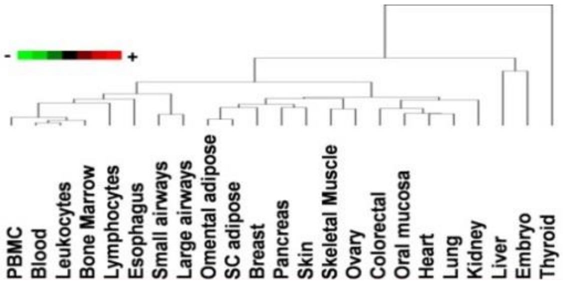

ExtRAAS

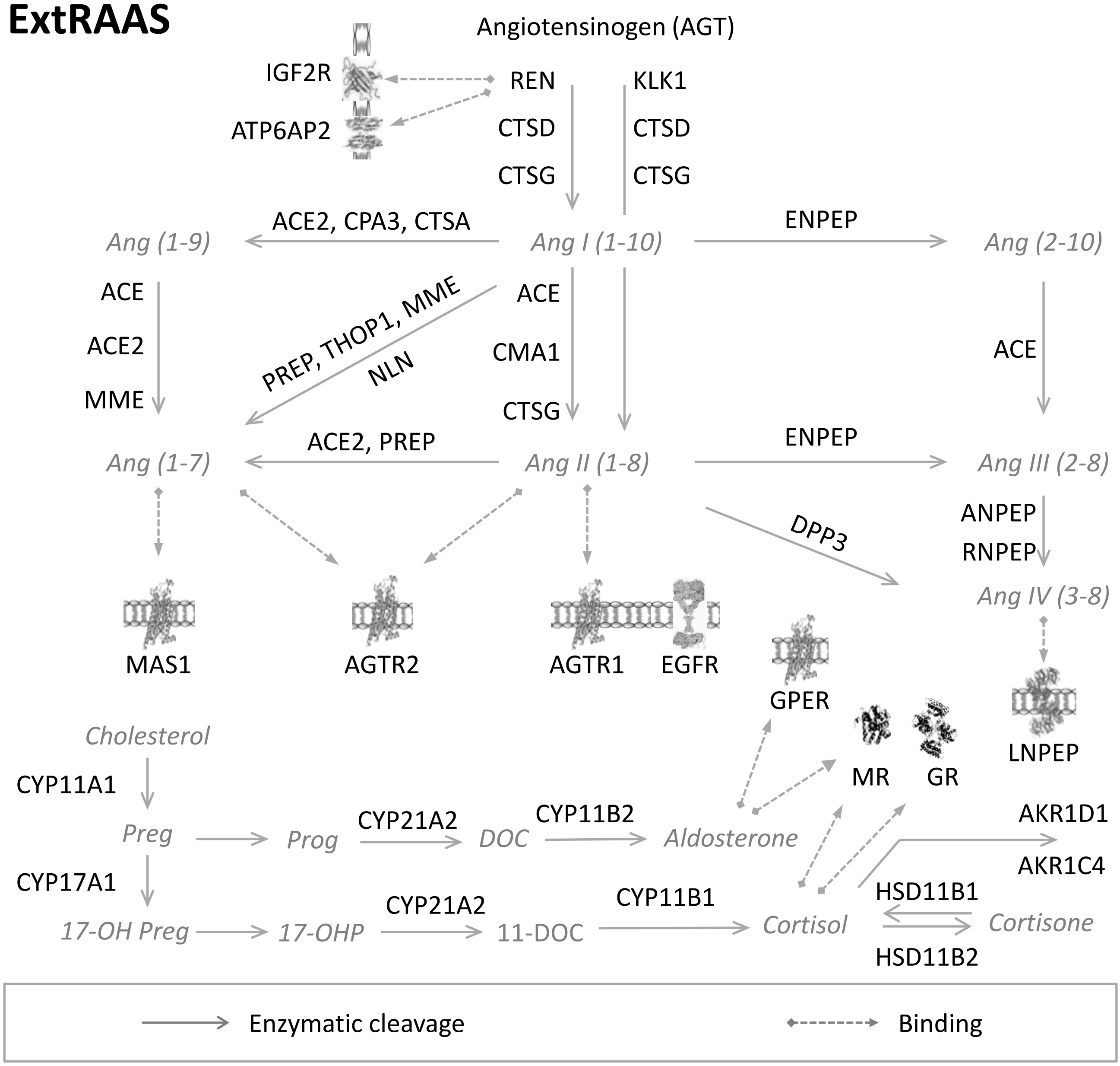

MCR font size

≤ 33 ➔ eleminated

34-40 ➔ 9

41-60 ➔ 11

61-80 ➔ 14

81-90 ➔ 18

91-100 ➔ 18

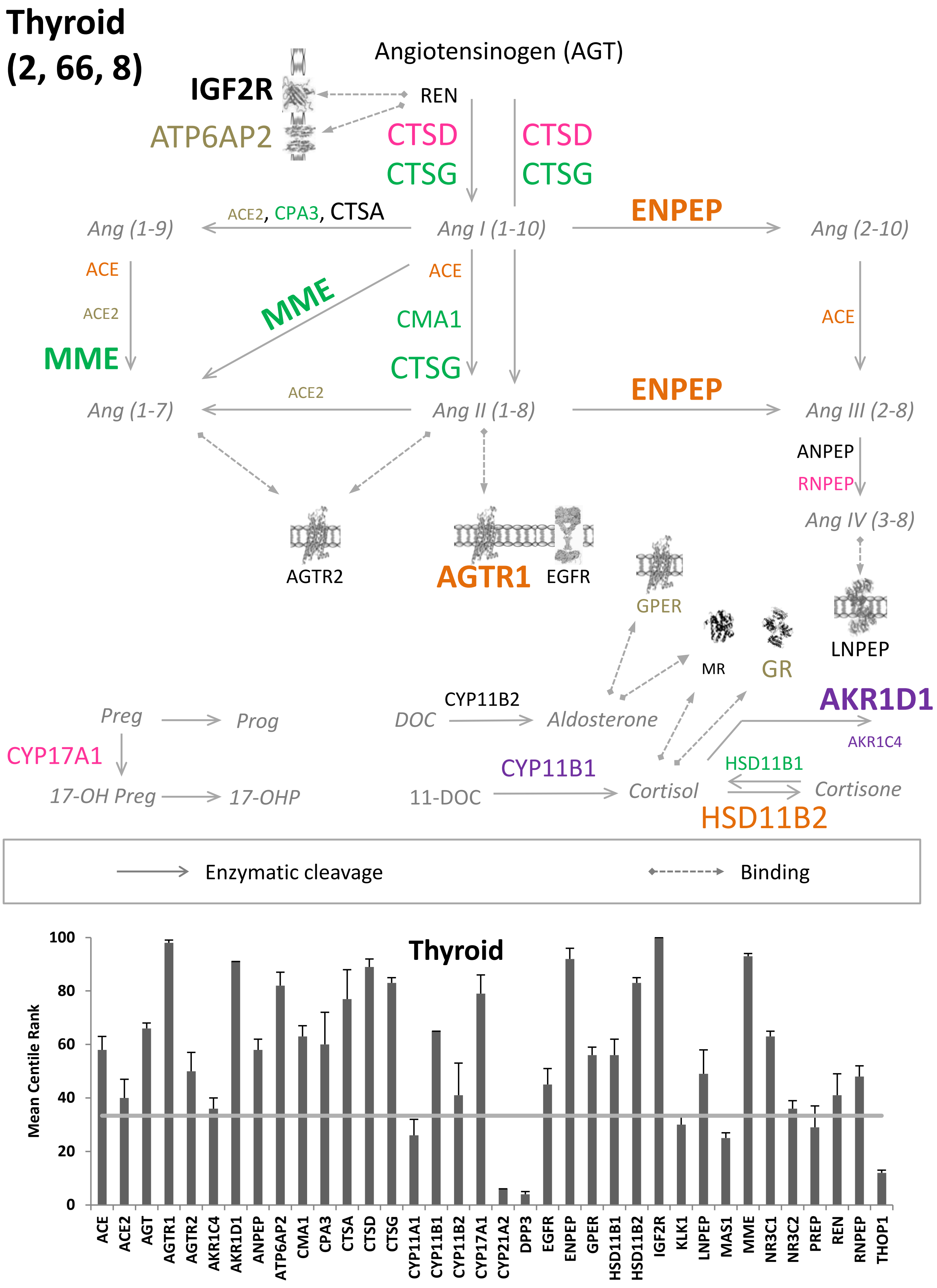
Thyroid
(2, 66, 8)
Angiotensinogen (AGT)
IGF2R
REN
ATP6AP2
CTSD
CTSG
CTSD
CTSG
ACE2, CPA3, CTSA
Ang (1-9)
Ang I (1-10)
ENPEP
Ang (2-10)
ACE
ACE2
MME
MME
ACE
CMA1
CTSG
ACE
ACE2
Ang (1-7)
Ang II (1-8)
ENPEP
Ang III (2-8)
ANPEP
RNPEP
Ang IV (3-8)
AGTR2
AGTR1
EGFR
GPER
MR
GR
LNPEP
AKR1D1
AKR1C4
Preg
Prog
DOC
CYP11B2
Aldosterone
CYP17A1
17-OH Preg
17-OHP
CYP11B1
11-DOC
Cortisol
HSD11B1
Cortisone
HSD11B2
Enzymatic cleavage
Binding
Thyroid
Mean Centile Rank
100
80
60
40
20
0
ACE
ACE2
AGT
AGTR1
AGTR2
AKR1C4
AKR1D1
ANPEP
ATP6AP2
CMA1
CPA3
CTSA
CTSD
CTSG
CYP11A1
CYP11B1
CYP11B2
CYP17A1
CYP21A2
DPP3
EGFR
ENPEP
GPER
HSD11B1
HSD11B2
IGF2R
KLK1
LNPEP
MAS1
MME
NR3C1
NR3C2
PREP
REN
RNPEP
THOP1

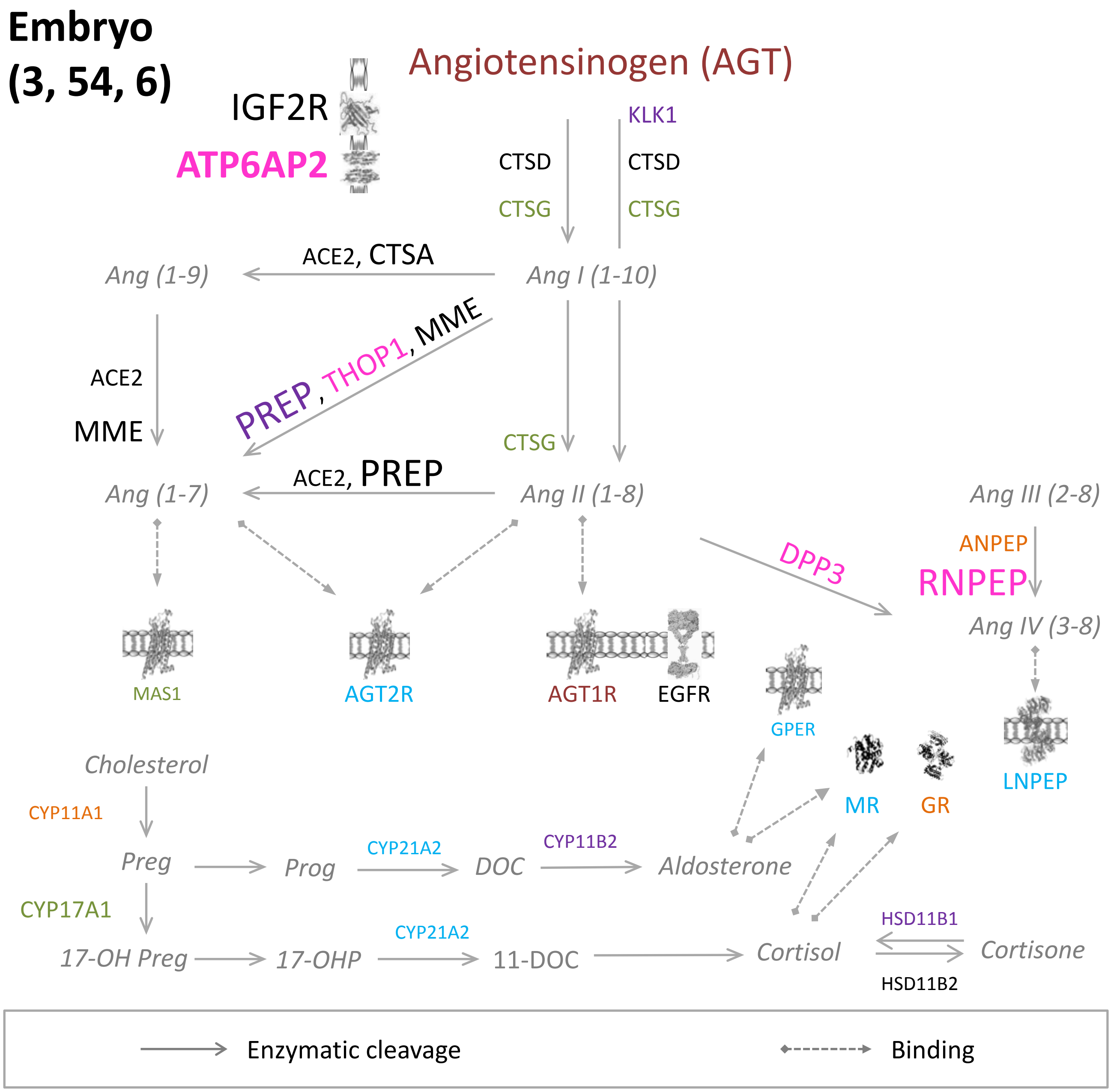

Embryo
(3, 54, 6)
IGF2R
ATP6AP2
Angiotensinogen (AGT)
KLK1
CTSD
CTSG
CTSD
CTSG
ACE2, CTSA
Ang (1-9)
Ang I (1-10)
ACE2
MME
PREP, THOP1, MME
CTSG
ACE2, PREP
Ang (1-7)
Ang II (1-8)
Ang III (2-8)
ANPEP
DPP3
RNPEP
Ang IV (3-8)
MAS1
AGT2R
AGT1R
EGFR
GPER
MR
GR
LNPEP
Cholesterol
CYP11A1
Preg
Prog
CYP21A2
DOC
CYP11B2
Aldosterone
CYP17A1
17-OH Preg
17-OHP
CYP21A2
11-DOC
Cortisol
HSD11B1
HSD11B2
Cortisone
Enzymatic cleavage
Binding

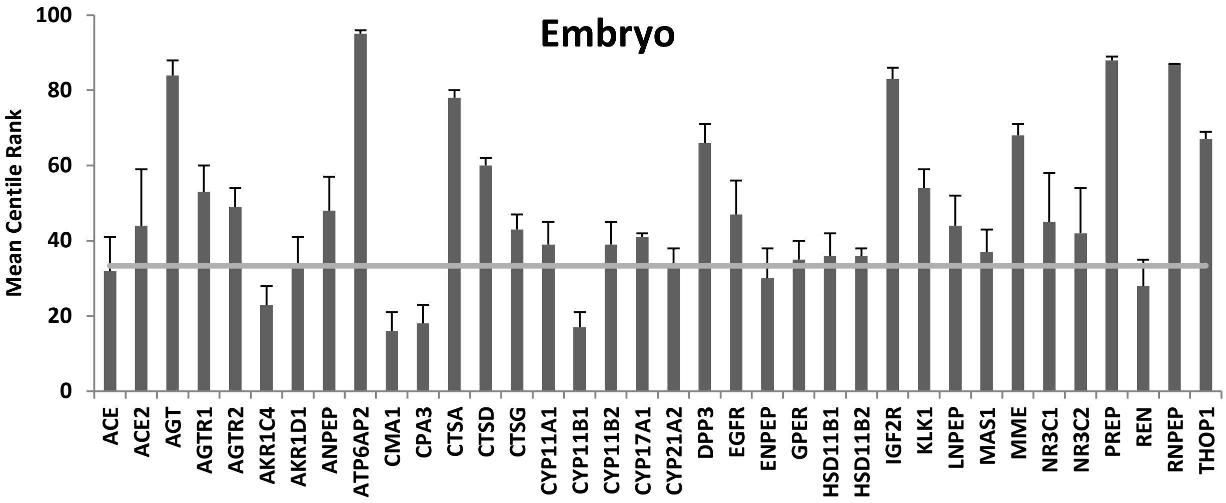

Embryo
Mean Centile Rank
100
80
60
40
20
0
ACE
ACE2
AGT
AGTR1
AGTR2
AKR1C4
AKR1D1
ANPEP
ATP6AP2
CMA1
CPA3
CTSA
CTSD
CTSG
CYP11A1
CYP11B1
CYP11B2
CYP17A1
CYP21A2
DPP3
EGFR
ENPEP
GPER
HSD11B1
HSD11B2
IGF2R
KLK1
LNPEP
MAS1
MME
NR3C1
NR3C2
PREP
REN
RNPEP
THOP1

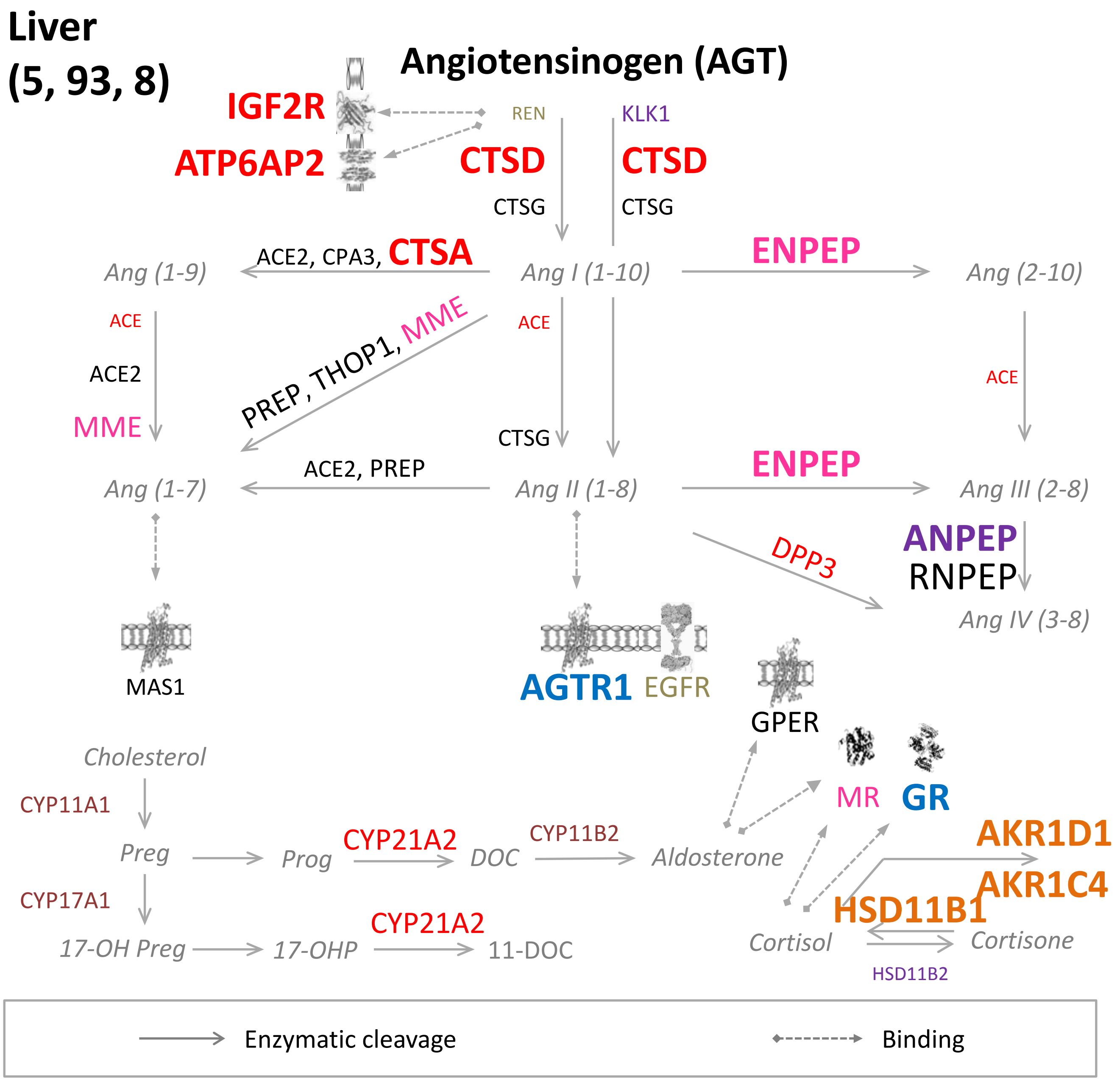

Liver
(5, 93, 8)
Angiotensinogen (AGT)
IGF2R
ATP6AP2
REN
KLK1
CTSD
CTSD
CTSG
CTSG
ACE2, CPA3, CTSA
Ang (1-9)
Ang I (1-10)
ENPEP
Ang (2-10)
ACE
ACE2
MME
PREP, THOP1, MME
ACE
CTSG
ACE
Ang (1-7)
ACE2, PREP
Ang II (1-8)
ENPEP
Ang III (2-8)
DPP3
ANPEP
RNPEP
Ang IV (3-8)
MAS1
AGTR1 EGFR
GPER
MR GR
Cholesterol
CYP11A1
Preg
Prog
CYP21A2
DOC
CYP11B2
Aldosterone
AKR1D1
AKR1C4
CYP17A1
HSD11B1
17-OH Preg
17-OHP
CYP21A2
11-DOC
Cortisol
Cortisone
HSD11B2
Enzymatic cleavage
Binding

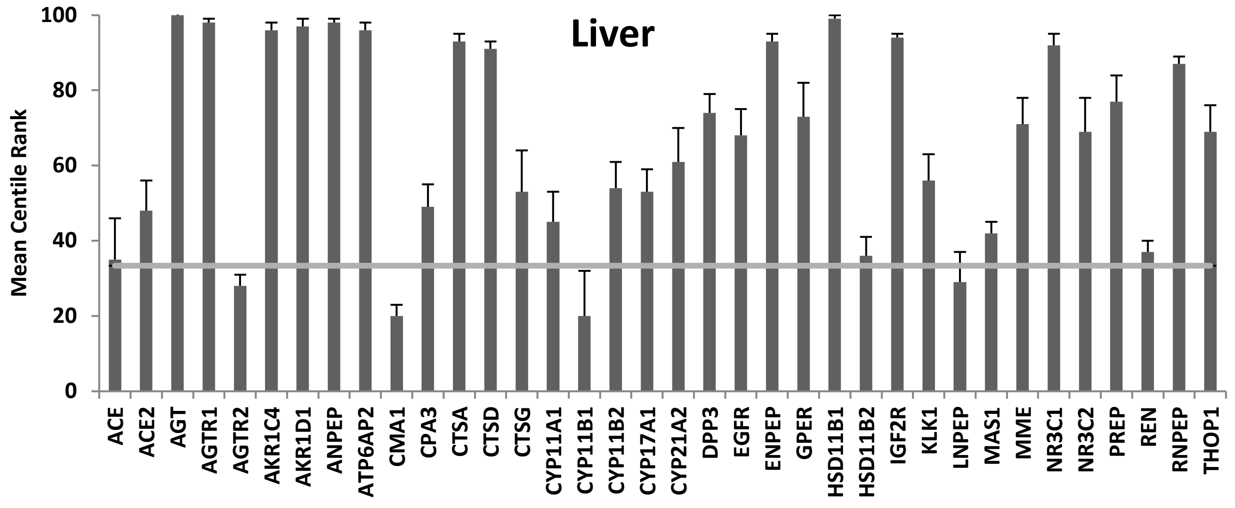

Liver
Mean Centile Rank
100
80
60
40
20
0
ACE
ACE2
AGT
AGTR1
AGTR2
AKR1C4
AKR1D1
ANPEP
ATP6AP2
CMA1
CPA3
CTSA
CTSD
CTSG
CYP11A1
CYP11B1
CYP11B2
CYP17A1
CYP21A2
DPP3
EGFR
ENPEP
GPER
HSD11B1
HSD11B2
IGF2R
KLK1
LNPEP
MAS1
MME
NR3C1
NR3C2
PREP
REN
RNPEP
THOP1

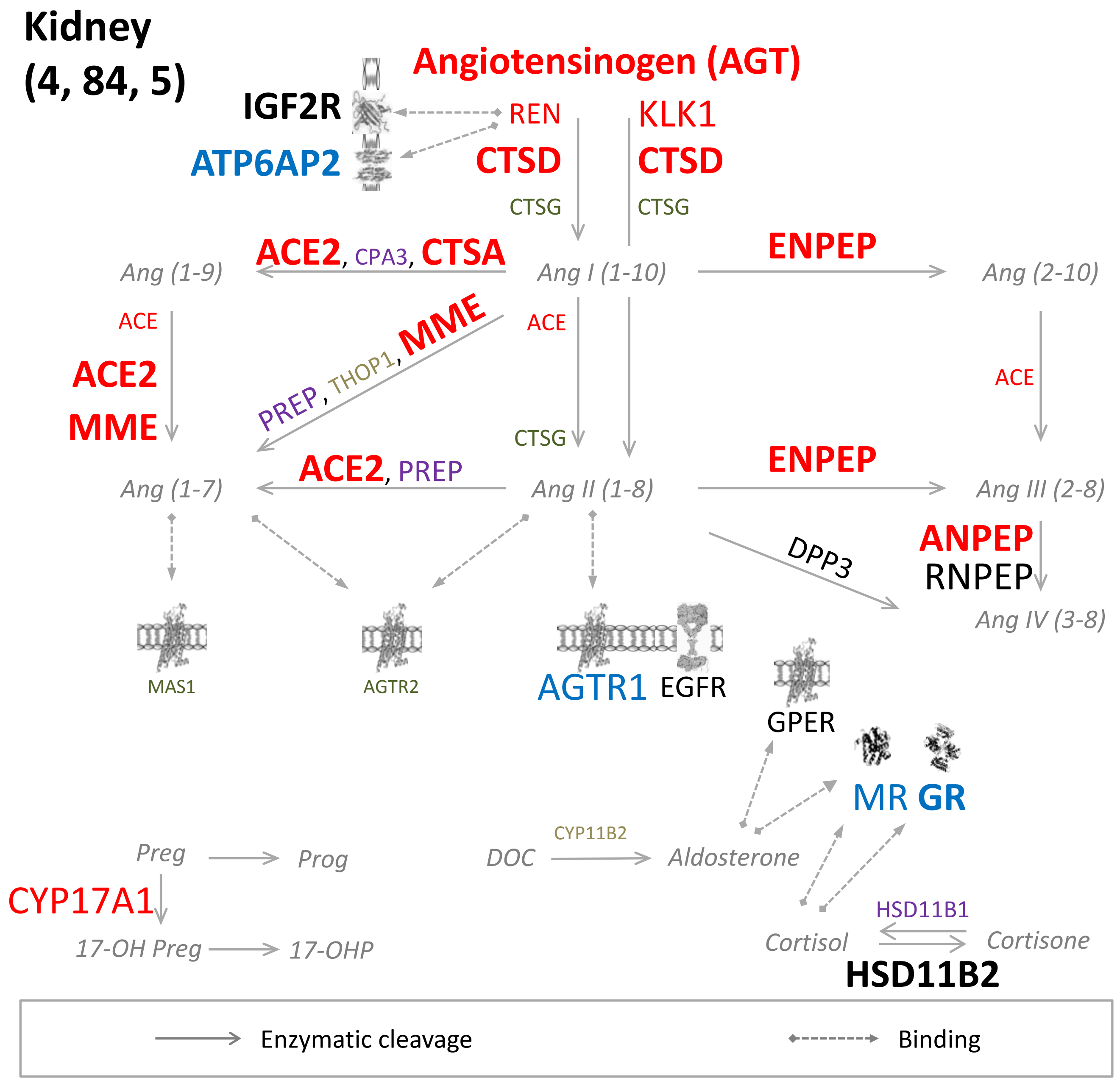
Kidney
(4, 84, 5)
Angiotensinogen (AGT)
IGF2R
ATP6AP2
REN
CTSD
CTSG
KLK1
CTSD
CTSG
Ang (1-9)
ACE2, CPA3, CTSA
Ang I (1-10)
ENPEP
Ang (2-10)
ACE
ACE2
MME
PREP, THOP1, MME
ACE
CTSG
ACE
Ang (1-7)
ACE2, PREP
Ang II (1-8)
ENPEP
Ang III (2-8)
DPP3
ANPEP
RNPEP
Ang IV (3-8)
MAS1
AGTR2
AGTR1
EGFR
GPER
MR GR
Preg
Prog
DOC
CYP11B2
Aldosterone
CYP17A1
17-OH Preg
17-OHP
HSD11B1
Cortisol
Cortisone
HSD11B2
Enzymatic cleavage
Binding

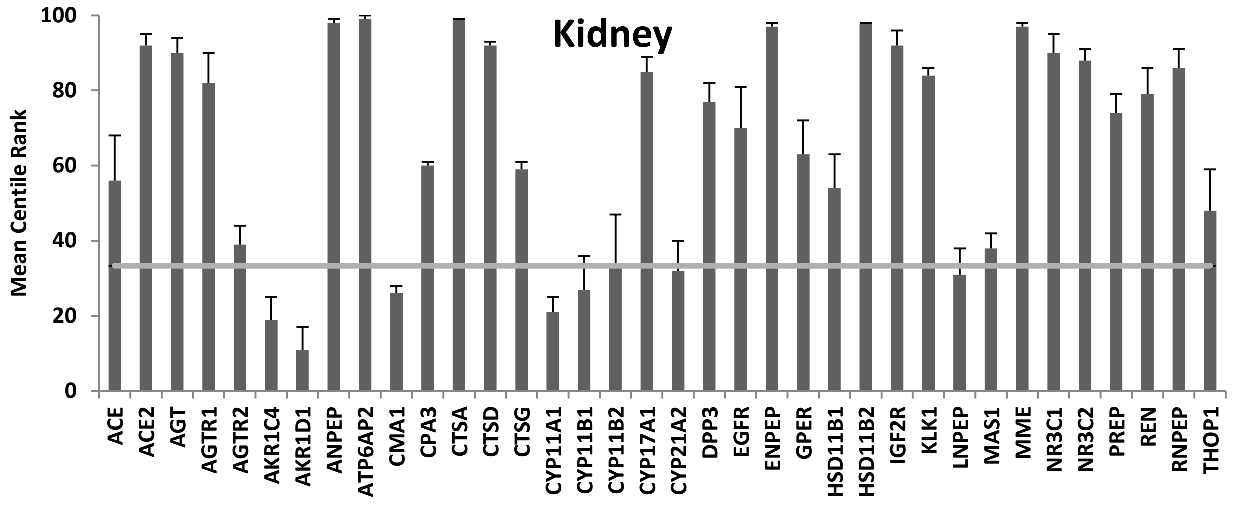
Kidney
Mean Centile Rank
0
20
40
60
80
100
ACE
ACE2
AGT
AGTR1
AGTR2
AKR1C4
AKR1D1
ANPEP
ATP6AP2
CMA1
CPA3
CTSA
CTSD
CTSG
CYP11A1
CYP11B1
CYP11B2
CYP17A1
CYP21A2
DPP3
EGFR
ENPEP
GPER
HSD11B1
HSD11B2
IGF2R
KLK1
LNPEP
MAS1
MME
NR3C1
NR3C2
PREP
REN
RNPEP
THOP1

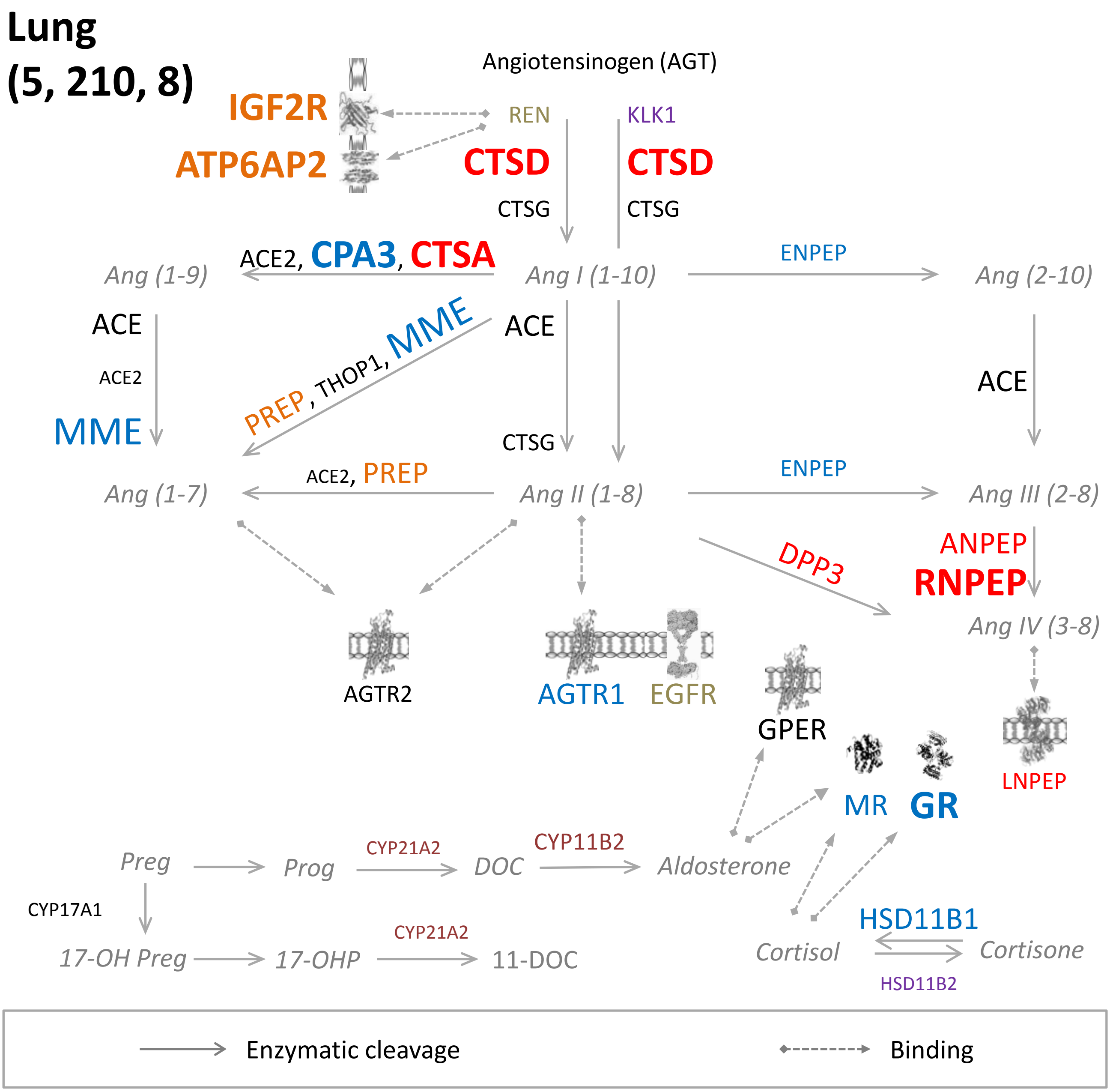

Lung
(5, 210, 8)
Angiotensinogen (AGT)
IGF2R
ATP6AP2
REN
KLK1
CTSD
CTSD
CTSG
CTSG
ACE2, CPA3, CTSA
Ang (1-9)
Ang I (1-10)
ENPEP
Ang (2-10)
ACE
ACE2
MME
PREP, THOP1, MME
ACE
ACE
CTSG
ACE2, PREP
Ang (1-7)
Ang II (1-8)
ENPEP
Ang III (2-8)
DPP3
ANPEP
RNPEP
Ang IV (3-8)
AGTR2
AGTR1
EGFR
GPER
MR
GR
LNPEP
Preg
Prog
CYP21A2
DOC
CYP11B2
Aldosterone
CYP17A1
17-OH Preg
17-OHP
CYP21A2
11-DOC
HSD11B1
Cortisol
Cortisone
HSD11B2
Enzymatic cleavage
Binding

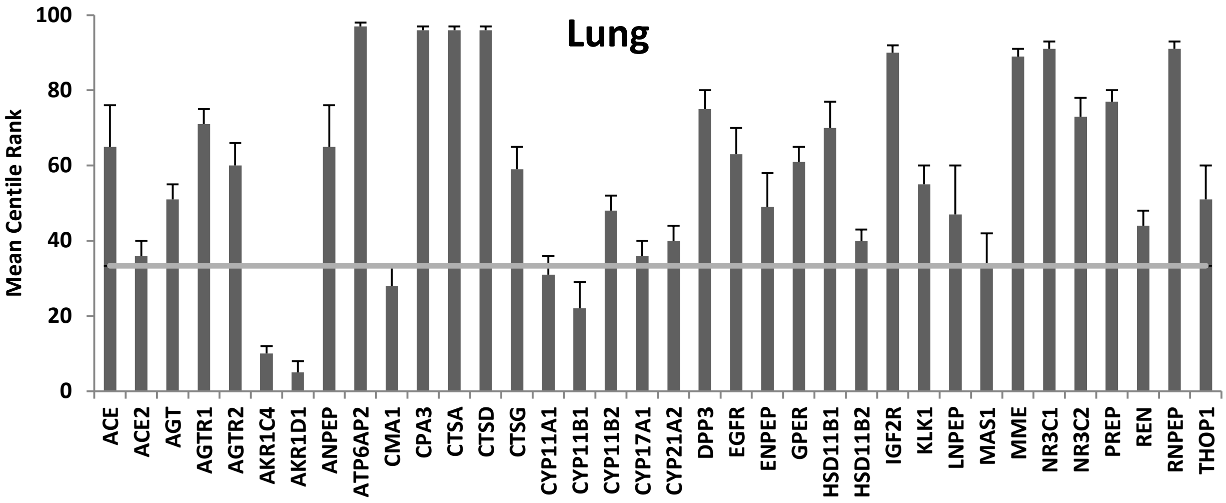

Lung
Mean Centile Rank
100
80
60
40
20
0
ACE
ACE2
AGT
AGTR1
AGTR2
AKR1C4
AKR1D1
ANPEP
ATP6AP2
CMA1
CPA3
CTSA
CTSD
CTSG
CYP11A1
CYP11B1
CYP11B2
CYP17A1
CYP21A2
DPP3
EGFR
ENPEP
GPER
HSD11B1
HSD11B2
IGF2R
KLK1
LNPEP
MAS1
MME
NR3C1
NR3C2
PREP
REN
RNPEP
THOP1

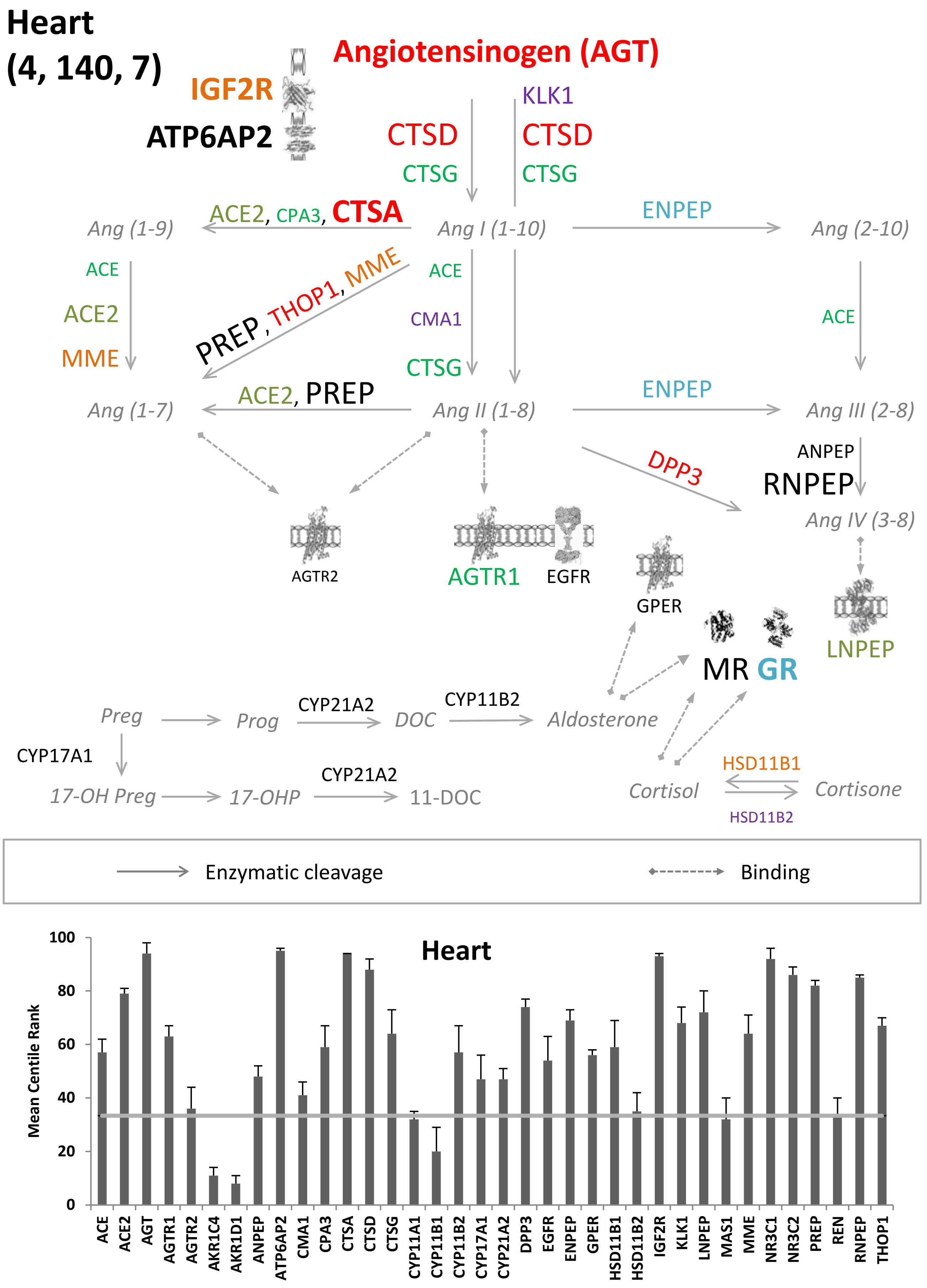
Heart
(4, 140, 7)
IGF2R
ATP6AP2
Angiotensinogen (AGT)
KLK1
CTSD
CTSD
CTSG
CTSG
ACE2, CPA3, CTSA
Ang (1-9)
Ang I (1-10)
ENPEP
Ang (2-10)
ACE
ACE2
MME
PREP, THOP1, MME
ACE
CMA1
CTSG
ACE
ACE2, PREP
Ang (1-7)
Ang II (1-8)
ENPEP
Ang III (2-8)
DPP3
ANPEP
RNPEP
Ang IV (3-8)
AGTR2
AGTR1
EGFR
GPER
MR GR
LNPEP
Preg
Prog
CYP21A2
DOC
CYP11B2
Aldosterone
CYP17A1
17-OH Preg
17-OHP
CYP21A2
11-DOC
Cortisol
HSD11B1
HSD11B2
Cortisone
Enzymatic cleavage
Binding
Heart
Mean Centile Rank
100
80
60
40
20
0
ACE
ACE2
AGT
AGTR1
AGTR2
AKR1C4
AKR1D1
ANPEP
ATP6AP2
CMA1
CPA3
CTSA
CTSD
CTSG
CYP11A1
CYP11B1
CYP11B2
CYP17A1
CYP21A2
DPP3
EGFR
ENPEP
GPER
HSD11B1
HSD11B2
IGF2R
KLK1
LNPEP
MAS1
MME
NR3C1
NR3C2
PREP
REN
RNPEP
THOP1

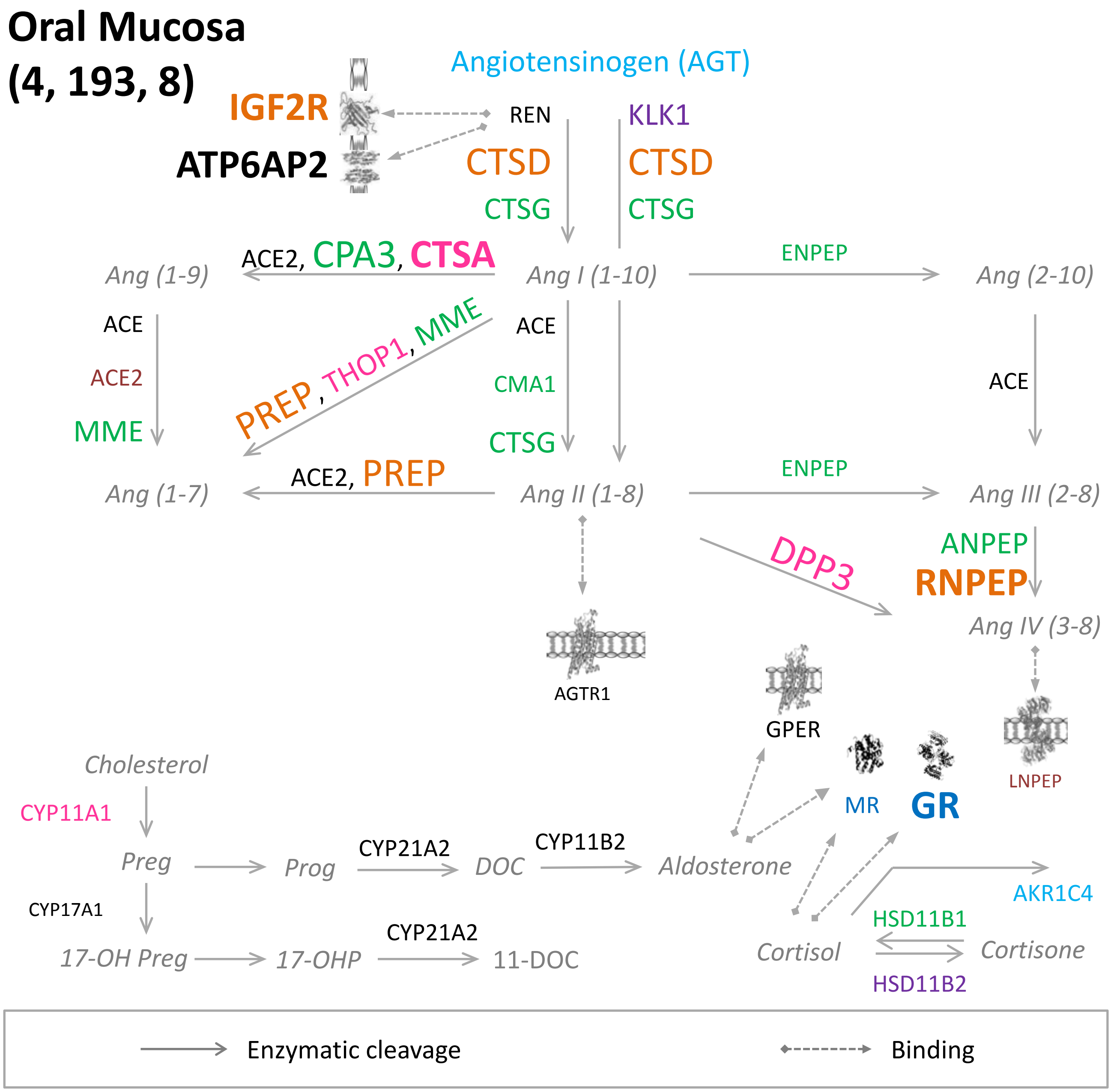
Oral Mucosa
(4, 193, 8)
Angiotensinogen (AGT)
IGF2R
ATP6AP2
REN
KLK1
CTSD
CTSD
CTSG
CTSG
ACE2, CPA3, CTSA
Ang (1-9)
Ang I (1-10)
ENPEP
Ang (2-10)
ACE
ACE2
MME
PREP, THOP1, MME
ACE
CMA1
CTSG
ACE
ACE2, PREP
Ang (1-7)
Ang II (1-8)
ENPEP
Ang III (2-8)
DPP3
ANPEP
RNPEP
Ang IV (3-8)
AGTR1
GPER
MR
GR
LNPEP
Cholesterol
CYP11A1
Preg
Prog
CYP21A2
DOC
CYP11B2
Aldosterone
CYP17A1
17-OH Preg
17-OHP
CYP21A2
11-DOC
AKR1C4
HSD11B1
Cortisol
Cortisone
HSD11B2
Enzymatic cleavage
Binding

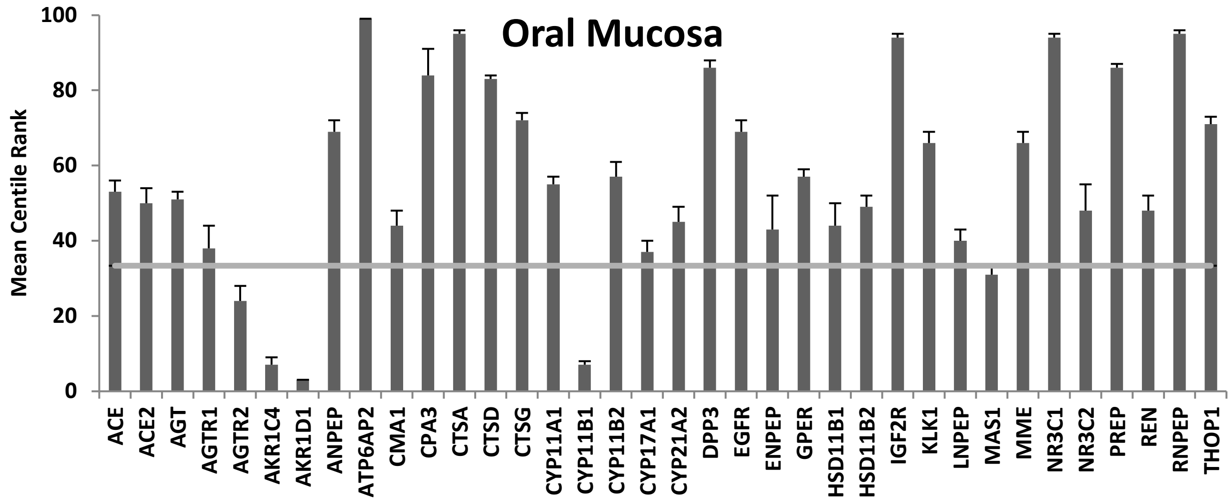
Oral Mucosa
Mean Centile Rank
100
80
60
40
20
0
ACE
ACE2
AGT
AGTR1
AGTR2
AKR1C4
AKR1D1
ANPEP
ATP6AP2
CMA1
CPA3
CTSA
CTSD
CTSG
CYP11A1
CYP11B1
CYP11B2
CYP17A1
CYP21A2
DPP3
EGFR
ENPEP
GPER
HSD11B1
HSD11B2
IGF2R
KLK1
LNPEP
MAS1
MME
NR3C1
NR3C2
PREP
REN
RNPEP
THOP1

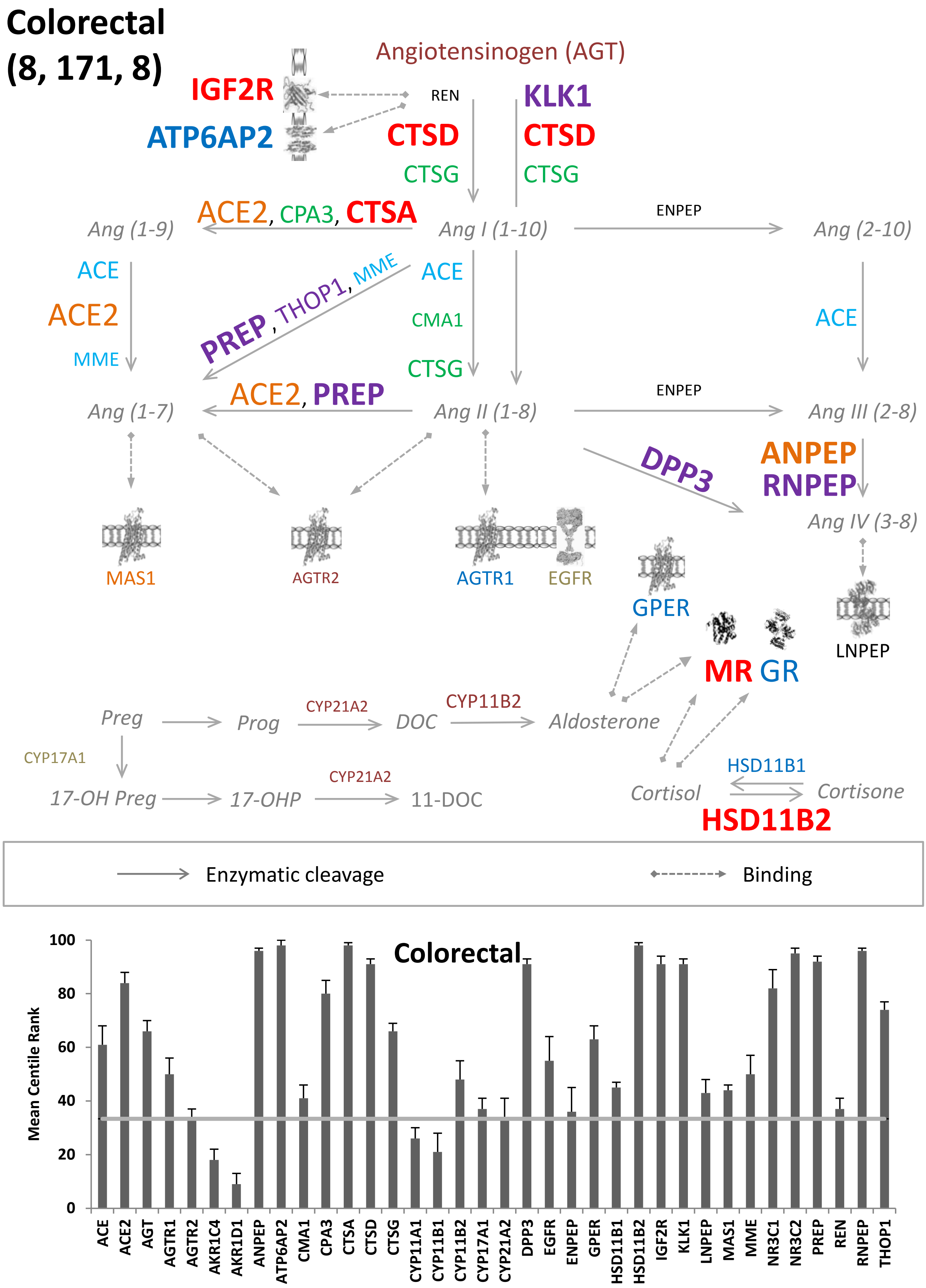
Colorectal
(8, 171, 8)
Angiotensinogen (AGT)
IGF2R
ATP6AP2
REN
KLK1
CTSD
CTSD
CTSG
CTSG
ACE2, CPA3, CTSA
Ang (1-9)
Ang I (1-10)
ENPEP
Ang (2-10)
ACE
ACE2
MME
PREP, THOP1, MME
ACE
CMA1
CTSG
ACE
Ang (1-7)
ACE2, PREP
Ang II (1-8)
ENPEP
Ang III (2-8)
DPP3
ANPEP
RNPEP
Ang IV (3-8)
MAS1
AGTR2
AGTR1
EGFR
GPER
MR
GR
LNPEP
Preg
Prog
CYP21A2
DOC
CYP11B2
Aldosterone
CYP17A1
17-OH Preg
17-OHP
CYP21A2
11-DOC
Cortisol
HSD11B1
Cortisone
HSD11B2
Enzymatic cleavage
Binding
Colorectal
Mean Centile Rank
100
80
60
40
20
0
ACE
ACE2
AGT
AGTR1
AGTR2
AKR1C4
AKR1D1
ANPEP
ATP6AP2
CMA1
CPA3
CTSA
CTSD
CTSG
CYP11A1
CYP11B1
CYP11B2
CYP17A1
CYP21A2
DPP3
EGFR
ENPEP
GPER
HSD11B1
HSD11B2
IGF2R
KLK1
LNPEP
MAS1
MME
NR3C1
NR3C2
PREP
REN
RNPEP
THOP1

Ovary Epithelium
(5, 55, 8)

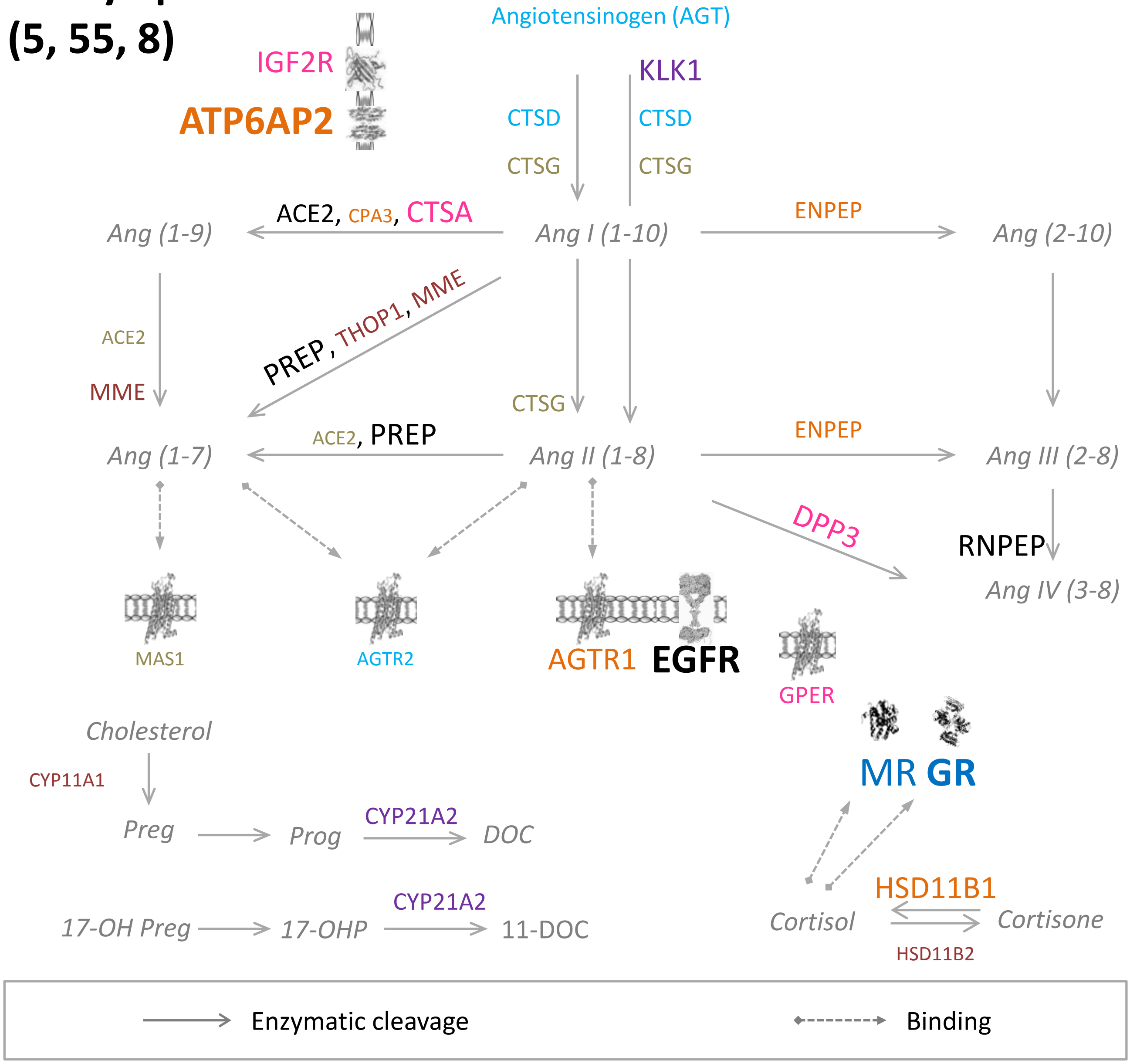

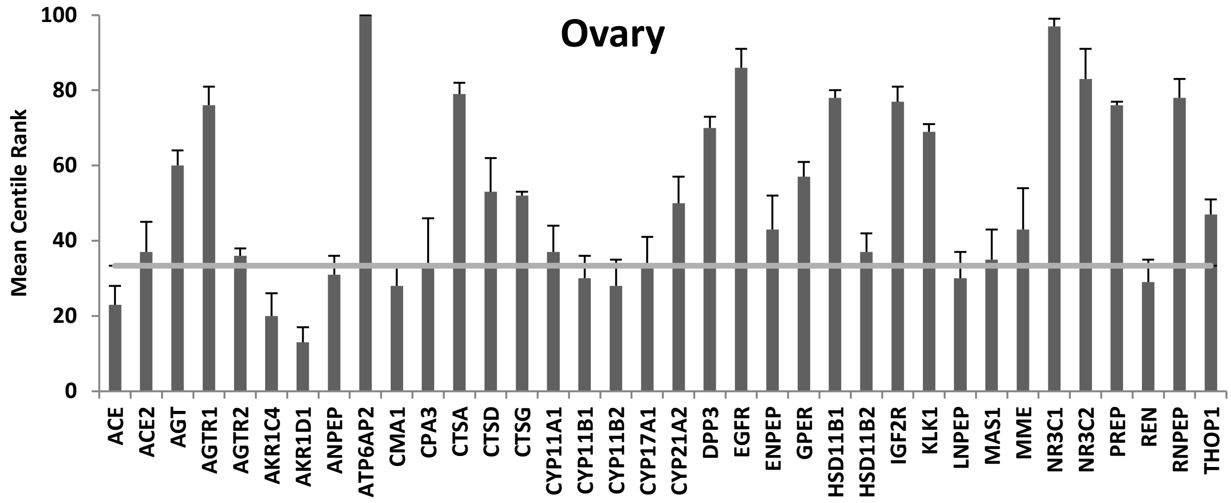

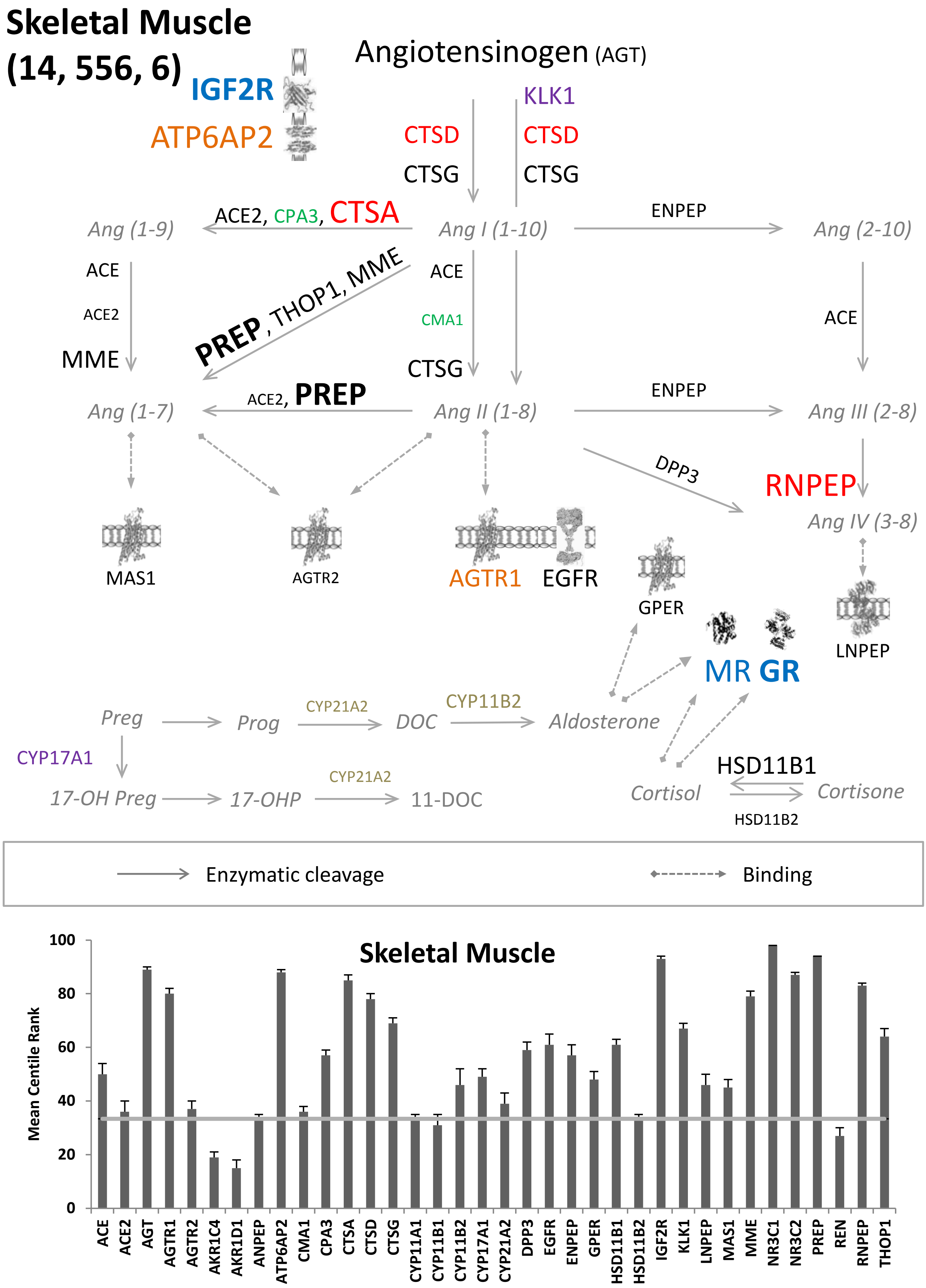

Skeletal Muscle
(14, 556, 6)
IGF2R
ATP6AP2
Angiotensinogen (AGT)
KLK1
CTSD
CTSG
CTSD
CTSG
ACE2, CPA3, CTSA
Ang (1-9)
Ang I (1-10)
ENPEP
Ang (2-10)
ACE
ACE2
MME
PREP, THOP1, MME
ACE
CMA1
CTSG
ACE
ACE2, PREP
Ang (1-7)
Ang II (1-8)
ENPEP
Ang III (2-8)
DPP3
RNPEP
Ang IV (3-8)
MAS1
AGTR2
AGTR1
EGFR
GPER
LNPEP
MR GR
Preg
Prog
CYP21A2
DOC
CYP11B2
Aldosterone
CYP17A1
17-OH Preg
17-OHP
CYP21A2
11-DOC
HSD11B1
Cortisol
Cortisone
HSD11B2
Enzymatic cleavage
Binding
Skeletal Muscle
Mean Centile Rank
100
80
60
40
20
0
ACE
ACE2
AGT
AGTR1
AGTR2
AKR1C4
AKR1D1
ANPEP
ATP6AP2
CMA1
CPA3
CTSA
CTSD
CTSG
CYP11A1
CYP11B1
CYP11B2
CYP17A1
CYP21A2
DPP3
EGFR
ENPEP
GPER
HSD11B1
HSD11B2
IGF2R
KLK1
LNPEP
MAS1
MME
NR3C1
NR3C2
PREP
REN
RNPEP
THOP1

Skin
(7, 222, 6)

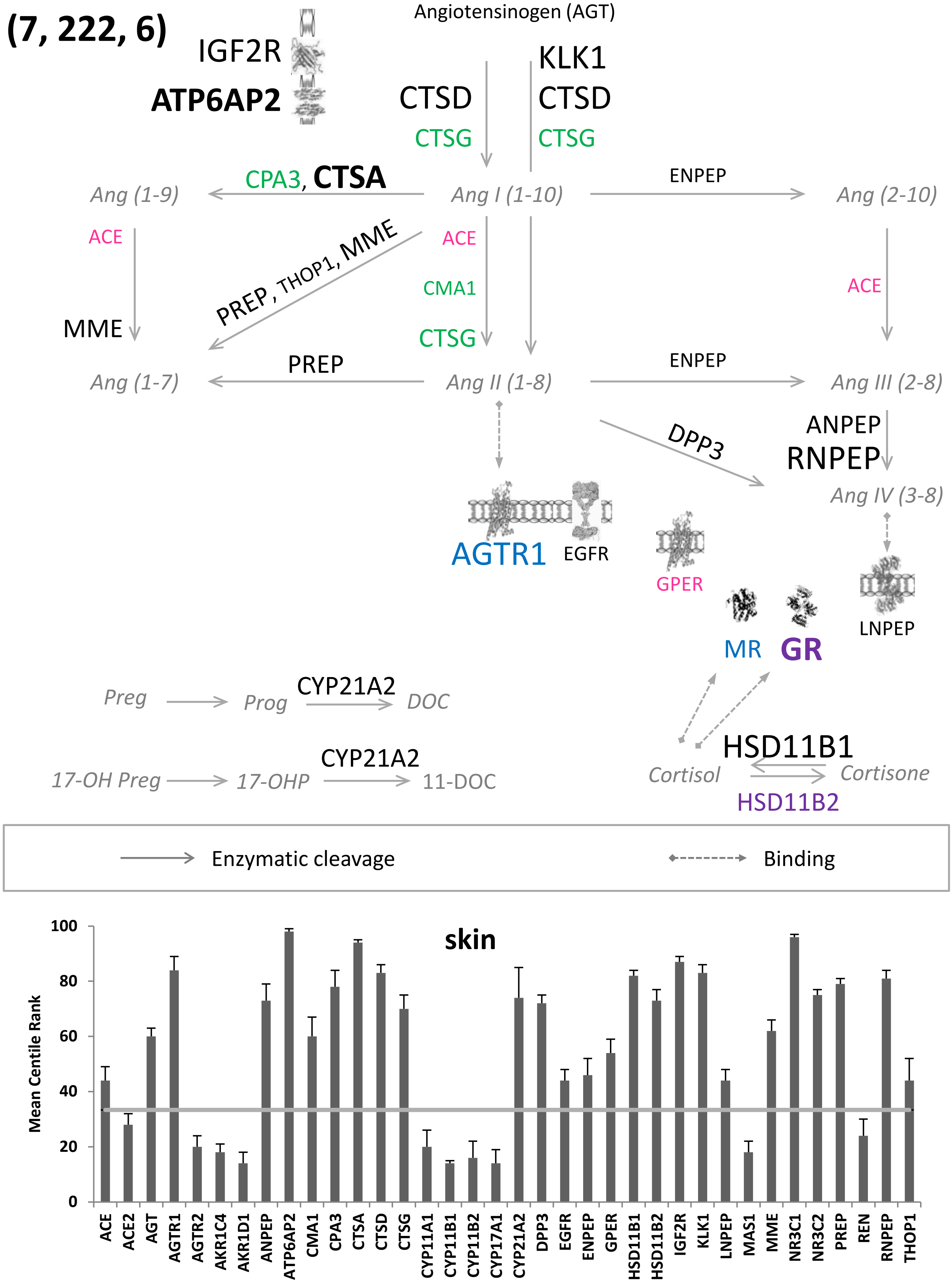

Angiotensinogen (AGT)
IGF2R
ATP6AP2
KLK1
CTSD
CTSG
CTSD
CTSG
CPA3, CTSA
Ang (1-9)
Ang I (1-10)
ENPEP
Ang (2-10)
ACE
PREP, THOP1, MME
ACE
CMA1
CTSG
ACE
MME
Ang (1-7)
PREP
Ang II (1-8)
ENPEP
Ang III (2-8)
DPP3
ANPEP
RNPEP
Ang IV (3-8)
AGTR1
EGFR
GPER
MR
GR
LNPEP
Preg
Prog
CYP21A2
DOC
17-OH Preg
17-OHP
CYP21A2
11-DOC
Cortisol
HSD11B1
Cortisone
HSD11B2
Enzymatic cleavage
Binding
skin
Mean Centile Rank
100
80
60
40
20
0
ACE
ACE2
AGT
AGTR1
AGTR2
AKR1C4
AKR1D1
ANPEP
ATP6AP2
CMA1
CPA3
CTSA
CTSD
CTSG
CYP11A1
CYP11B1
CYP11B2
CYP17A1
CYP21A2
DPP3
EGFR
ENPEP
GPER
HSD11B1
HSD11B2
IGF2R
KLK1
LNPEP
MAS1
MME
NR3C1
NR3C2
PREP
REN
RNPEP
THOP1

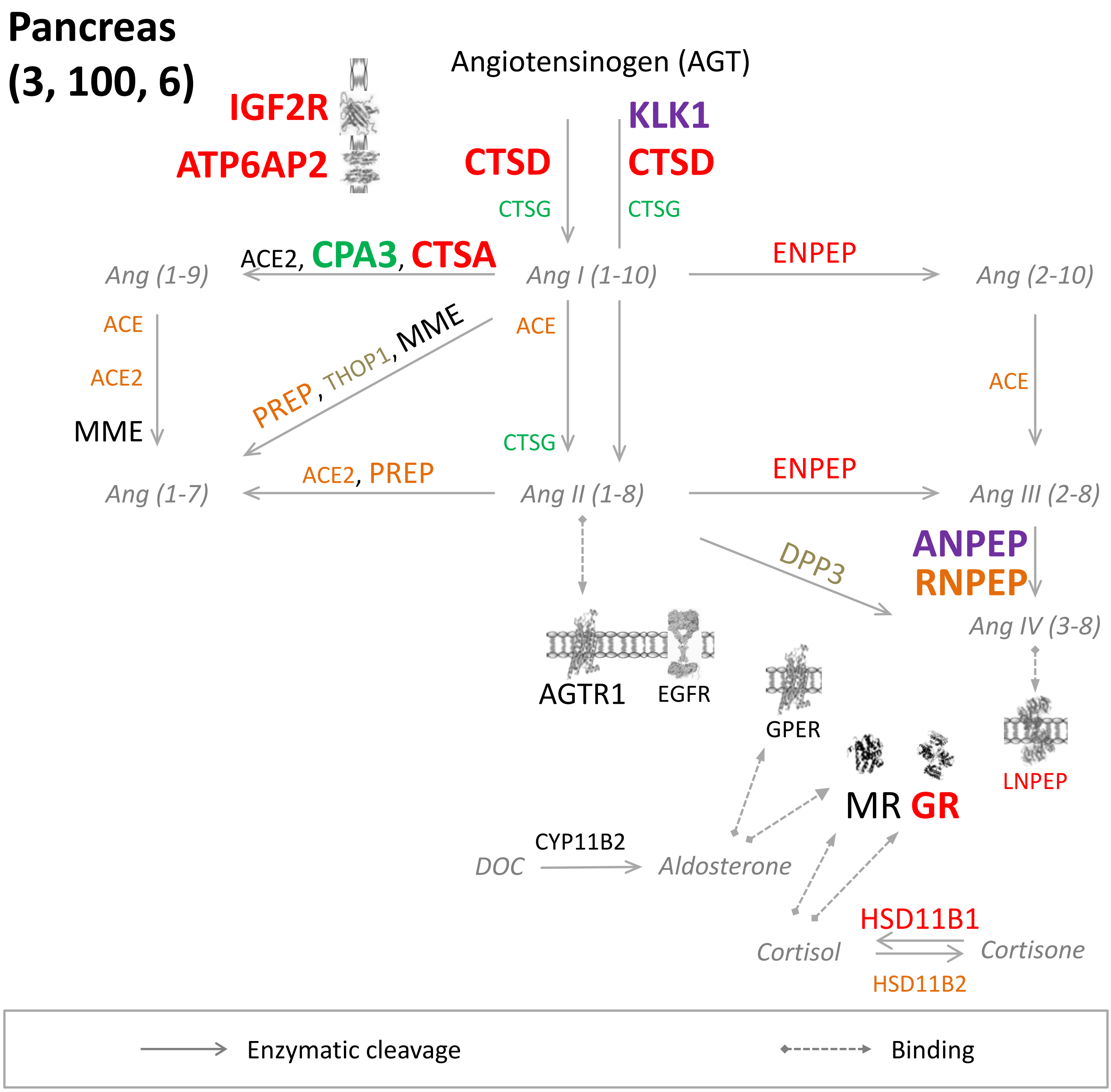
Pancreas
(3, 100, 6)
Angiotensinogen (AGT)
IGF2R
ATP6AP2
KLK1
CTSD
CTSD
CTSG
CTSG
ACE2, CPA3, CTSA
ENPEP
Ang (1-9)
Ang I (1-10)
Ang (2-10)
ACE
ACE2
MME
PREP, THOP1, MME
ACE
ACE
CTSG
ACE2, PREP
ENPEP
Ang (1-7)
Ang II (1-8)
Ang III (2-8)
DPP3
ANPEP
RNPEP
Ang IV (3-8)
AGTR1
EGFR
GPER
MR GR
LNPEP
CYP11B2
DOC
Aldosterone
HSD11B1
Cortisol
Cortisone
HSD11B2
Enzymatic cleavage
Binding

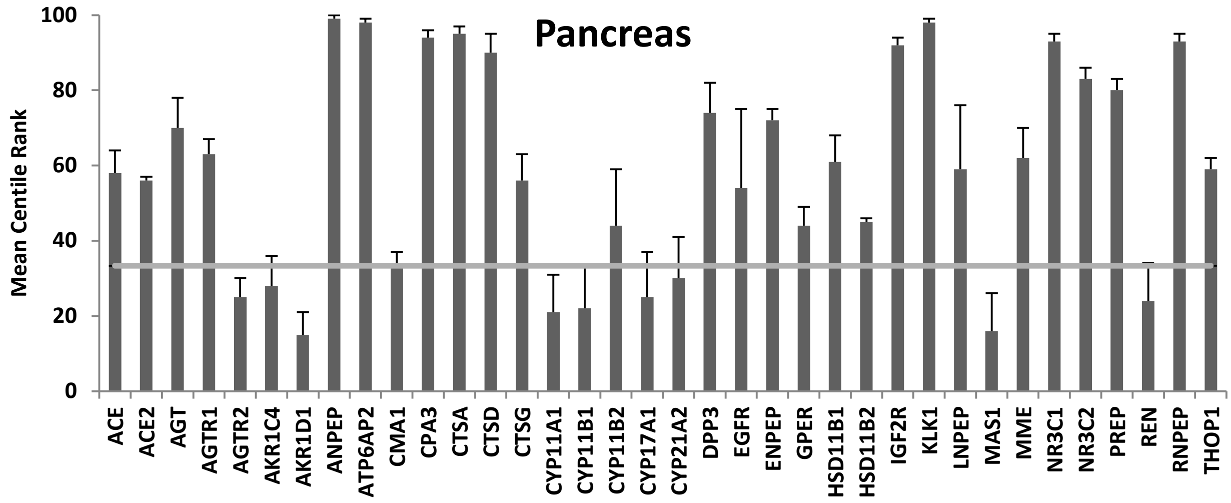
Pancreas
Mean Centile Rank
100
80
60
40
20
0
ACE
ACE2
AGT
AGTR1
AGTR2
AKR1C4
AKR1D1
ANPEP
ATP6AP2
CMA1
CPA3
CTSA
CTSD
CTSG
CYP11A1
CYP11B1
CYP11B2
CYP17A1
CYP21A2
DPP3
EGFR
ENPEP
GPER
HSD11B1
HSD11B2
IGF2R
KLK1
LNPEP
MAS1
MME
NR3C1
NR3C2
PREP
REN
RNPEP
THOP1

Breast

(6, 239, 7)

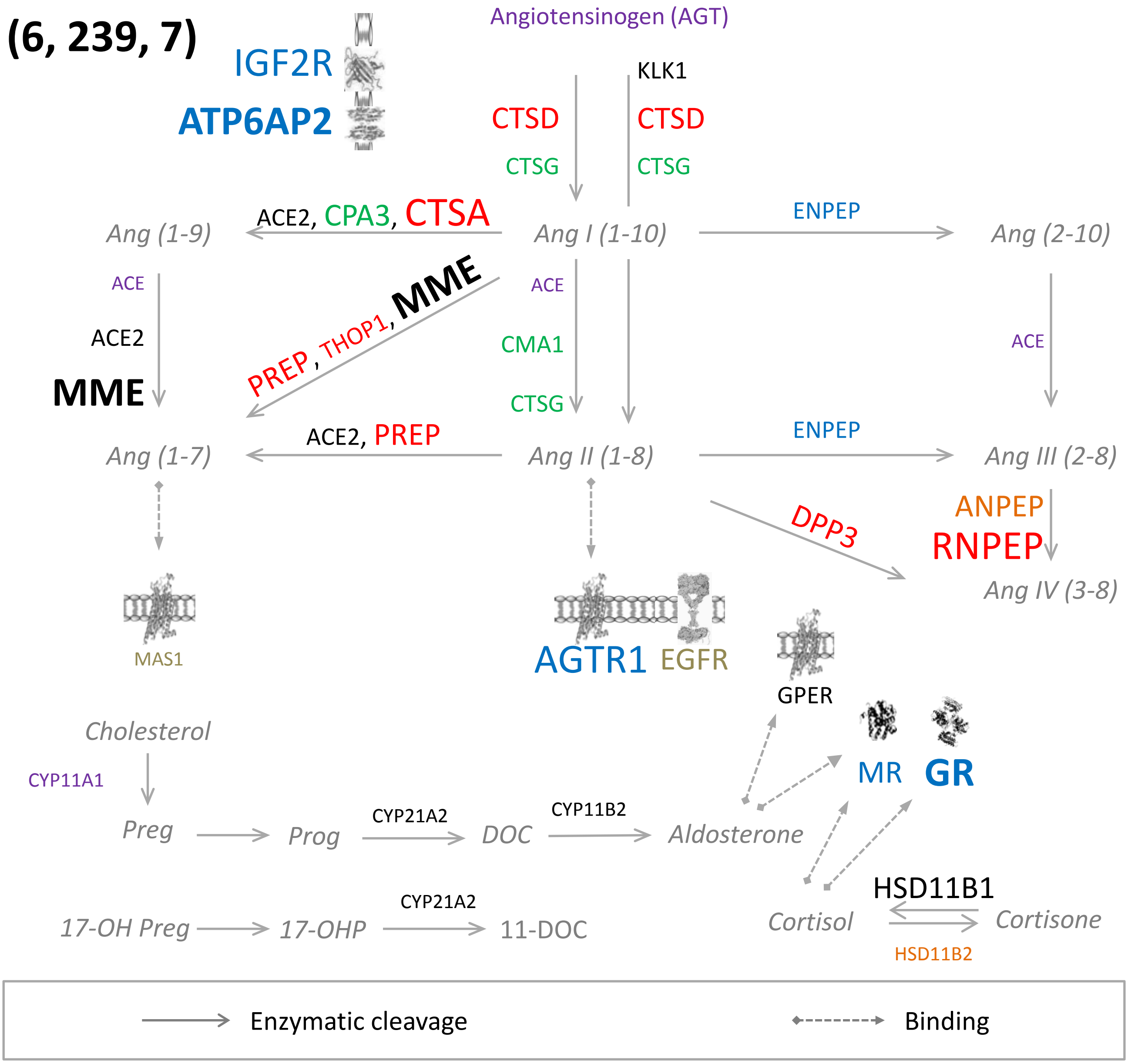

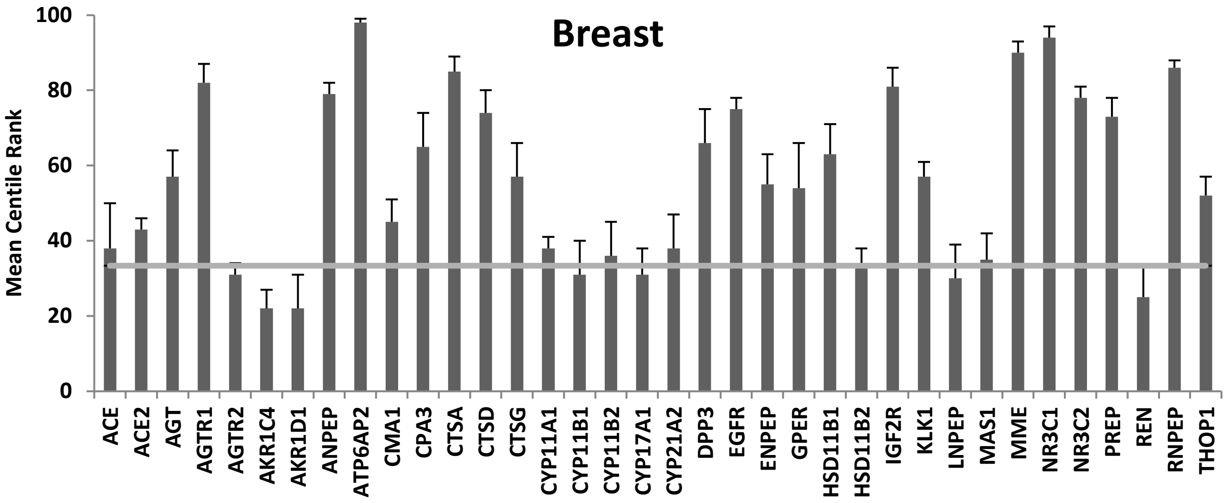

Subcutaneous Adipose
(9, 474, 8)

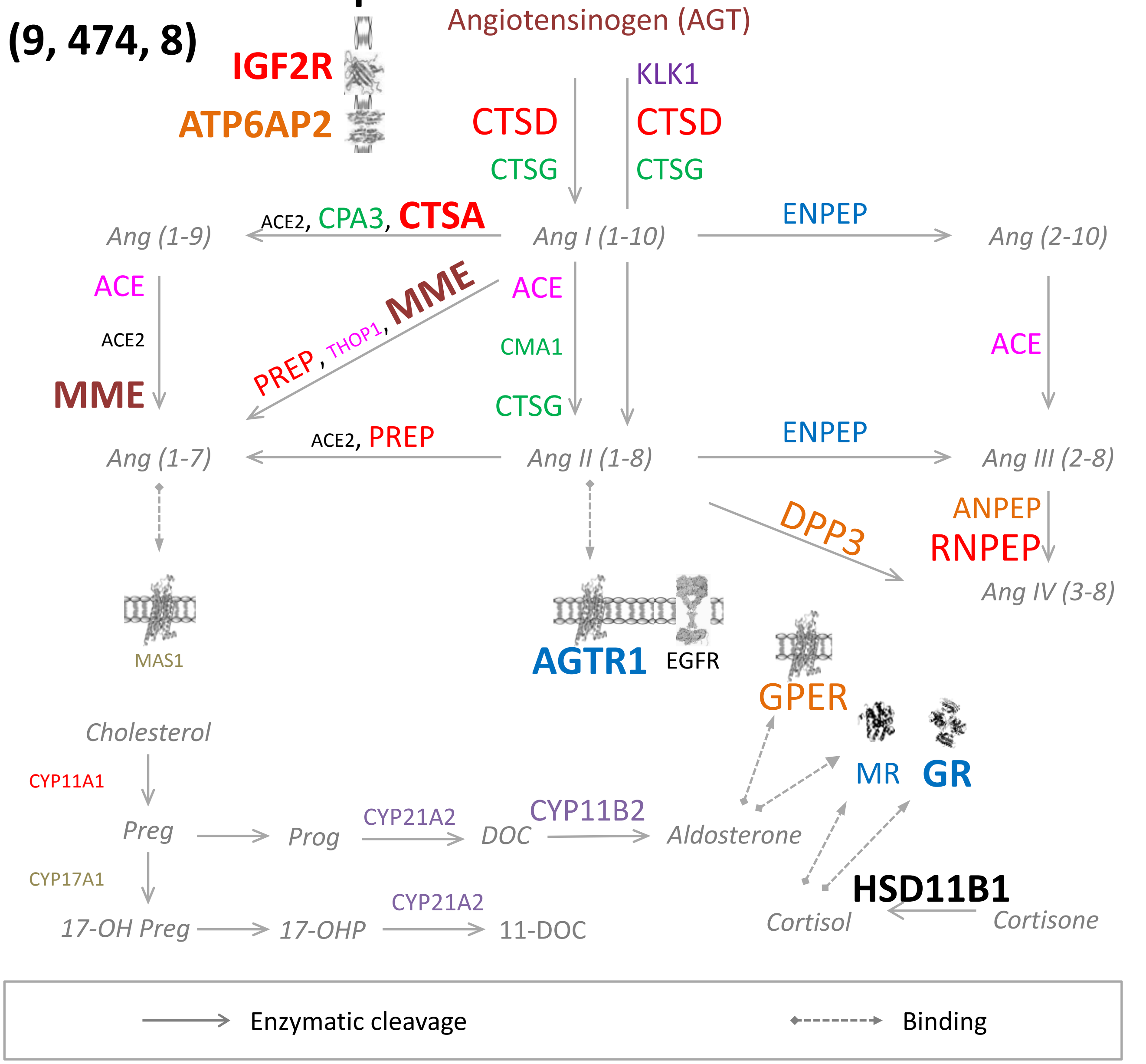

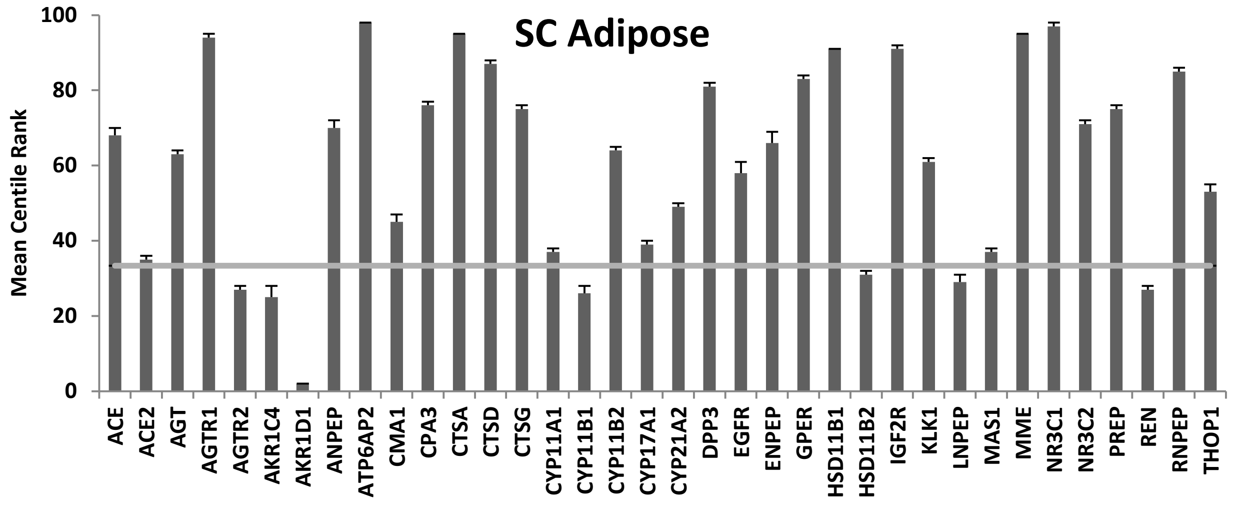

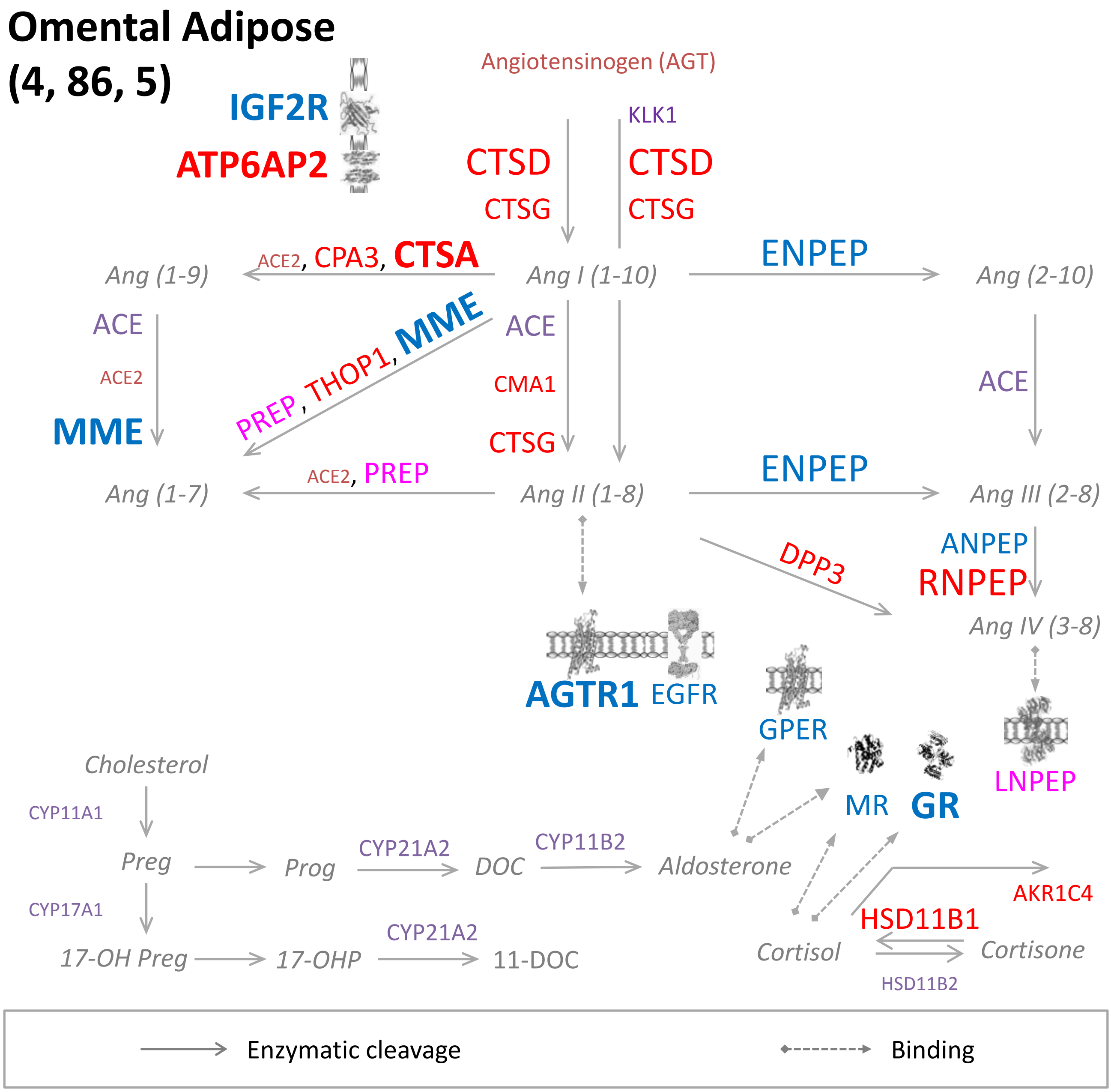
Omental Adipose
(4, 86, 5)
IGF2R
ATP6AP2
Angiotensinogen (AGT)
KLK1
CTSD
CTSG
CTSD
CTSG
Ang (1-9)
ACE2, CPA3, CTSA
Ang I (1-10)
ENPEP
Ang (2-10)
ACE
ACE2
MME
PREP, THOP1, MME
ACE
CMA1
CTSG
ACE
Ang (1-7)
ACE2, PREP
Ang II (1-8)
ENPEP
Ang III (2-8)
DPP3
ANPEP
RNPEP
Ang IV (3-8)
AGTR1
EGFR
GPER
MR
GR
LNPEP
Cholesterol
CYP11A1
Preg
Prog
CYP21A2
DOC
CYP11B2
Aldosterone
CYP17A1
17-OH Preg
17-OHP
CYP21A2
11-DOC
AKR1C4
HSD11B1
Cortisol
Cortisone
HSD11B2
Enzymatic cleavage
Binding

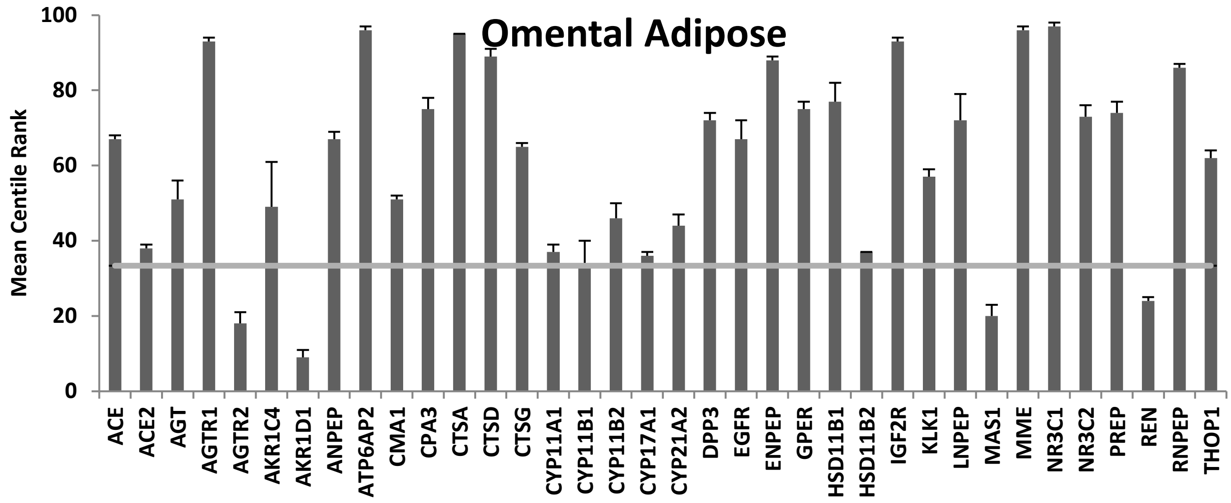
Omental Adipose
Mean Centile Rank
0
20
40
60
80
100
ACE
ACE2
AGT
AGTR1
AGTR2
AKR1C4
AKR1D1
ANPEP
ATP6AP2
CMA1
CPA3
CTSA
CTSD
CTSG
CYP11A1
CYP11B1
CYP11B2
CYP17A1
CYP21A2
DPP3
EGFR
ENPEP
GPER
HSD11B1
HSD11B2
IGF2R
KLK1
LNPEP
MAS1
MME
NR3C1
NR3C2
PREP
REN
RNPEP
THOP1

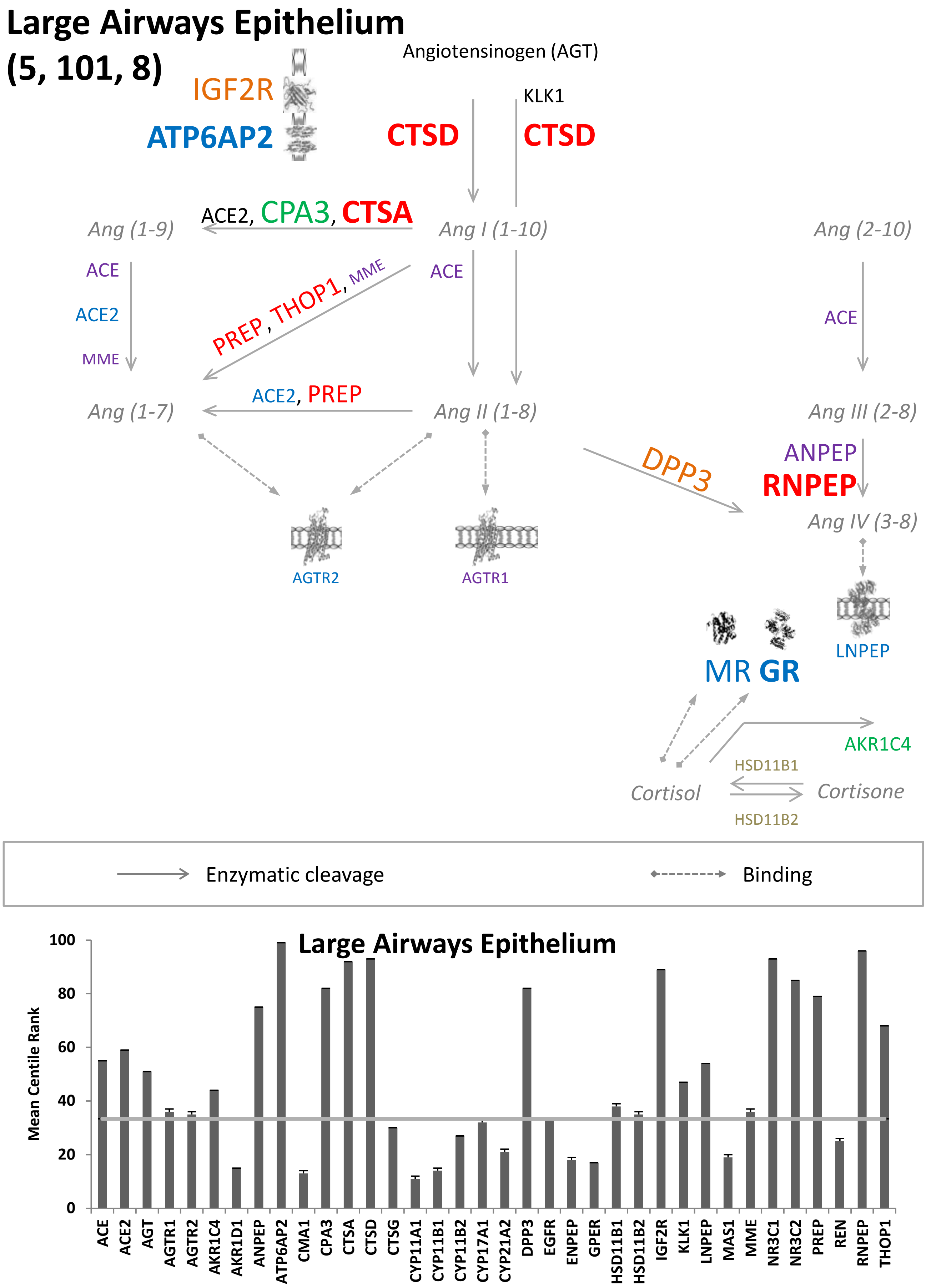
Large Airways Epithelium
(5, 101, 8)
Angiotensinogen (AGT)
KLK1
IGF2R
ATP6AP2
CTSD
CTSD
ACE2, CPA3, CTSA
Ang (1-9)
Ang I (1-10)
Ang (2-10)
ACE
ACE2
MME
PREP, THOP1, MME
ACE
ACE
ACE2, PREP
Ang (1-7)
Ang II (1-8)
Ang III (2-8)
DPP3
ANPEP
RNPEP
Ang IV (3-8)
AGTR2
AGTR1
LNPEP
MR GR
AKR1C4
HSD11B1
Cortisol
Cortisone
HSD11B2
Enzymatic cleavage
Binding
Large Airways Epithelium
Mean Centile Rank
100
80
60
40
20
0
ACE
ACE2
AGT
AGTR1
AGTR2
AKR1C4
AKR1D1
ANPEP
ATP6AP2
CMA1
CPA3
CTSA
CTSD
CTSG
CYP11A1
CYP11B1
CYP11B2
CYP17A1
CYP21A2
DPP3
EGFR
ENPEP
GPER
HSD11B1
HSD11B2
IGF2R
KLK1
LNPEP
MAS1
MME
NR3C1
NR3C2
PREP
REN
RNPEP
THOP1

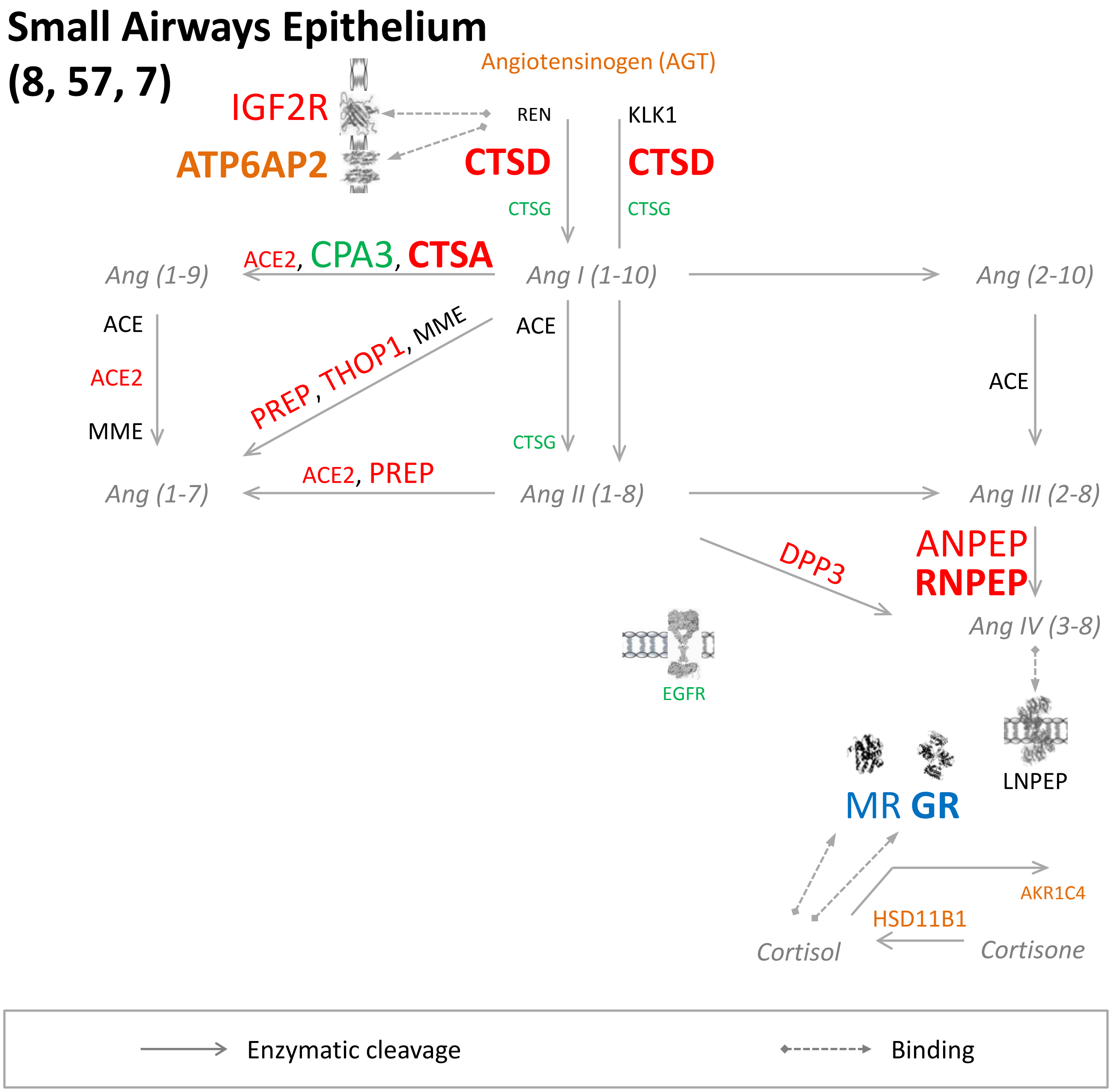
Small Airways Epithelium
(8, 57, 7)
Angiotensinogen (AGT)
IGF2R
ATP6AP2
REN
KLK1
CTSD
CTSD
CTSG
CTSG
ACE2, CPA3, CTSA
Ang (1-9)
Ang I (1-10)
Ang (2-10)
ACE
ACE2
MME
PREP, THOP1, MME
ACE
CTSG
ACE
ACE2, PREP
Ang (1-7)
Ang II (1-8)
Ang III (2-8)
DPP3
ANPEP
RNPEP
Ang IV (3-8)
EGFR
MR GR
LNPEP
AKR1C4
HSD11B1
Cortisol
Cortisone
Enzymatic cleavage
Binding

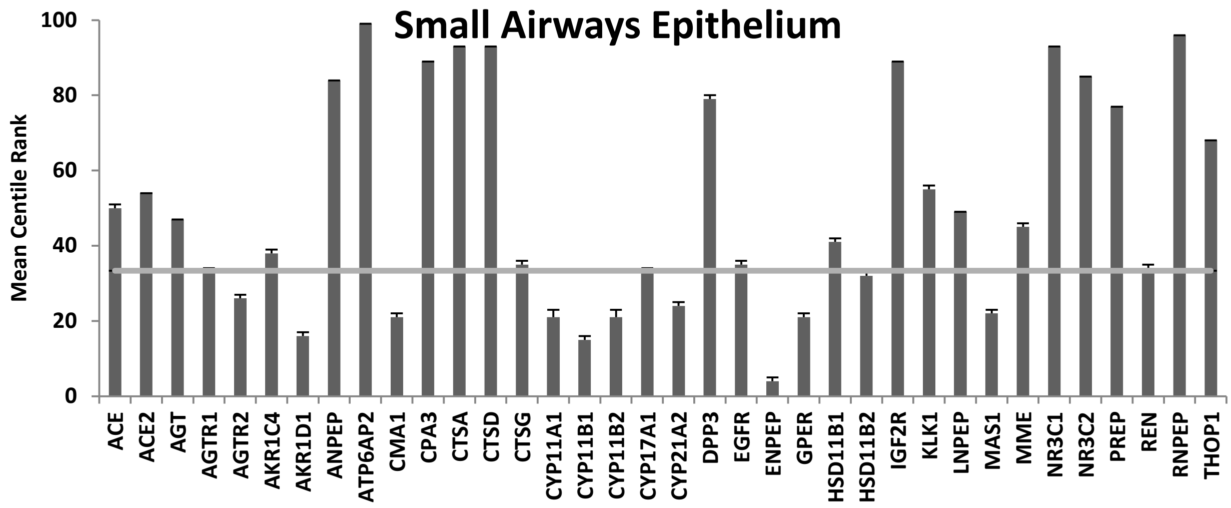
Small Airways Epithelium
Mean Centile Rank
100
80
60
40
20
0
ACE
ACE2
AGT
AGTR1
AGTR2
AKR1C4
AKR1D1
ANPEP
ATP6AP2
CMA1
CPA3
CTSA
CTSD
CTSG
CYP11A1
CYP11B1
CYP11B2
CYP17A1
CYP21A2
DPP3
EGFR
ENPEP
GPER
HSD11B1
HSD11B2
IGF2R
KLK1
LNPEP
MAS1
MME
NR3C1
NR3C2
PREP
REN
RNPEP
THOP1

Esophagus
(3, 83, 6)

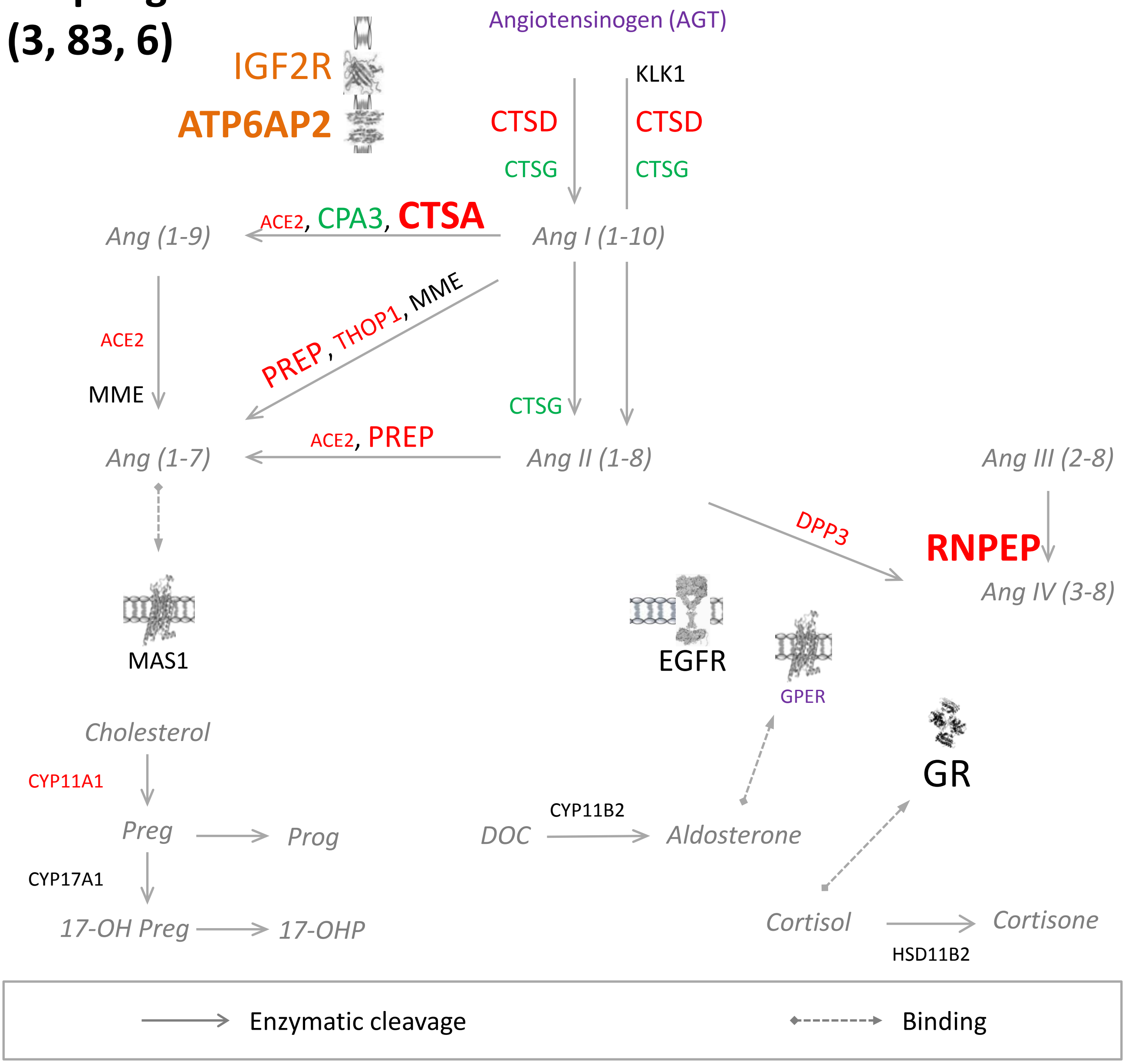

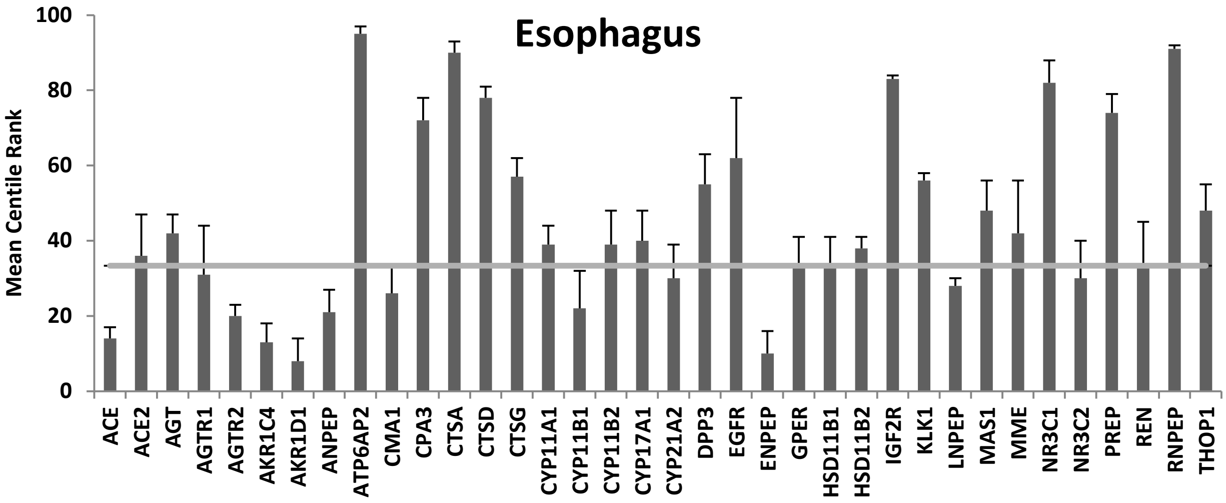

Lymphocytes (6, 184, 7)

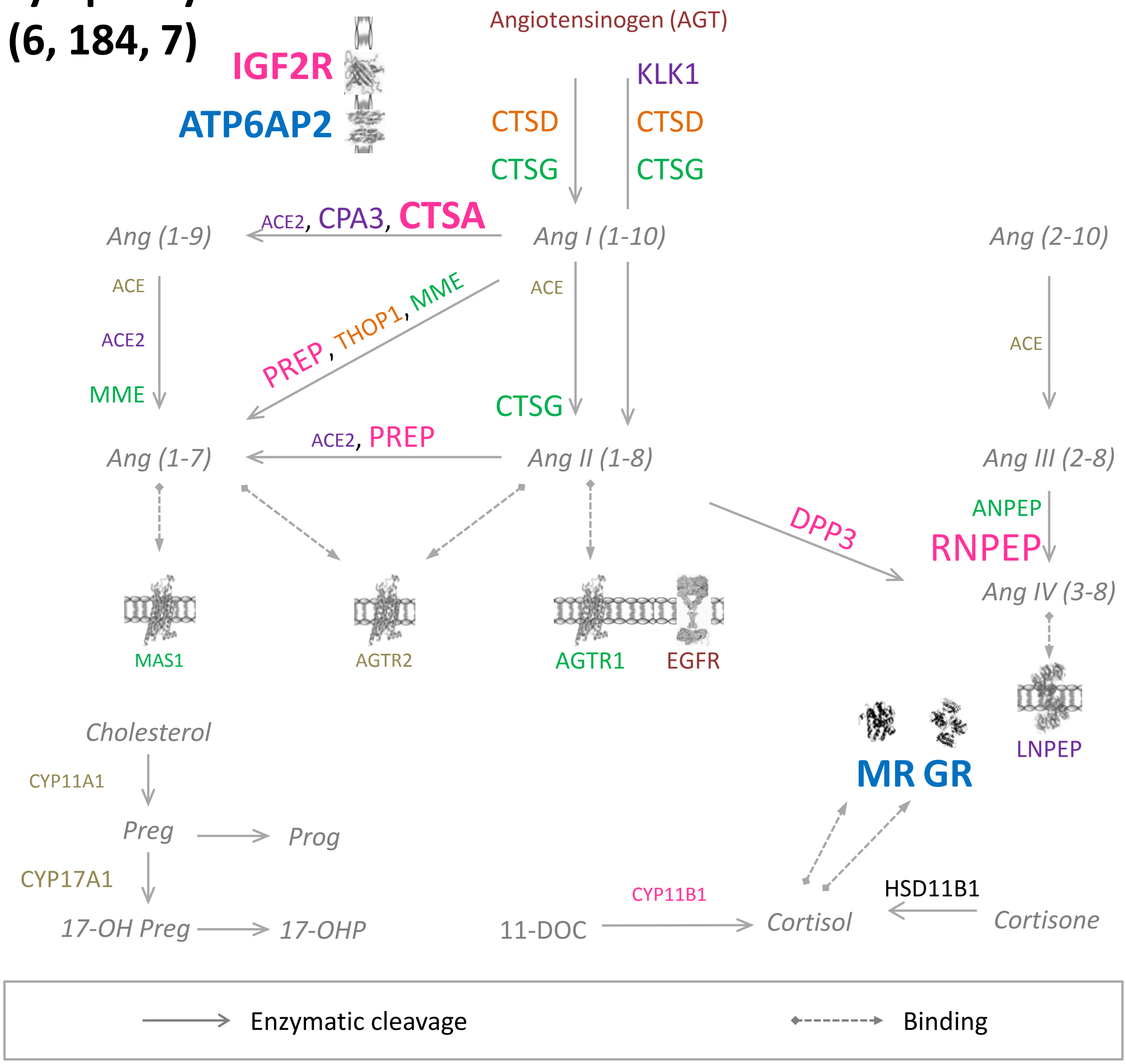

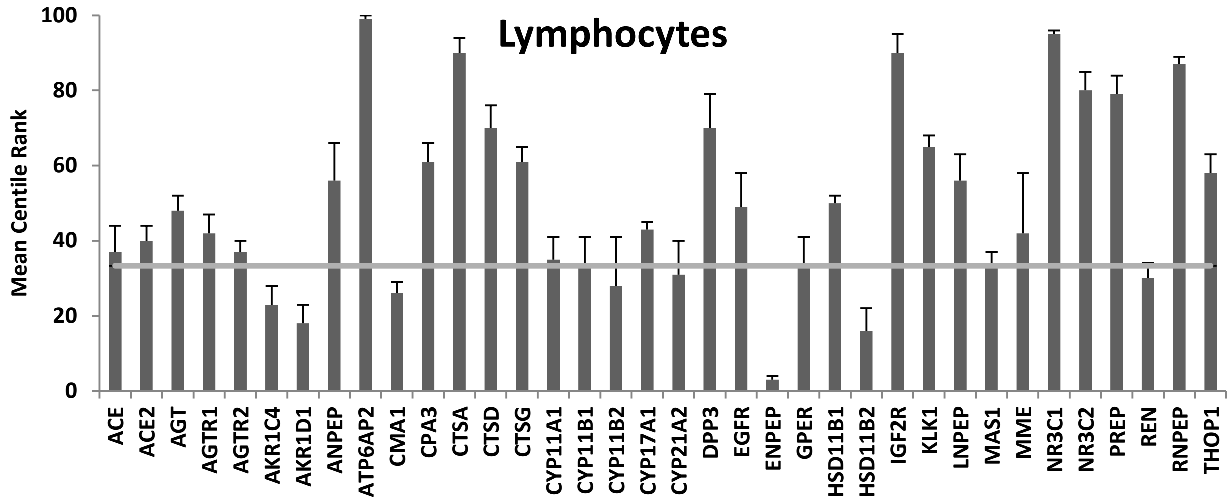

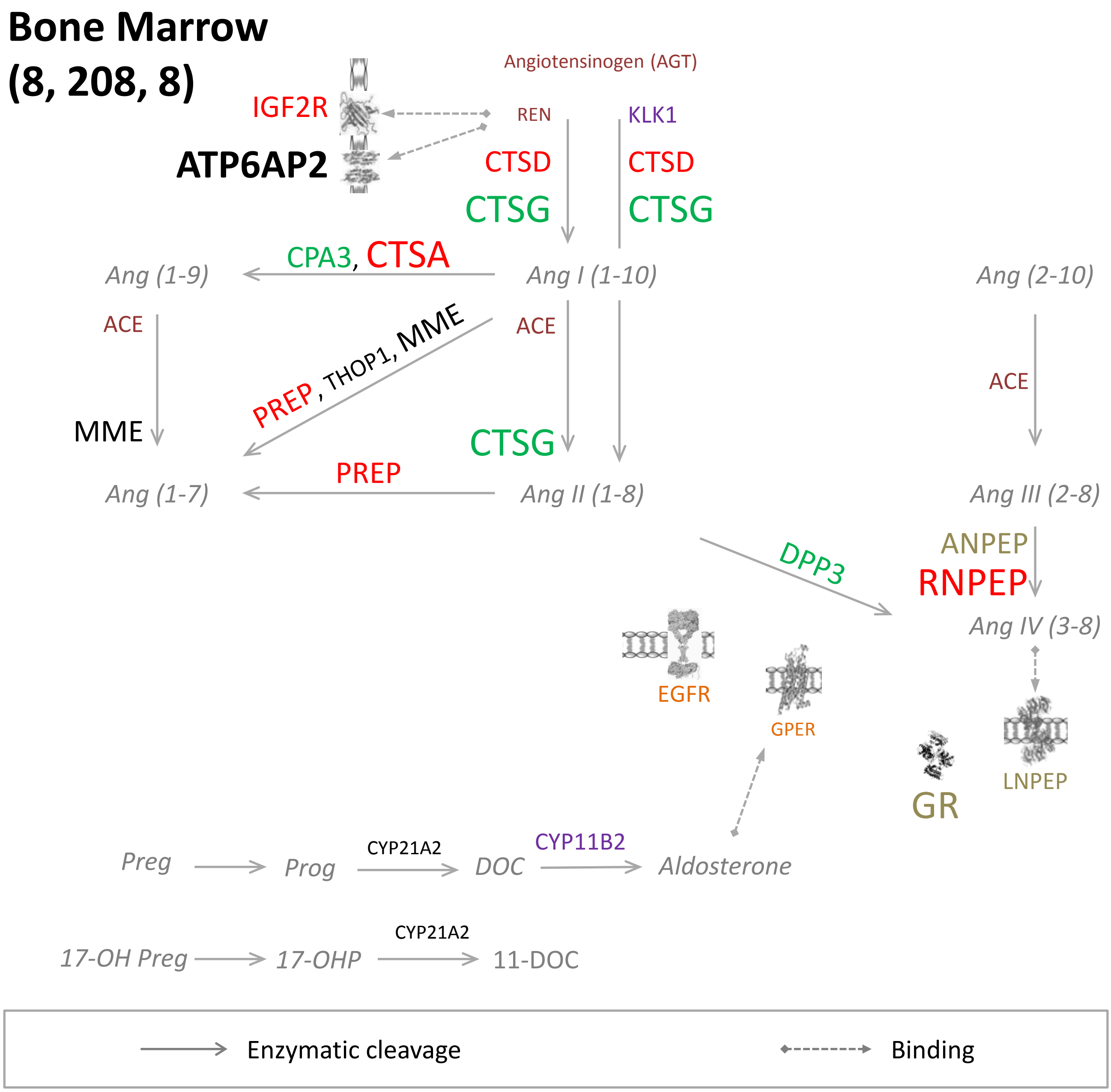

Bone Marrow
(8, 208, 8)
Angiotensinogen (AGT)
IGF2R
ATP6AP2
REN
KLK1
CTSD
CTSG
CTSD
CTSG
CPA3, CTSA
Ang (1-9)
Ang I (1-10)
Ang (2-10)
ACE
ACE
ACE
PREP, THOP1, MME
MME
CTSG
PREP
Ang (1-7)
Ang II (1-8)
Ang III (2-8)
DPP3
ANPEP
RNPEP
Ang IV (3-8)
EGFR
GPER
GR
LNPEP
Preg
Prog
CYP21A2
DOC
CYP11B2
Aldosterone
17-OH Preg
17-OHP
CYP21A2
11-DOC
Enzymatic cleavage
Binding

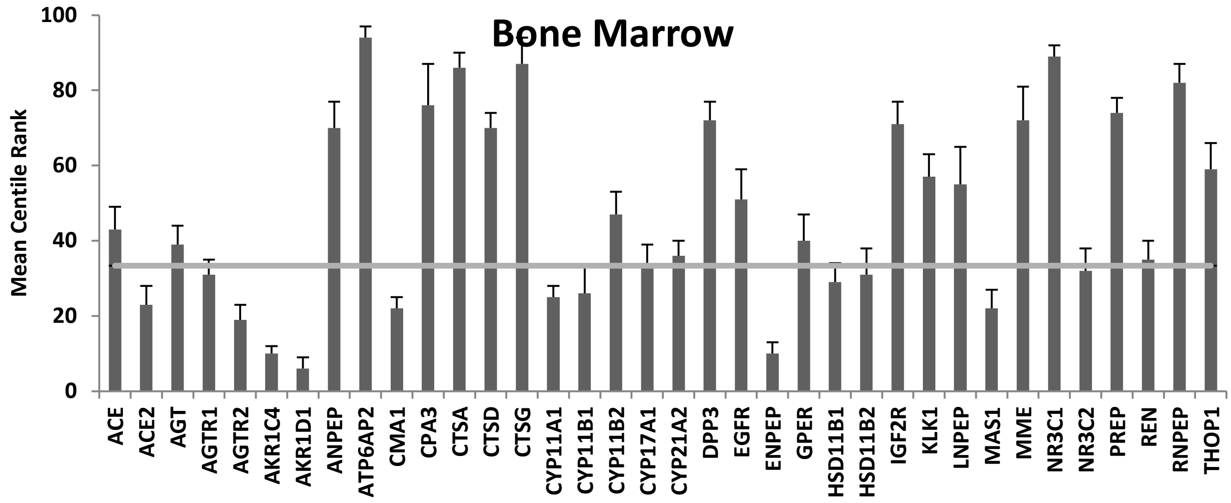

Bone Marrow
Mean Centile Rank
0
20
40
60
80
100
ACE
ACE2
AGT
AGTR1
AGTR2
AKR1C4
AKR1D1
ANPEP
ATP6AP2
CMA1
CPA3
CTSA
CTSD
CTSG
CYP11A1
CYP11B1
CYP11B2
CYP17A1
CYP21A2
DPP3
EGFR
ENPEP
GPER
HSD11B1
HSD11B2
IGF2R
KLK1
LNPEP
MAS1
MME
NR3C1
NR3C2
PREP
REN
RNPEP
THOP1

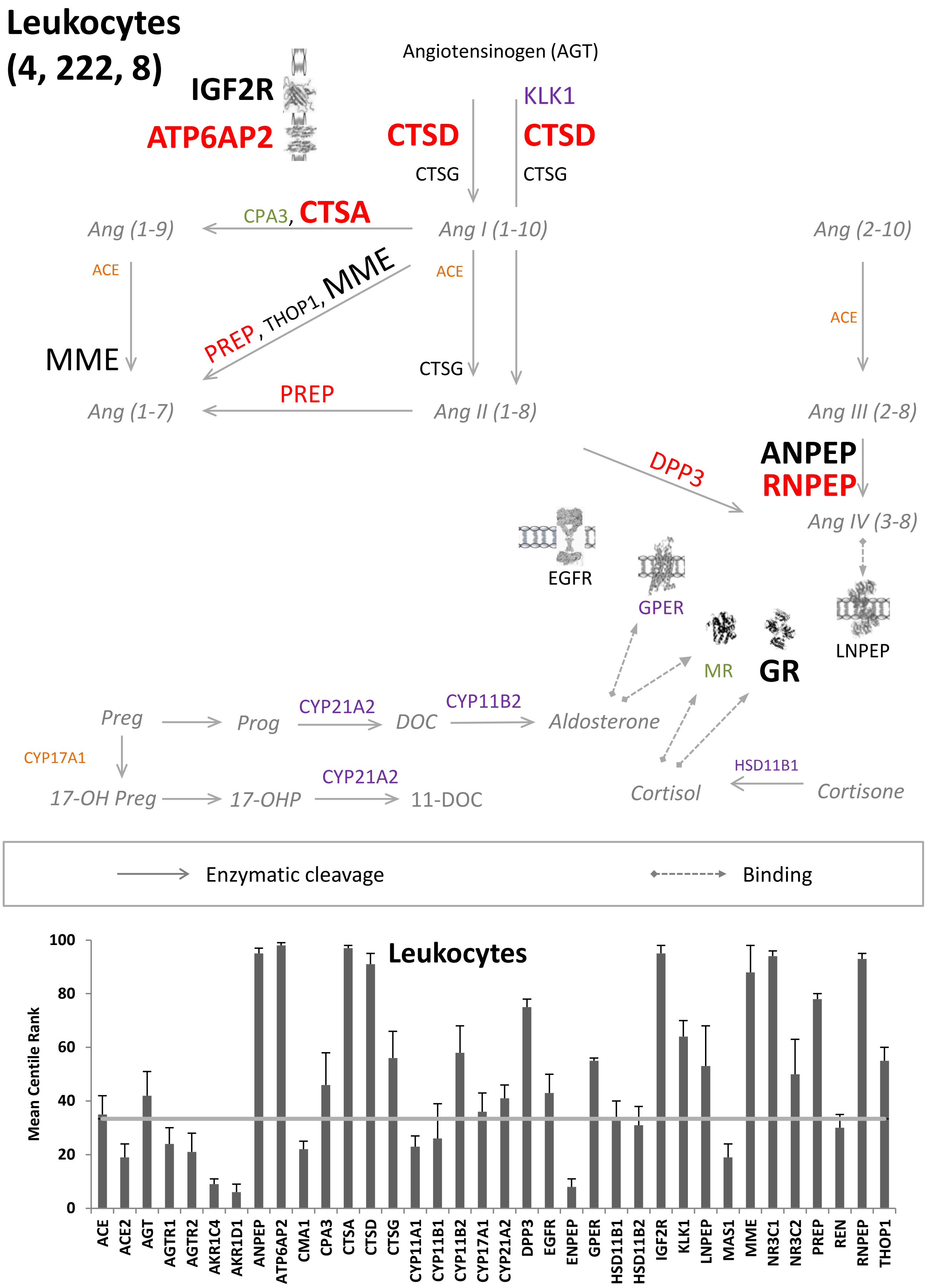

Leukocytes
(4, 222, 8)
IGF2R
ATP6AP2
Angiotensinogen (AGT)
KLK1
CTSD
CTSG
CTSD
CTSG
CPA3, CTSA
Ang (1-9)
Ang I (1-10)
Ang (2-10)
ACE
PREP, THOP1, MME
ACE
ACE
MME
CTSG
Ang (1-7)
PREP
Ang II (1-8)
Ang III (2-8)
DPP3
ANPEP
RNPEP
Ang IV (3-8)
EGFR
GPER
MR
GR
LNPEP
Preg
Prog
CYP21A2
DOC
CYP11B2
Aldosterone
CYP17A1
17-OH Preg
17-OHP
CYP21A2
11-DOC
Cortisol
HSD11B1
Cortisone
Enzymatic cleavage
Binding
Leukocytes
Mean Centile Rank
0
20
40
60
80
100
ACE
ACE2
AGT
AGTR1
AGTR2
AKR1C4
AKR1D1
ANPEP
ATP6AP2
CMA1
CPA3
CTSA
CTSD
CTSG
CYP11A1
CYP11B1
CYP11B2
CYP17A1
CYP21A2
DPP3
EGFR
ENPEP
GPER
HSD11B1
HSD11B2
IGF2R
KLK1
LNPEP
MAS1
MME
NR3C1
NR3C2
PREP
REN
RNPEP
THOP1

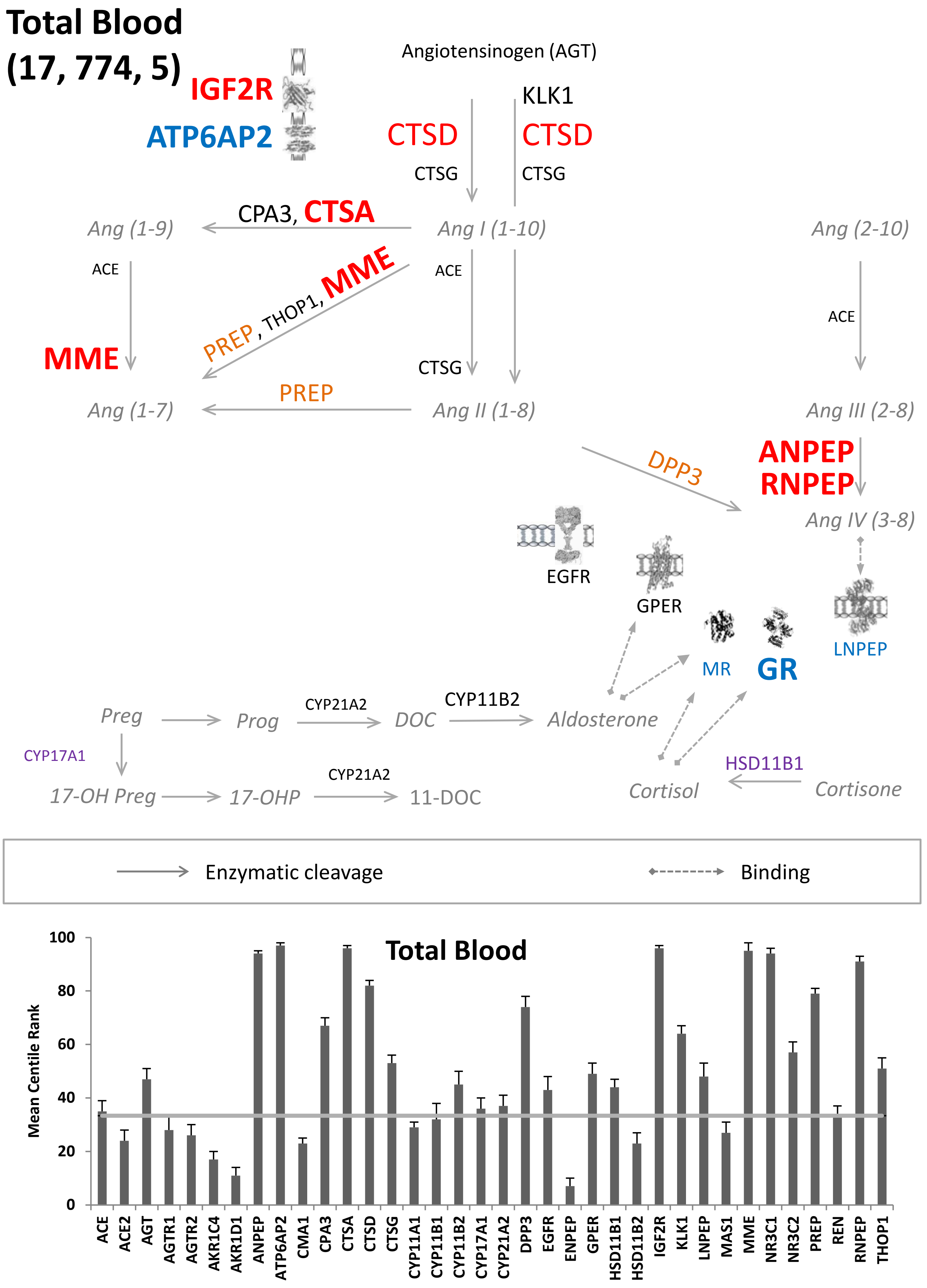
Total Blood
(17, 774, 5)
IGF2R
ATP6AP2
Angiotensinogen (AGT)
KLK1
CTSD
CTSD
CTSG
CTSG
CPA3, CTSA
Ang (1-9)
Ang I (1-10)
Ang (2-10)
ACE
ACE
ACE
PREP, THOP1, MME
MME
CTSG
PREP
Ang (1-7)
Ang II (1-8)
Ang III (2-8)
DPP3
ANPEP
RNPEP
Ang IV (3-8)
EGFR
GPER
MR
GR
LNPEP
Preg
Prog
CYP21A2
DOC
CYP11B2
Aldosterone
CYP17A1
17-OH Preg
17-OHP
CYP21A2
11-DOC
Cortisol
HSD11B1
Cortisone
Enzymatic cleavage
Binding
Total Blood
Mean Centile Rank
100
80
60
40
20
0
ACE
ACE2
AGT
AGTR1
AGTR2
AKR1C4
AKR1D1
ANPEP
ATP6AP2
CMA1
CPA3
CTSA
CTSD
CTSG
CYP11A1
CYP11B1
CYP11B2
CYP17A1
CYP21A2
DPP3
EGFR
ENPEP
GPER
HSD11B1
HSD11B2
IGF2R
KLK1
LNPEP
MAS1
MME
NR3C1
NR3C2
PREP
REN
RNPEP
THOP1

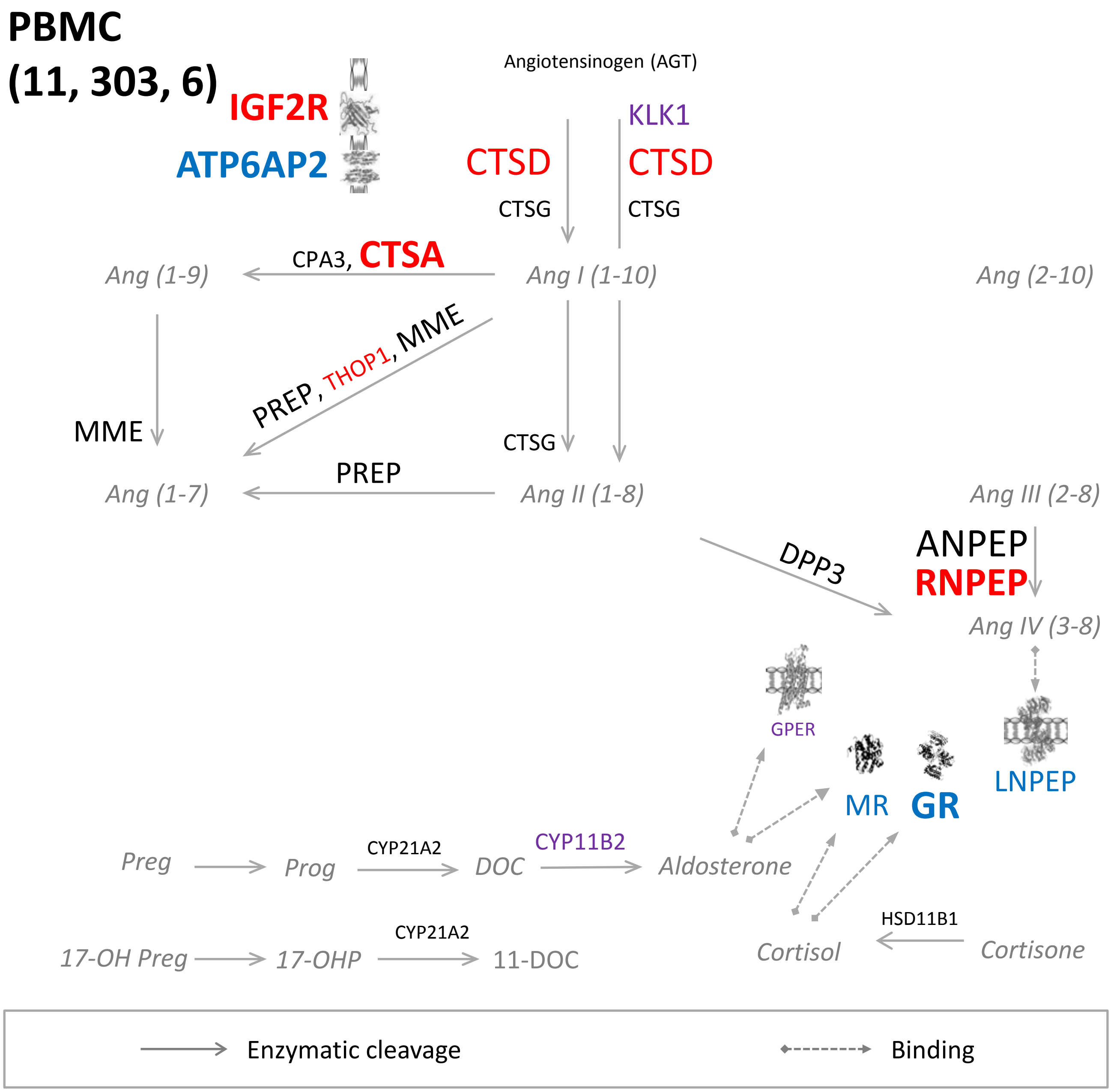
PBMC
(11, 303, 6)
IGF2R
ATP6AP2
Angiotensinogen (AGT)
KLK1
CTSD
CTSD
CTSG
CTSG
CPA3, CTSA
Ang (1-9)
Ang I (1-10)
Ang (2-10)
PREP, THOP1, MME
MME
CTSG
Ang (1-7)
PREP
Ang II (1-8)
Ang III (2-8)
DPP3
ANPEP
RNPEP
Ang IV (3-8)
GPER
MR
GR
LNPEP
Preg
Prog
CYP21A2
DOC
CYP11B2
Aldosterone
17-OH Preg
17-OHP
CYP21A2
11-DOC
Cortisol
HSD11B1
Cortisone
Enzymatic cleavage
Binding

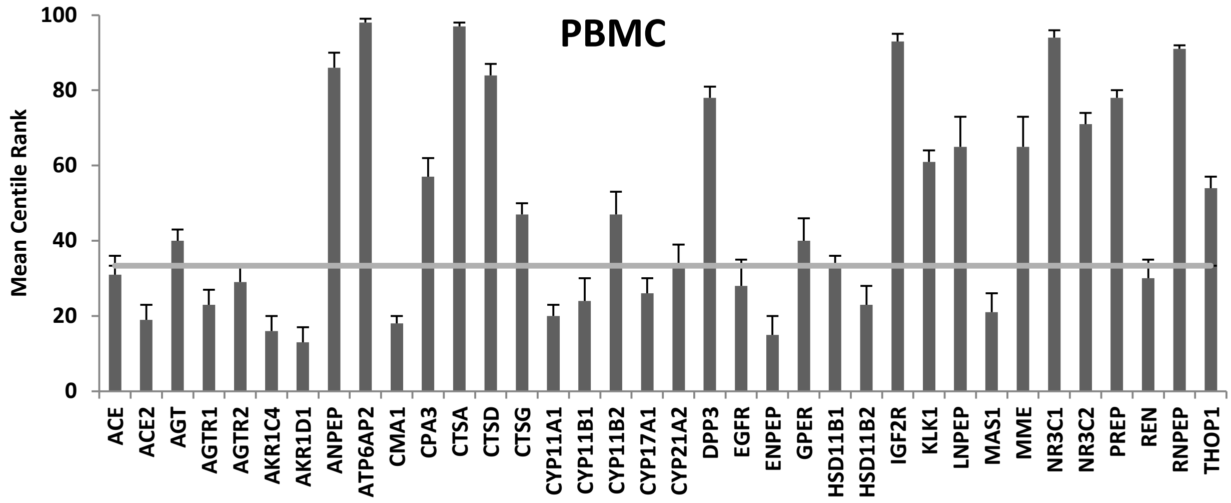
PBMC
Mean Centile Rank
100
80
60
40
20
0
ACE
ACE2
AGT
AGTR1
AGTR2
AKR1C4
AKR1D1
ANPEP
ATP6AP2
CMA1
CPA3
CTSA
CTSD
CTSG
CYP11A1
CYP11B1
CYP11B2
CYP17A1
CYP21A2
DPP3
EGFR
ENPEP
GPER
HSD11B1
HSD11B2
IGF2R
KLK1
LNPEP
MAS1
MME
NR3C1
NR3C2
PREP
REN
RNPEP
THOP1
